# Psychological interventions for harmful gambling: Systematic review and network meta-analysis

**DOI:** 10.1556/2006.2025.00445

**Published:** 2026-09-16

**Authors:** Odette Megnin-Viggars, Ifigeneia Mavranezouli, Beatrice C Downing, Nicky J Welton, Anja Fricke, Jennifer Francis, Tim Reeves, Stephen Pilling, Henrietta Bowden-Jones, Barbara J Sahakian, Mohammad S Rahman, Robert Dawson, André Geel, Matthew Gaskell

**Affiliations:** 1 Centre for Outcomes Research and Effectiveness, Research Department of Clinical, Educational, and Health Psychology, University College London, London, UK; 2 Population Health Sciences, Bristol Medical School, University of Bristol, Bristol, UK; 3 National Institute for Health and Care Excellence, Manchester, UK; 4 NHS England, National Advisor on Gambling Harms, UK, National Problem Gambling Clinic & National Centre for Gaming Disorders, London, UK; 5 Department of Psychiatry, University of Cambridge, Cambridge, UK; 6 NHS Nottingham and Nottinghamshire Integrated Care Board, Nottingham, UK; 7 NECA, Tyne and Wear, UK; 8 Central and North West London NHS Foundation Trust, London, UK; 9 NHS Northern Gambling Service, Leeds & York Partnership NHS Foundation Trust, Leeds, UK

**Keywords:** gambling, psychological interventions, network meta-analysis, clinical guidelines, systematic review

## Abstract

**Background and aims:**

Gambling causes harms for the individual, their families and wider society. Psychological interventions have been shown to improve gambling severity, but their relative effectiveness is not well understood.

**Methods:**

Systematic review and network meta-analysis (NMA) of psychological interventions for harmful gambling. Outcomes were gambling symptom severity and gambling frequency (expressed as standardized mean differences [SMDs]).

**Results:**

We included 52 randomized controlled trials (RCTs), 9013 participants and 14 intervention classes. For gambling symptom severity, group CBT was the most effective intervention (SMD −1.09, 95% credible intervals [95%CrIs] −1.75 to −0.44) followed by individual CBT (SMD −0.56, 95%CrIs −1.07 to −0.07). Waitlist showed increased symptom severity compared with no treatment (SMD 0.96, 95%CrIs 0.47 to 1.46). All other intervention effects were characterized by uncertainty. For gambling frequency, all active interventions showed similar effects to treatment as usual.

**Discussion:**

Included studies were of low or very low quality and there was moderate-to-high heterogeneity for the gambling symptom severity NMA. However, threshold analysis suggested that conclusions based on the NMA were robust to potential biases and sampling variation in the included evidence. Bias adjustment analyses did not show evidence of bias associated with small studies or industry funding.

**Conclusions:**

Group CBT shows clinically important benefits and should be regarded as the best intervention for harmful gambling. For individuals who decline group CBT, or where this is not possible or suitable, individual CBT shows the next best evidence of benefit.

## Introduction

Harmful gambling is an important global public health issue. Recent prevalence estimates suggest that 8.7% of adults have engaged in any risk gambling in the past 12 months globally, and 1.4% have engaged in problematic gambling, with particularly high levels of harmful gambling associated with online formats (Tran et al., 2024). Gambling causes a range of harms for the individual, their families and for the wider society, including financial harms, relationship problems, emotional distress, loss of employment, and involvement in criminal activity (Langham et al., 2016). Additionally, there appear to be health inequalities in gambling related harms, with some vulnerable groups, for instance, those with lower socioeconomic status or younger age groups, being more at risk of experiencing harms (Raybould, Larkin, & Tunney, 2021). People who experience harmful gambling have high rates of coexisting mental health problems that may precede or be secondary to harmful gambling, in particular, depression, anxiety disorders and substance use disorders (Dowling et al., 2015). Moreover, harmful gambling has been identified as a significant risk factor for suicidality, even after controlling for psychiatric comorbidity (Wardle, John, Dymond, & McManus, 2020).

Assessment of psychological and pharmacological interventions for gambling disorder in randomized controlled trials (RCTs) and the development of clinical guidelines for the management of gambling-related harms have been highlighted as key research priorities to guide national policies (Bowden-Jones et al., 2022). Cognitive behavioral therapy (CBT) is the most common psychological intervention for treating gambling problems (Menchon, Mestre-Bach, Steward, Fernandez-Aranda, & Jimenez-Murcia, 2018). Findings from meta-analyses of RCTs suggest that psychological interventions may improve gambling symptom severity overall (Eriksen et al., 2023; Ribeiro, Afonso, & Morgado, 2021), with CBT found to have the largest evidence base and a large, statistically significant, effect (Eriksen et al., 2023; Ribeiro et al., 2021). Evidence suggests high effects for group psychological interventions in reducing gambling symptom severity, but significant effects have also been found for individual, face-to-face and remote psychological interventions (Eriksen et al., 2023). However, the systematic reviews to date have been limited to (minimal) pairwise comparisons of these interventions, and the relative effectiveness of different psychological interventions is not known.

Network meta-analysis has the advantage of combining evidence from RCTs on multiple interventions in connected networks and providing internally coherent estimates of relative intervention effectiveness for any pair of interventions in the network including compared to a common reference condition (e.g. no treatment). Unlike pairwise meta-analysis, network meta-analysis can incorporate direct evidence (interventions that have been compared against each other in one or more trials) and indirect evidence (interventions that have not been compared against each other in a trial but the difference between them is inferred from other comparisons) to increase the precision and robustness of results (Caldwell, Ades, & Higgins, 2005). In this study, we updated a network meta-analysis (NMA) that examined the relative effectiveness of psychological interventions for harmful gambling, which we had originally conducted to inform the development of national guidance on gambling-related harms in England, published by the National Institute for Health and Care Excellence (NICE) (National Institute for Health and Care Excellence, 2025).

## Methods

### Search strategy and selection criteria

A systematic review of RCTs of psychological interventions for the treatment of harmful gambling was undertaken according to Preferred Reporting Items for Systematic reviews and Meta-Analyses (PRISMA) guidelines for NMAs (Hutton et al., 2015). The study protocol was registered on PROSPERO (CRD42022356147). The following electronic databases were searched for RCTs published between 2000 and March 2026 (studies published up to November 2022 were included in the original NMA): Applied Social Science Index and Abstracts (ASSIA); Cumulative Index to Nursing and Allied Health Literature (CINAHL); Cochrane Central Register of Controlled Trials (CENTRAL); Cochrane Database of Systematic Reviews (CDSR); Embase; Emcare; Epistemonikos; Health Management Information Consortium (HMIC); International Health Technology Assessment (INAHTA); Medline and Medline In-Process; PsycInfo; Social Care Online; Social Policy and Practice; Social Sciences Citation Index (Supplementary material S1). Additional search methods included checking reference lists of systematic reviews (identified through the electronic database search), and sources of grey literature, for example, Kings Fund reports, Campbell Collaboration, Gambling Research Exchange Ontario, Australian Gambling Research Centre, and Gambling and Addictions Research Centre.

The review included RCTs of psychological or psychosocial interventions for adults aged at least 18 years who were currently experiencing harmful gambling according to the Diagnostic and Statistical Manual of Mental Disorders (DSM), the International Classification of Diseases (ICD), or similar criteria, or by scoring above threshold on a validated gambling symptom severity scale, or self-identifying as experiencing harmful gambling and seeking treatment. Comorbid conditions such as depression, anxiety and substance-use disorders were not a criterion for exclusion, although trials including people with bipolar disorder where gambling behaviour only occurred during manic episodes were excluded. Eligible interventions included cognitive and behavioural interventions and related techniques, other psychotherapeutic interventions for harmful gambling, trauma-informed interventions for addiction, self-help, digital interventions and helplines, life and social skills-based interventions, family, systemic and significant other interventions, community and peer support interventions. Pharmacological interventions were eligible for inclusion if they were used in combination with a psychological/psychosocial intervention or as a control condition Trials of population-level interventions, non-English language papers, conference abstracts, dissertations, and trial registry protocols were excluded from the review.

Titles and abstracts of identified studies were screened by two reviewers for inclusion against pre-defined protocol criteria, until inter-rater reliability of at least 90% agreement was observed. Initially 10% of references were double-screened; as inter-rater agreement was over 90%, the remaining references were screened by one reviewer. For studies considered relevant after title/abstract screening, the full text paper was acquired and checked for eligibility. Disagreements in study inclusion were resolved through discussion between the two reviewers, and consultation with a senior reviewer if necessary.

### Data analysis

Study characteristics and outcome data were extracted into a standardized template created in Excel and all data extraction and risk of bias assessment were double-coded. From each included study, we extracted: country, population characteristics, intervention details, outcome data, source of funding, and risk of bias. The latter was assessed using the Cochrane Risk-of-Bias tool (Higgins et al., 2019), which considered the following domains: adequacy of randomisation, blinding, attrition, selective reporting bias, and other bias.

There were a number of interventions that shared common characteristics, and some of these interventions had been tested on small numbers of participants and their effects were characterized by considerable uncertainty. For this reason, the NMAs utilized class models and interventions were grouped according to common theoretical structure and methodological approach, using relevant information extracted from the included studies. Interventions within a class were expected to have similar (but not necessarily identical) effects. Psychological intervention classes included in the NMAs were as follows: CBT individual; CBT group; behavioural therapies individual; counselling individual; motivational interviewing; self-help (with no or minimal support); guided self-help; couple interventions; twelve step group programme. Controls included no treatment, treatment as usual (TAU), attention placebo, waitlist, and selective serotonin reuptake inhibitors (SSRIs).

Two outcomes were analyzed using NMA techniques: gambling symptom severity (measured as a change in a symptom severity score from baseline to treatment endpoint) and gambling frequency. Change from baseline scores were less commonly reported than final scores in the included studies but were preferred as they control for baseline imbalance, which was an issue in some of the studies. Change from baseline scores were calculated using baseline and endpoint means and assuming a correlation of 0.5 to estimate the standard deviation in the change from baseline (Higgins et al., 2019). Studies reported intervention effects across a number of validated gambling symptom severity scales (Supplementary material S2), none of which were consistently used in all RCTs. Some studies reported the average number of diagnostic criteria (DSM-IV pathological gambling or DSM-5 gambling disorder criteria) met before and after treatment as a measure of gambling severity, either in addition to or instead of a scale score. In order to include studies that only reported the number of diagnostic criteria item scores, these were treated as a proxy for a gambling severity score and pooled with scale symptom severity scores in the NMA of gambling symptom severity. This was considered reasonable, as all studies reporting the number of diagnostic criteria used 9 or 10 item criteria and this was judged to be sufficiently large to treat as a continuous scale. For studies reporting two or more different continuous gambling severity scales, or a scale score and the number of diagnostic criteria met, a pooled study-specific effect was calculated to avoid arbitrarily prioritizing one of these outcomes (Daly, Welton, Dias, Anwer, & Ades, 2021). Due to the range of different severity scales, including the number of diagnostic criteria, used to measure change scores, relative effects on gambling symptom severity between treatments were synthesized using standardized mean differences (SMDs) (rather than mean differences on a single symptom severity scale, which could have been used if all studies had used the same scale to measure symptom severity) (Higgins et al., 2019). The gambling frequency NMA also synthesized frequency measures using SMDs due to between-study variability in the scale of frequency measures as a result of differences in the length of follow-up times, units (days or sessions), and populations (baseline frequency).

NMAs were conducted within a Bayesian framework using Markov Chain Monte Carlo simulation techniques implemented in OpenBUGS 3.2.3 (Lunn, Jackson, Best, Thomas, & Spiegelhalter, 2012) for the gambling symptom severity outcome and WinBUGS 1.4.3 (Spiegelhalter, Thomas, Best, & Lunn, 2003) for the gambling frequency outcome. The WinBUGS code for SMDs from (Dias, Ades, Welton, Jansen, & Sutton, 2018) was adapted to incorporate the pooling across outcomes for studies reporting two different measures of symptom severity (Supplementary material S3). Both fixed and random class models were fitted. The former assumes that intervention effects within a class are identical, and the latter assumes that similar intervention effects within a class are distributed around the class mean. Fixed and random study effects models were also fitted for each class model. Model fit was assessed using the posterior mean residual deviance and the Deviance Information Criterion (DIC).

For each NMA, mean relative effects (SMDs) were estimated between intervention classes with 95% credible intervals (CrIs). Mean ranks (and 95% CrIs) were also estimated for each intervention class, with a rank of 1 indicating the best intervention class. Results were interpreted in terms of ‘evidence of effect’, which was determined by 95% CrIs not crossing the line of no effect relative to a reference class, which was no treatment.

### Inconsistency checks

A core NMA assumption is that the distribution of effect modifiers across the included studies is similar, so that intervention A would be expected to have similar relative effects in intervention A versus intervention B trials as it would have in intervention A versus intervention C trials (transitivity assumption). The validity of this assumption was tested by measuring statistical heterogeneity, and the consistency between direct and indirect evidence. To assess consistency, the fit of the NMA model was compared to an ‘inconsistency’ or unrelated mean effects (UME) model, equivalent to having separate, unrelated, meta-analyses for every pairwise contrast (code provided in Supplementary material S3) (Dias et al., 2013). The consistency and inconsistency models were compared based on their posterior residual deviance and DIC. Deviance (dev-dev) plots were also produced to plot the posterior mean deviance for individual data points in the inconsistency model against their posterior mean deviance in the NMA model. Points lying below the line of equality indicated data points contributing to inconsistency.

### Sensitivity and bias-adjusted analyses

There was a high level of attrition in the studies included, and, where possible, results from an Intention-To-Treat (ITT) analysis that adjusted for attrition were extracted from the studies. However, for the base-case analysis, where results were only reported for those with post-intervention follow-up data (Complete Case (CC) analysis), ITT data were imputed by assuming that, for participants with missing endpoint values, these would equal their baseline values (Baseline Observation Carried Forward (BOCF)). Sensitivity analyses were conducted using (i) only studies reporting ITT data, and (ii) only studies reporting CC data (with no imputation). Potential bias associated with industry-funded studies was explored in two ways. Firstly by only including studies where it was clear they did not receive industry funding. Secondly by fitting bias-adjusted models that included a covariate effect if a study reported receiving industry funding or if industry funding was unclear. Given that the NMAs included a significant number of studies with small sample sizes, bias-adjusted models were also fitted to adjust for bias associated with small study size effects by including a covariate effect for 1/N, where N is the sample size. In all bias-adjusted models, potential bias was assumed in comparisons of active interventions versus inactive control (see Supplementary material S3 for code for bias-adjusted models).

### Threshold analysis

Threshold analysis was used to assess the robustness of the NMA-based conclusions and guideline recommendations to potential study limitations, including high risk of bias or sampling variation (Caldwell et al., 2016; Phillippo et al., 2018, 2019). Threshold analysis quantifies how much the evidence would need to change before the conclusion would change and what the revised conclusion would be.

## Results

### Studies and treatments

The systematic literature search identified 6888 potentially eligible records after removal of duplicates (8029), of which 55 publications reporting on 52 RCTs were included in the NMA (Fig. 1). Supplementary material S4 reports included study characteristics with full references. 164 publications assessed for eligibility at full-text were excluded (Supplementary material S5).

**Fig. 1.**
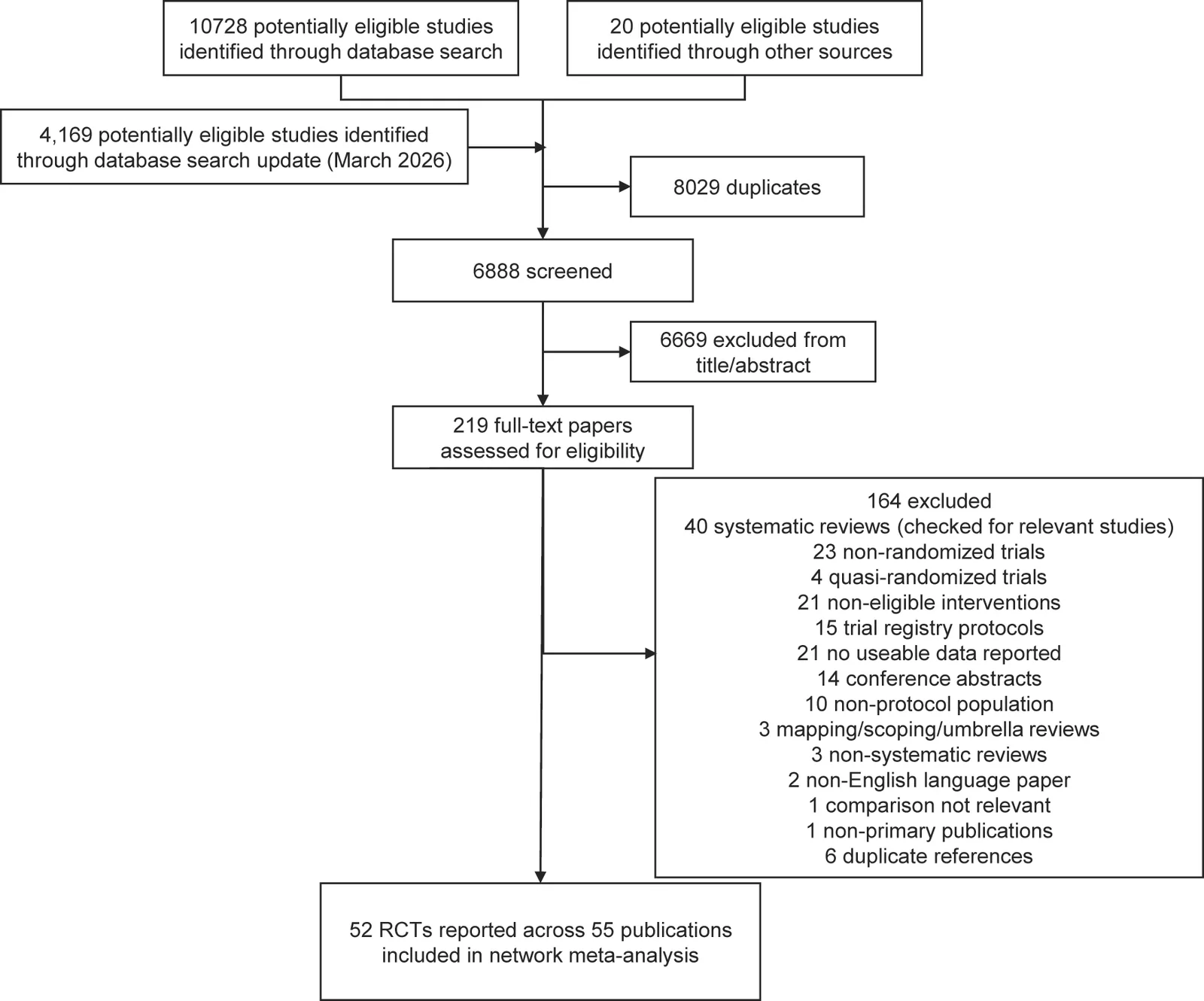
Study selection

### Risk of bias assessment

The quality of the individual studies included in the NMAs was rated as low or very low (Supplementary material S6), mainly due to risk of bias stemming from lack of blinding, poor reporting of randomization procedures, or high attrition rates. This impacted on the quality of the NMAs.

### NMA model fit statistics

Based on model fit statistics (Supplementary material S7), fixed class effect models were used for both gambling symptom severity and gambling frequency NMAs, indicating no evidence of heterogeneity between interventions within a class. For the gambling symptom severity NMA, model fit statistics supported using a random study effects model. For the gambling frequency NMA, there was a small advantage in DIC for fixed study-specific effects, but this difference was slight and model estimates were similar, suggesting either model would be appropriate. For consistency with the symptom severity NMA, the random study effect with fixed class model was chosen for both outcomes.

### Gambling symptom severity

#### Treatment outcomes

The NMA for gambling symptom severity included 46 RCTs, 14 intervention classes, and 6564 participants (see Supplementary material S8). The respective network is shown in Fig. 2. Results for each intervention class relative to no treatment for gambling symptom severity are shown in Table 1 and Fig. 3 as a forest plot. All active intervention classes except pure self-help showed some evidence of benefit compared to no treatment, and ranked in a higher position relative to treatment as usual (TAU) and waitlist. However, for most intervention classes the results were characterized by uncertainty as indicated by wide 95% CrIs around the mean effect, and also by overlapping rankings with very wide 95% CrIs. Group CBT had the highest effect among all intervention classes, followed by individual CBT. These were the only intervention classes that showed evidence of effect (95% Crls that did not cross the line of no effect) compared with no treatment. Waitlist showed increased symptom severity compared with no treatment, with the 95% CrIs not crossing the line of no effect.

**Fig. 2.**
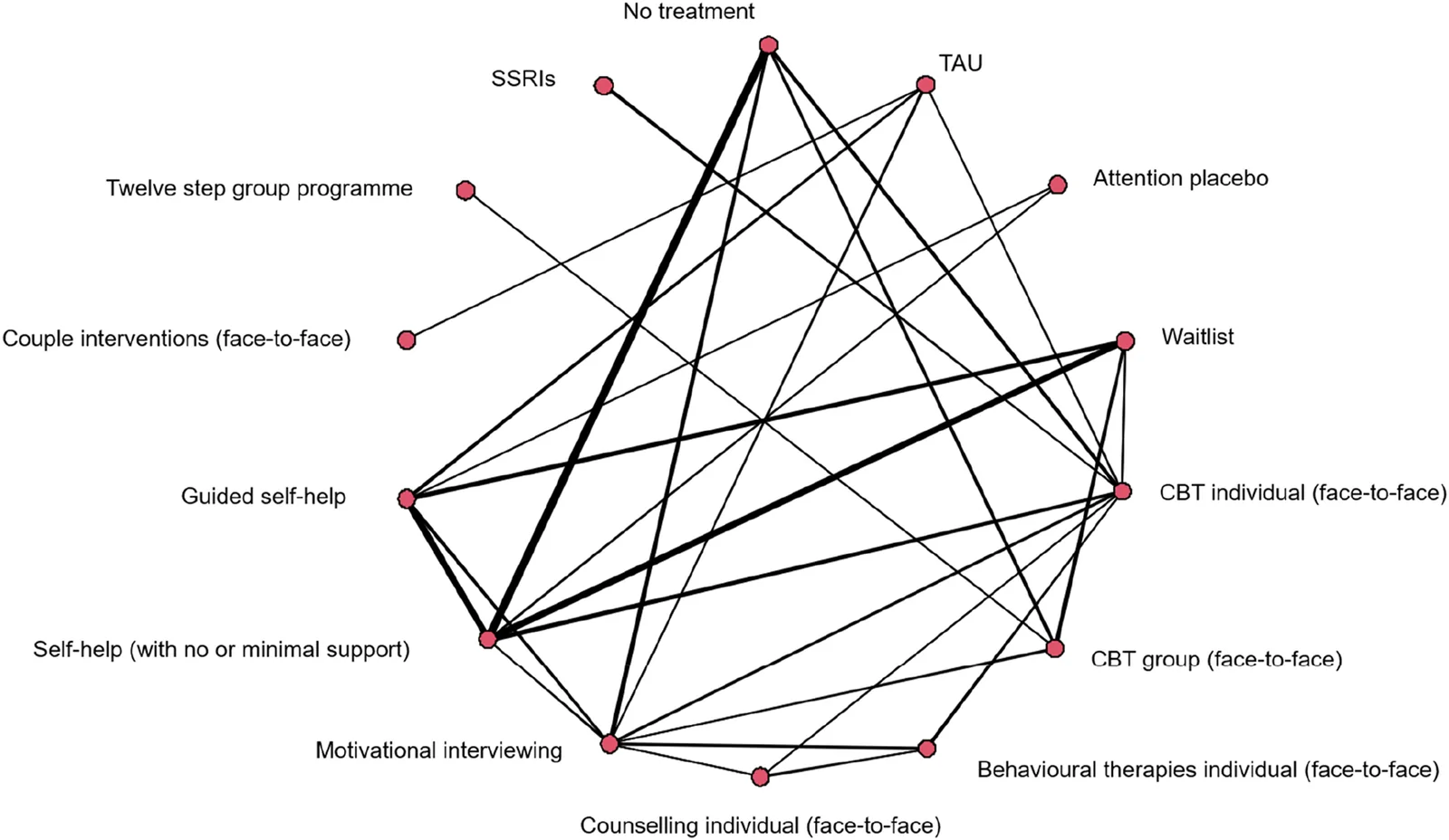
Network of intervention classes for gambling symptom severity. Each intervention class is represented by a circle. The size of each circle (treatment node) is proportional to the number of participants in each intervention class. The lines connecting intervention classes show the available direct (head-to-head) trial comparisons. The width of lines is proportional to the number of trials in which each direct comparison is made. CBT: cognitive behavioural therapy; SSRIs: selective serotonin reuptake inhibitors; TAU: treatment as usual

**Table 1.** Gambling symptom severity results – full dataset: posterior standardised mean difference (SMD) of all treatments versus no treatment

Intervention class	*N* rand	**SMD vs no treatment (mean, 95% CrIs**)	Rank (mean, 95% CrIs)
CBT group	121	**−1.09 (−1.75 to −0.44)**	2.04 (1 to 5)
CBT individual	592	**−0.56 (−1.07 to −0.07)**	4.49 (2 to 8)
Behavioural therapies individual	136	−0.59 (−1.41 to 0.23)	4.70 (1 to 11)
Couple interventions	8	−0.48 (−2.22 to 1.28)	6.12 (1 to 14)
Counselling individual	76	−0.43 (−1.51 to 0.65)	6.06 (1 to 13)
SSRIs	15	−0.46 (−1.92 to 0.99)	6.12 (1 to 14)
Twelve step group programme	11	−0.38 (−1.91 to 1.11)	6.66 (1 to 14)
Motivational interviewing	303	−0.30 (−0.84 to 0.24)	6.69 (3 to 11)
Guided self-help	994	−0.06 (−0.56 to 0.44)	8.87 (5 to 13)
No treatment	771	Reference	9.61 (5 to 13)
Attention placebo	271	0.04 (−0.75 to 0.82)	9.59 (3 to 13)
Self-help (with no or minimal support)	2,555	0.01 (−0.36 to 0.37)	9.73 (6 to 13)
TAU	153	0.16 (−0.69 to 0.99)	10.50 (4 to 13)
Waitlist	558	**0.96 (0.47 to 1.46)**	13.83 (13 to 14)

CBT: cognitive behavioural therapy; CrI: credible intervals; K arms: number of arms; N rand: number randomised; SMD: standardised mean difference; SSRIs: selective serotonin reuptake inhibitors; TAU: treatment as usual.

Intervention classes ordered from best to worst, according to mean rankings. Negative effect values indicate a favourable outcome compared with no treatment. Results where 95% CrI do not cross the no effect line are shown in bold.

**Fig. 3.**
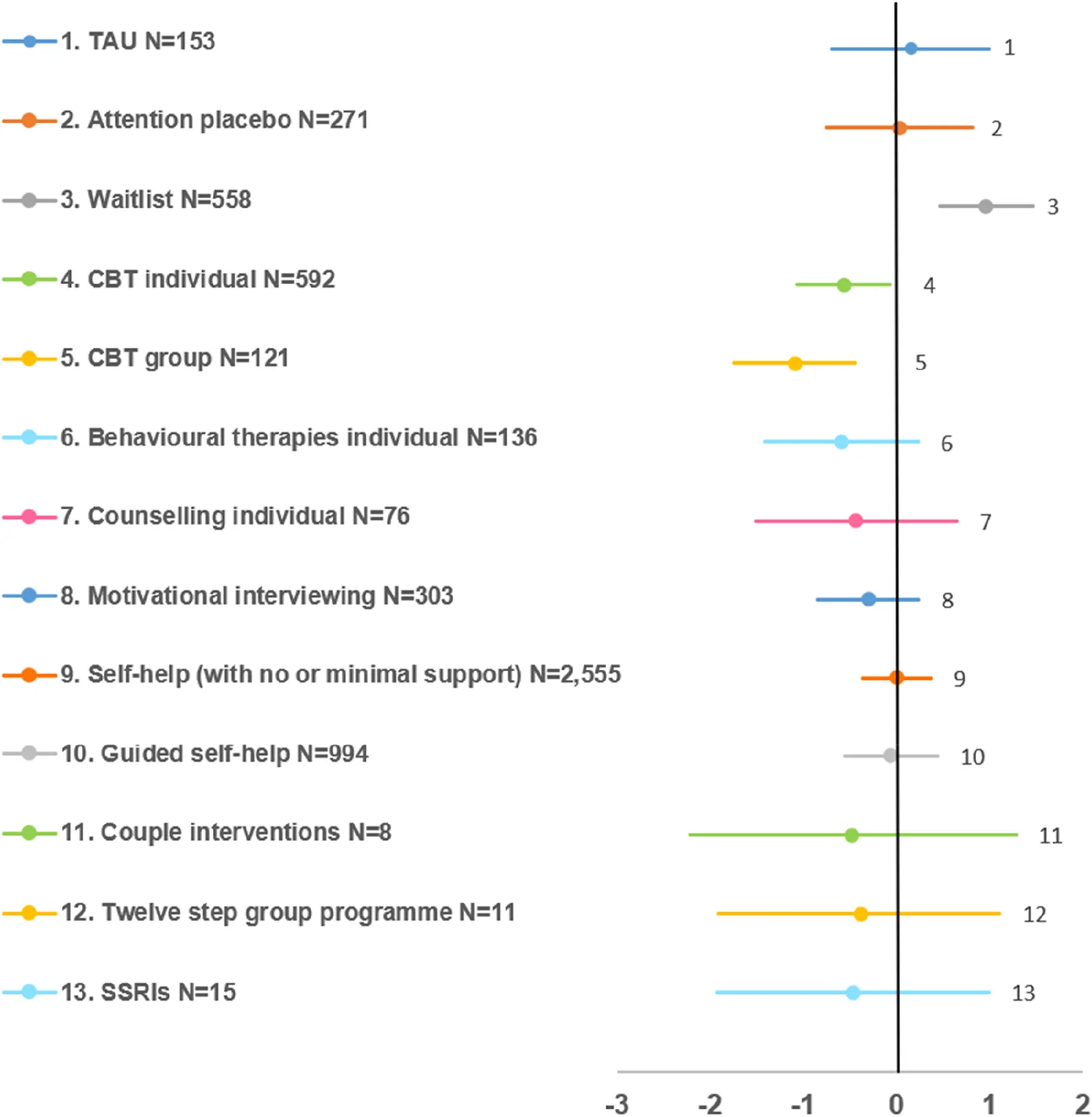
Gambling symptom severity forest plot – full dataset. Standardized mean difference of each intervention class versus no treatment (*N* = 681). Vertical axis represents the effect of no treatment. Values on the left side of the vertical axis indicate better effect compared with no treatment. CBT: cognitive behavioural therapy; SSRIs: selective serotonin reuptake inhibitors; TAU: treatment as usual

Sensitivity analyses (Supplementary material S9) were informed by more limited data but were broadly consistent with the base-case analysis. After using complete cases data, group CBT was the only class showing evidence of effect versus no treatment; group CBT, individual CBT, individual behavioural therapies and guided self-help showed evidence of effect versus no treatment in the ITT analysis, in which self-help with no or with minimal support was the only other active intervention class present. Active intervention classes either were not present or showed uncertain effects in the sensitivity analysis that included only non-industry funded trials.

In the base-case analysis, comparisons between active intervention classes indicated evidence of effect for group CBT versus motivational interviewing, group CBT versus guided self-help, group CBT versus self-help with no or with minimal support, and individual CBT versus self-help with no or with minimal support. Relative effects between other active intervention classes were characterized by uncertainty (Supplementary material S10).

#### Inconsistency, heterogeneity and bias-adjusted analyses

There was no evidence of global inconsistency as seen by comparing model fit for the inconsistency and NMA models (Supplementary material S7). However, the dev-dev plot showed that the direct evidence from one study (Ladouceur et al., 2001) found a stronger benefit of CBT individual compared with waitlist than that seen from the indirect evidence. The effect was in the same direction and 95% CrIs did not contain 0 for both, but the magnitude of the effect of CBT individual should be interpreted with caution.

Heterogeneity was found to be moderate-to-high, and this was attributed to construct differences across gambling symptom severity scales, and between gambling symptom severity scales and DSM criteria, which were synthesized in the analysis. Bias analyses testing for bias resulting from small study size and bias associated with funding source (any industry funding/unclear funding) did not show evidence of such bias (Supplementary material S7).

### Gambling frequency

#### Treatment outcomes

The NMA on gambling frequency included fewer studies with 23 RCTs, 11 intervention classes, and 4422 participants (Supplementary material S11). Supplementary materials S12 to S16 include the network plot, the NMA results versus no treatment, the forest plots versus no treatment, results of sensitivity analyses, and results for all pairs of intervention classes for the gambling frequency outcome. All active intervention classes showed a benefit compared to no treatment, with evidence of efficacy (95% CrIs not crossing the line of no effect) shown for individual behavioural therapies, individual CBT, motivational interviewing, guided self-help and self-help with no or minimal support. Waitlist had the same effect as no treatment. However, all active intervention classes showed similar (or somewhat lower) effects compared to TAU.

#### Inconsistency, heterogeneity and bias-adjusted analyses

Model fit statistics were similar for the gambling frequency outcome as for gambling symptom severity, with inconsistency between direct and indirect evidence only found in the comparison between individual CBT versus waitlist where the benefit for individual CBT observed in one study (Ladouceur et al., 2001) was not seen in the indirect evidence (Supplementary material S7). Heterogeneity was very low for gambling frequency, likely due to the latter being a more straightforward measurement (compared with gambling symptom severity) with similar methods used across studies. Bias adjustment analyses did not show evidence of small study bias or bias associated with industry funding (Supplementary material S7).

### Threshold analysis

Threshold analysis on the gambling symptom severity outcome (Supplementary material S17) suggested that the conclusions of the NMA, which directly informed guideline recommendations, were robust to potential plausible changes in the evidence.

## Discussion

### Overview of findings

Our findings are broadly consistent with previous systematic reviews employing pairwise meta-analysis (Eriksen et al., 2023; Ribeiro et al., 2021) and confirm that psychological interventions can effectively reduce gambling symptom severity and gambling frequency. The results of our NMA base-case analysis on gambling symptom severity suggested that group CBT had the highest effect among all intervention classes relative to no treatment, followed by individual CBT. These were the only intervention classes that showed evidence of effect compared with no treatment. Results of sensitivity analyses were similar. Large effects of group therapy have been observed in other addiction disorders, where they have been attributed to potential benefits in promoting peer support, reducing stigma, enhancing communication and interpersonal skills, and practicing coping strategies with other group members (Substance Abuse and Mental Health Services Administration, 2021). Across the trials included in the NMA, group CBT was delivered in weekly sessions usually by two therapists trained in CBT. Treatment included components such as recognising internal triggers, facilitating participant awareness, recognition of negative emotions, cognitive correction, motivation, decision-making, and problem solving. A principal component of group CBT across most programmes was relapse prevention.

Regarding gambling frequency, any intervention class (including TAU) showed higher effects compared with no treatment (albeit with varying uncertainty), but all intervention classes had a similar effect and it was not possible to differentiate between them. It is noted that gambling frequency is only one aspect of gambling symptom severity; the latter may capture gambling-related characteristics such as gambling frequency and expenditure but is also directly linked with wider aspects of personal, social and life functioning as well as physical and psychological wellbeing. Therefore, a more intensive intervention may be required to reduce gambling symptom severity compared with reducing gambling frequency. This may explain why a few treatments were found to be effective in reducing gambling frequency but only group and individual CBT were shown to be effective in reducing gambling symptom severity. It is also possible that more evidence needs to be gathered to reduce the uncertainty currently characterizing observed treatment effects on gambling symptom severity, considering also the underlying heterogeneity around this outcome.

### Strengths and limitations

To our knowledge, this is the first time that psychological interventions for the treatment of harmful gambling have been concurrently assessed in network meta-analysis. Our results are consistent with findings of previously published systematic reviews and meta-analyses (Eriksen et al., 2023; Ribeiro et al., 2021), but our NMA has the advantage of allowing estimation of relative effects between all pairs of intervention classes for people experiencing harmful gambling (via direct and indirect comparisons), using all available RCT evidence without breaking the rules of randomization (Caldwell et al., 2005).

The quality of the individual studies included in the NMAs was rated as low or very low, mainly due to risk of bias associated with lack of blinding, poor reporting of randomization procedures, or high attrition rates. The quality and limitations of individual RCTs need to be considered when interpreting the results. There was also moderate-to-high heterogeneity for the gambling symptom severity outcome (very low heterogeneity was found for the gambling frequency outcome) that was mainly attributed to differences between measures that were pooled in the analysis. Nevertheless, the results of the threshold analysis suggested that the treatment recommendations made on the basis of the NMA for gambling symptom severity were robust to potential biases and sampling variation in the included evidence. There was also no evidence of significantly different results in the bias-adjusted NMA models or sensitivity analyses that explored bias due to small study sample size, industry funding or analysis method, and this increases our confidence in the NMA results.

### Implications for practice and need for further research

Results of this NMA update are very similar to the results of the original NMA (available in Supplementary material S18), conducted to inform national guidance on gambling-related harms in England (National Institute for Health and Care Excellence, 2025), but notably they strengthen the evidence on the effectiveness of individual CBT. Given the more limited evidence base on gambling frequency and the fact that the latter is only one aspect of gambling symptom severity, the gambling symptom severity outcome was prioritized over gambling frequency when making treatment recommendations. Based on the findings of our original NMA and the results of the cost-effectiveness analysis, updated results of which will be reported elsewhere (Mavranezouli et al., under review), the NICE guideline on gambling-related harms recommends that group CBT be offered to reduce gambling severity and frequency (National Institute for Health and Care Excellence, 2025).

The NICE guideline also recommends individual CBT as an option where group CBT is not possible (for example, there are no other people available to form a group), if group therapy is judged to be unsuitable, or where there is a patient preference for individual intervention (National Institute for Health and Care Excellence, 2025). The recommendation for offering individual CBT as an alternative intervention, in addition to the evidence of effectiveness for individual CBT, was further supported by evidence of a negative effect for waitlist compared with no treatment, which suggested that people experiencing harmful gambling presenting to services should receive effective treatment rather than be placed on a waiting list. The general paucity of the evidence was also noted as well as the need for future large-scale RCTs to strengthen the evidence base.

In line with other findings (Eriksen et al., 2023), the NMA showed that although motivational interviewing had some beneficial effects on gambling symptom severity versus no treatment, efficacy relative to no treatment was lower compared with high intensity interventions and was characterized by uncertainty. It is important to note that some of the CBT interventions tested in the RCTs included in the NMA had an initial session of motivational interviewing as part of the intervention. Given the potential role of motivational interviewing in encouraging people to seek treatment, even when people are undecided as to whether they want to change their gambling, the NICE guideline makes a weaker (‘consider’) recommendation for motivational interviewing, to strengthen people's confidence and commitment to change, or to encourage people who are unsure or have reservations about starting treatment (National Institute for Health and Care Excellence, 2025).

The evidence for some intervention classes that have been shown to be effective in comparable fields such as alcohol use disorder, for example, twelve step group programmes (Kelly, Humphreys, & Ferri, 2020), was so limited and characterized by such a high level of uncertainty, that these intervention classes were not considered when formulating recommendations. Future research that explores the effectiveness of these interventions for harmful gambling could expand the treatment options available. There is also very little known about the longer-term outcomes of psychological interventions, and further research is recommended on long-term effectiveness including effects on increasing recovery capital, and on the potential impact of psychological interventions on reducing rates of suicide and self-harm that have been shown to be higher in people experiencing harmful gambling. There is also a lack of evidence for interventions for people who experience harmful gambling with comorbid conditions, and the potential for effective treatment of harmful gambling to improve or resolve secondary comorbidities. This is an important gap given the high rate of comorbidity in people experiencing harmful gambling. However, it should be noted that comorbid conditions such as depression, anxiety and substance-use disorders were not a criterion for exclusion in this review.

## Conclusions

In conclusion, although further research is needed in order to address a number of gaps in our understanding, this systematic review and NMA allowed evidence to be synthesized across psychological interventions used to treat harmful gambling and showed that group CBT was most effective in reducing gambling symptom severity. This NMA directly informed national guidance in England, and should be of general interest globally, to help improve outcomes for people experiencing harmful gambling through the provision of effective treatment. Equally importantly, there is further and imperative need for global and transparent research on the epidemiology, risk, predictors, protective factors and measurement tools of gambling and gambling-related harms, in order to inform relevant national policies and initiatives that aim to reduce the prevalence and harms of gambling (Bowden-Jones et al., 2022; Czako et al., 2025).

## Supplementary materials

## Data Availability

The datasets analyzed during the current study are available from the corresponding author on reasonable request.
