# Supplementary material for: Psychological interventions for harmful gambling: Systematic review and network meta-analysis
Source: J Behav Addict. 2026 Sep 16;15(3):971–80. doi: 10.1556/2006.2025.00445 (PMC13678395; doi:10.1556/2006.2025.00445)
Supplement: Supplementary file 1 [file jba-15-971-s001.pdf]

SUPPLEMENTARY MATERIAL

Psychological interventions for harmful gambling: systematic review and network meta-analysis

Contents

SUPPLEMENTARY MATERIAL..1

SUPPLEMENTARY MATERIAL S1: Search strategies 3

SUPPLEMENTARY MATERIAL S2: Gambling symptom severity scales 23

SUPPLEMENTARY MATERIAL S3: WinBUGS codes 24

WinBUGS code for Symptom Severity, Random Study Effect, Random Class Effect Model...24

WinBUGS code for Symptom Severity, Random Study Effect, Fixed Class Effect Model...25

WinBUGS code for Frequency, Random Study Effect, Fixed Class Effect Model...27

WinBUGS code for Symptom Severity, Unrelated Mean Effects (Inconsistency) Model...29

WinBUGS code for Symptom Severity, Random Study Effect, Fixed Class Effect Model, Meta-Regression for Industry Funding 30

WinBUGS code for Symptom Severity, Random Study Effect, Fixed Class Effect Model, Meta-Regression for Sample Size...............................32

SUPPLEMENTARY MATERIAL S4: Included studies, study characteristics and references...35

SUPPLEMENTARY MATERIAL S5: Excluded studies, reason for exclusion and references...76

SUPPLEMENTARY MATERIAL S6: Risk of bias of individual studies included in the NMAs...92

SUPPLEMENTARY MATERIAL S7: Model fit statistics..94

Model fit statistics for the base case analysis for the outcome symptom severity, using the full dataset...94

Dev-dev plot for gambling severity, showing the posterior mean deviance for each data-point for the inconsistency model plotted against the NMA model (random study, fixed class effect model) 95

Model fit statistics for the base case analysis for the outcome gambling frequency, using the full dataset 96

Dev-dev plot for gambling frequency, showing the posterior mean deviance for each data-point for the inconsistency model plotted against the NMA model (random study, fixed class effect model).........................97

SUPPLEMENTARY MATERIAL S8: Gambling symptom severity NMA: treatment classes, interventions and numbers of participants 98

SUPPLEMENTARY MATERIAL S9: Gambling symptom severity NMA: sensitivity analyses...100

Gambling symptom severity – base-case and sensitivity analyses: results, all treatments versus no treatment 100

Gambling symptom severity – base-case and sensitivity analyses: Network plots....101
Gambling symptom severity – base-case and sensitivity analyses: Model fit statistics....103
SUPPLEMENTARY MATERIAL S10: Gambling symptom severity NMA: relative effects between all pairs of treatment classes....104
SUPPLEMENTARY MATERIAL S11: Gambling frequency NMA: treatment classes, interventions and numbers of participants....107
SUPPLEMENTARY MATERIAL S12: Gambling frequency NMA: network of treatment classes....108
SUPPLEMENTARY MATERIAL S13: Gambling frequency NMA: results versus no treatment....109
SUPPLEMENTARY MATERIAL S14: Gambling frequency NMA: forest plots versus no treatment....110
SUPPLEMENTARY MATERIAL S15: Gambling frequency NMA: sensitivity analyses....111
Gambling frequency – base-case and sensitivity analyses: network plots and results, all treatments versus no treatment....111
Gambling frequency – base-case and sensitivity analyses: Network plots ..112
Gambling frequency – base-case and sensitivity analyses: Model fit statistics 114
SUPPLEMENTARY MATERIAL S16: Gambling frequency NMA: relative effects between all pairs of treatment classes....115
SUPPLEMENTARY MATERIAL S17: Threshold analysis....117
SUPPLEMENTARY MATERIAL S18: Results of the original NMA, undertaken to inform the development of the NICE guideline on gambling-related harms .119

SUPPLEMENTARY MATERIAL S1: Search strategies

Database searches

Date of last search: 23/03/2026

Note: Study filters to locate Randomized controlled trials and Systematic reviews of Randomized controlled trials were used on the Medline, Embase, Emcare and PsycInfo database searches. The study filters are from Chapter 4 of the Cochrane Handbook for Systematic Reviews of Interventions:

Lefebvre C, Glanville J, Briscoe S, Featherstone R, Littlewood A, Metzendorf M-I, Noel-Storr A, Paynter R, Rader T, Thomas J, Wieland LS. Chapter 4: Searching for and selecting studies [last updated March 2025]. In: Higgins JPT, Thomas J, Chandler J, Cumpston M, Li T, Page MJ, Welch VA (editors). Cochrane Handbook for Systematic Reviews of Interventions version 6.5.1 Cochrane, 2025. Available from *cochrane.org/handbook*.

As well as the Technical supplement to Chapter 4:

Lefebvre C, Glanville J, Briscoe S, Featherstone R, Littlewood A, Metzendorf M-I, Noel-Storr A, Paynter R, Rader T, Thomas J, Wieland LS. Technical Supplement to Chapter 4: Searching for and selecting studies [last updated September 2024]. In: Higgins JPT, Thomas J, Chandler J, Cumpston M, Li T, Page MJ, Welch VA (editors). Cochrane Handbook for Systematic Reviews of Interventions version 6.5. Cochrane, 2024. Available from cochrane.org/handbook.

Database: Applied Social Science Index and Abstracts (ASSIA) [ProQuest]

#	Searches
	AB,TI (gambl* or betting or bet or bets or wager* or “gaming machine*” or “slot machine*” or “fruit machine*” or “poker machine*” or “lottery machine*” or “lotteries machine*” or “gaming terminal*” or “slot terminal*” or “fruit terminal*” or “poker terminal*” or “lottery terminal*” or “lotteries terminal*” or pokies or pokey or puggy or fruities)
AND	AB,TI (psycho* or therap* or psychotherap * or cognitive or behaviour* or behavior* or CBT or aversi* or counsel* or “motivational interview*” or “harm reduction” or Psychodrama or dramatherap* or “eye movement” or EMDR or hypno* or “electric stimulat*” or electrostimulat* or electrotherapy or transcranial* or “brain stimulation” or neuromodulat * or TMS or “cognitive bias modification” or retreat or retreats or “self help” or “self care” or “self manage*” or “self directed” or “self guided” or “web based” or “internet based” or “phone based” or app or apps or hotline* or helpline* or “help line*” or “web support*” or “personali* feedback” or “personali* feed back” or gamif* or psychosocial or “psycho social” or “social skill” or “social skills” or assertiveness or “community support” or “social support” or “support program*” or “support group*” or “peer support” or “SMART recovery” or “relapse prevention” or “prevent* relapse” or “secondary prevention” or “recovery capital” or “mutual aid” or “after care” or aftercare or “followup treatment” or “follow up treatment” or “support therapy” or mindfulness or “self compassion” or mentor* or “systemic* intervention*” or finance* or banking or budget* or “self exclu*” or “voluntary exclu*” or “restrict* access” or Gamban or “support mechanism*” or “support model*” or “post resident*” or postresident* or “post treatment” or posttreatment or “relapse program*” or “recovery college*” or “cognitive restructuring*” or biofeedback or neurofeedback or “autogenic training” or meditate or meditation or “crisis intervention*” or “transaction analysis” or “role play” or “role playing” or “breathing exercise*” or qigong or “tai ji” or “tai chi” or yoga or “therapeutic touch” or “node link mapping”)
AND	Additional limits – Date: From January 01 2000

Database: Cochrane Central Register of Controlled Trials (CENTRAL) and Cochrane Database of Systematic Reviews (CDSR) [Cochrane Library Database]

#	Searches
#1	MeSH descriptor: [Gambling] this term only
#2	gambl*:ti,ab
#3	betting:ti,ab
#4	(bet or bets):ti,ab
#5	wager*:ti,ab
#6	((gaming or gambling or slot or fruit or poker or lottery or lotteries) near/5 (machine* or terminal*)):ti,ab
#7	(pokies or pokey or puggy or fruities):ti,ab
#8	((dice or card or cards or roulette or blackjack or poker or baccarat or crap or craps or keno or casino* or bingo or bookmaker* or “book maker” or bookie* or lottery or lotteries or lotto or “scratch card*” or scratchcard* or raffle or raffles

#	Searches
	or sweepstak * or “amusement arcade*” or slot or slots) near/5 (money or monetization or monetisation or monetary or currency or currencies or cryptocurrency or cryptocurrencies or reward* or win or wins or winning* or loss or losses or lose)):ti,ab
#9	((game or games or gaming or gamer*) near/5 (money or monetization or monetisation or monetary)):ti,ab
#10	(“loot box*” or lootbox*):ti,ab
#11	#1 or #2 or #3 or #4 or #5 or #6 or #7 or #8 or #9 or #10
#12	MeSH descriptor: [Psychotherapy] explode all trees
#13	MeSH descriptor: [Mind-Body Therapies] explode all trees
#14	(psycho* near/5 (intervention* or treat* or therap*)):ti,ab
#15	((cogniti* or behavio*) near/5 (intervention* or treat* or therap* or technique*)):ti,ab
#16	CBT:ti,ab
#17	(aversi* near/3 (therap* or treat* or learn*)):ti,ab
#18	(psychotherap * or psychodynamic* or psychoanal*):ti,ab
#19	MeSH descriptor: [Counseling] explode all trees
#20	counsel*:ti,ab
#21	(motivat * near/3 interview*):ti,ab
#22	MeSH descriptor: [Harm Reduction] this term only
#23	(harm* near/3 (reduc* or minimi*)):ti,ab
#24	MeSH descriptor: [Psychodrama] explode all trees
#25	(psychodrama* or “psycho drama*”):ti,ab
#26	(drama* near/3 therap*):ti,ab
#27	dramatherap*:ti,ab
#28	(addict* near/5 (intervention* or treat* or therap* or rehab*)):ti,ab
#29	(trauma* near/5 (intervention* or treat* or therap* or rehab*)):ti,ab
#30	(eye* near/3 mov* near/5 (desensiti* or de-sensiti* or process* or reprocess* or therap* or program* or reprogram*)):ti,ab
#31	EMDR:ti,ab
#32	hypno*:ti,ab
#33	MeSH descriptor: [Electric Stimulation] this term only
#34	MeSH descriptor: [Electric Stimulation Therapy] this term only
#35	MeSH descriptor: [Transcranial Magnetic Stimulation] this term only
#36	MeSH descriptor: [Transcranial Direct Current Stimulation] this term only
#37	MeSH descriptor: [Deep Brain Stimulation] this term only
#38	(stimulat* near/5 (intervention* or treat* or therap* or rehab*)):ti,ab
#39	((neurolo* or brain* or transcranial*) near/3 stimulat*):ti,ab
#40	neuromodulat *:ti,ab
#41	TMS:ti,ab
#42	(cognit* near/3 bias* near/3 modif*):ti,ab
#43	((resident* or inpatient) near/5 (intervention* or treat* or therap* or rehab*)):ti,ab
#44	(retreat or retreats):ti,ab
#45	MeSH descriptor: [Self-Management] this term only
#46	MeSH descriptor: [Self Care] this term only
#47	MeSH descriptor: [Self-Help Groups] this term only
#48	(self near/5 (help* or care or manag* or direct* or guid*)):ti,ab
#49	MeSH descriptor: [Internet-Based Intervention] this term only
#50	((digital* or computer* or online or web or internet or tele* or mobile or phone* or app or apps) near/5 intervention*):ti,ab
#51	MeSH descriptor: [Hotlines] this term only
#52	(hotline* or helpline* or “help line*”):ti,ab
#53	(web near/3 (service* or support*)):ti,ab
#54	(personali* near/3 (feedback or “feed back” or intervention*)):ti,ab
#55	gamif*:ti,ab
#56	((psychosocial* or “psycho social*”) near/5 (intervention* or treat* or therap* or rehab*)):ti,ab
#57	MeSH descriptor: [Social Skills] this term only
#58	MeSH descriptor: [Assertiveness] this term only
#59	((life or social*) near/3 skill* near/5 (intervention* or treat* or therap* or rehab* or train*)):ti,ab
#60	((assertive* or function* or communicat*) near/5 (intervention* or treat* or therap* or rehab* or train*)):ti,ab
#61	((parent or parents or parental or mother or mothers or father or fathers or son or sons or daughter* or sibling* or brother* or sister* or grandparent* or grandfather* or grandmother* or family or families or relatives or cousin* or uncle* or aunt or aunts or auntie* or caregiver* or carer* or friend* or spouse* or husband* or wife or wives or couple or couples or partner or partners or boyfriend* or girlfriend*) near/5 (intervention* or treat* or therap* or rehab* or train* or model*)):ti,ab
#62	((affected or significant) near/3 other* near/5 (intervention* or treat* or therap* or rehab* or train* or model*)):ti,ab
#63	(“loved one*” near/5 (intervention* or treat* or therap* or rehab* or train* or model*)):ti,ab
#64	MeSH descriptor: [Community Support] this term only
#65	MeSH descriptor: [Social Support] this term only
#66	((communit* or neighbor* or neighbour* or religious* or social* or cultur* or ethnic*) near/5 support*):ti,ab
#67	(support* near/5 (organization* or organisation* or program* or group*)):ti,ab
#68	(peer* near/3 (support* or intervention*)):ti,ab
#69	(SMART near/3 recover*):ti,ab
#70	MeSH descriptor: [Secondary Prevention] this term only
#71	((prevent* or avoid*) near/5 (relaps* or recur* or dropout or “drop* out” or second*)):ti,ab

#	Searches
#72	(recover* near/3 capital*):ti,ab
#73	(mutual* near/3 aid*):ti,ab
#74	MeSH descriptor: [Aftercare] this term only
#75	((after* or followup or “follow* up”) near/3 care):ti,ab
#76	aftercare:ti,ab
#77	((followup or “follow* up”) near/3 treat*):ti,ab
#78	(support* near/5 therap*):ti,ab
#79	MeSH descriptor: [Mindfulness] this term only
#80	mindful*:ti,ab
#81	(self near/3 (compass* or forgiv*)):ti,ab
#82	MeSH descriptor: [Mentoring] this term only
#83	MeSH descriptor: [Mentors] this term only
#84	mentor*:ti,ab
#85	((marital* or marriage*) near/5 therap*):ti,ab
#86	(systemic* near/5 (intervention* or treat* or therap* or rehab* or model*)):ti,ab
#87	MeSH descriptor: [Banking, Personal] this term only
#88	MeSH descriptor: [Budgets] this term only
#89	((financ * or bank* or money or spend* or cash or budget*) near/5 (intervention* or manag* or plan* or train* or educat* or limit* or restrict*)):ti,ab
#90	((self or volunt*) near/5 exclu*):ti,ab
#91	(access* near/3 restrict*):ti,ab
#92	Gamban:ti,ab
#93	((financ * or bank* or money or spend* or cash or budget*) near/5 (digital* or computer* or online or web or internet or tele* or mobile or phone* or app or apps)):ti,ab
#94	(support* near/5 (react* or mechanism* or intervention* or model*)):ti,ab
#95	(post near/5 (resident* or treat* or intervention* or therap* or rehab*)):ti,ab
#96	(postresident* or posttreat* or postintervention* or posttherap * or postrehab*):ti,ab
#97	(relaps* near/5 (program* or educat* or train* or learn* or teach* or volunteer* or occupation* or work* or job* or employ* or intervention*)):ti,ab
#98	(recover* near/3 college*):ti,ab
#99	((animal* or equine or art or anger or acceptance or commitment* or implosive or “virtual reality” or relaxation or dance or emotion* or gestalt or horticultur* or mentalisation or mentalization or music* or narrativ * or play or reality or schema or socioenvironmental or “socio environmental” or milieu or “mind body” or laugh*) near/3 therap*):ti,ab
#100	(chronotherapy* or bibliotherapy* or logotherap*):ti,ab
#101	(cognitive near/3 (restructur* or remediat* or refram *)):ti,ab
#102	(feedback near/3 (psycholog* or sensory or neuro*)):ti,ab
#103	(biofeedback or neurofeedback):ti,ab
#104	((autogenic or sensitivity or desensitisation or desensitization or sensitisation or sensitization) near/3 train*):ti,ab
#105	meditat *:ti,ab
#106	((crisis or crises) near/3 intervention*):ti,ab
#107	(transaction* near/3 analys*):ti,ab
#108	“role play*”:ti,ab
#109	(breath* near/3 (exercis * or therap*)):ti,ab
#110	(qigong or “tai ji” or “tai chi” or yoga):ti,ab
#111	(mental* near/3 (heal or heals or healing)):ti,ab
#112	(therap* near/3 touch*):ti,ab
#113	(node* near/3 link* near/3 map*):ti,ab
#114	#12 or #13 or #14 or #15 or #16 or #17 or #18 or #19 or #20 or #21 or #22 or #23 or #24 or #25 or #26 or #27 or #28 or #29 or #30 or #31 or #32 or #33 or #34 or #35 or #36 or #37 or #38 or #39 or #40 or #41 or #42 or #43 or #44 or #45 or #46 or #47 or #48 or #49 or #50 or #51 or #52 or #53 or #54 or #55 or #56 or #57 or #58 or #59 or #60 or #61 or #62 or #63 or #64 or #65 or #66 or #67 or #68 or #69 or #70 or #71 or #72 or #73 or #74 or #75 or #76 or #77 or #78 or #79 or #80 or #81 or #82 or #83 or #84 or #85 or #86 or #87 or #88 or #89 or #90 or #91 or #92 or #93 or #94 or #95 or #96 or #97 or #98 or #99 or #100 or #101 or #102 or #103 or #104 or #105 or #106 or #107 or #108 or #109 or #110 or #111 or #112 or #113
#115	#11 and #114
#116	#11 and #114 with Cochrane Library publication date Between Jan 2000 and Jun 2022

Database: Cumulative Index to Nursing and Allied Health Literature (CINAHL) [EBSCO]

#	Searches
S 1	TI (gambl* or betting or bet or bets or wager* or “gaming machine*” or “slot machine*” or “fruit machine*” or “poker machine*” or “lottery machine*” or “lotteries machine*” or “gaming terminal*” or “slot terminal*” or “fruit terminal*” or “poker terminal*” or “lottery terminal*” or “lotteries terminal*” or pokies or pokey or puggy or fruities) Limiters – Published Date: 20000101-20221231
S 2	TI (psycho* or therap* or psychotherap * or cognitive or behaviour* or behavior* or CBT or aversi* or counsel* or “motivational interview*” or “harm reduction” or Psychodrama or dramatherap* or “eye movement” or EMDR or hypno* or “electric stimulat*” or electrostimulat* or electrotherapy or transcranial* or “brain stimulation” or neuromodulat * or TMS or “cognitive bias modification” or retreat or retreats or “self help” or “self care” or “self manage*” or “self directed” or “self guided” or “web based” or “internet based” or “phone based” or app or apps or hotline* or helpline* or “help line*” or “web support*” or

#	Searches
	"personali* feedback" or "personali* feed back" or gamif* or psychosocial or "psycho social" or "social skill" or "social skills" or assertiveness or "community support" or "social support" or "support program*" or "support group*" or "peer support" or "SMART recovery" or "relapse prevention" or "prevent* relapse" or "secondary prevention" or "recovery capital" or "mutual aid" or "after care" or aftercare or "followup treatment" or "follow up treatment" or "support therapy" or mindfulness or "self compassion" or mentor* or "systemic* intervention*" or finance* or banking or budget* or "self exclu*" or "voluntary exclu*" or "restrict* access" or Gamban or "support mechanism*" or "support model*" or "post resident*" or postresident* or "post treatment" or posttreatment or "relapse program*" or "recovery college*" or "cognitive restructuring*" or biofeedback or neurofeedback or "autogenic training" or meditate or meditation or "crisis intervention*" or "transaction analysis" or "role play" or "role playing" or "breathing exercise*" or qigong or "tai ji" or "tai chi" or yoga or "therapeutic touch" or "node link mapping") Limiters – Published Date: 20000101-20221231
S3	S1 and S2

Database: Embase [Ovid]

#	Searches
1	GAMBLING/
2	PATHOLOGICAL GAMBLING/
3	gambl*.ti,ab.
4	betting.ti,ab.
5	(bet or bets).ti,ab.
6	wager*.ti,ab.
7	((gaming or gambling or slot or fruit or poker or lottery or lotteries) adj5 (machine? Or terminal?)).ti,ab.
8	(pokies or pokey or puggy or fruities).ti,ab.
9	((dice or card? Or roulette or blackjack or poker or baccarat or crap or craps or keno or casino? Or bingo or bookmaker? Or book maker or bookie? Or lottery or lotteries or lotto or scratch card? Or scratchcard? Or raffle or raffles or sweepstak * or amusement arcade? Or slot?) adj5 (money or monetization or monetisation or monetary or currency or currencies or cryptocurrency or cryptocurrencies or reward* or win or wins or winning* or loss or losses or lose)).ti,ab.
10	((game or games or gaming or gamer?) adj5 (money or monetization or monetisation or monetary)).ti,ab.
11	(loot box* or lootbox*).ti,ab.
12	or/1-11
13	exp PSYCHOTHERAPY/
14	exp ALTERNATIVE MEDICINE/
15	(psycho* adj5 (intervention? Or treat* or therap*)).ti,ab.
16	((cogniti* or behavio*) adj5 (intervention? Or treat* or therap* or technique?)).ti,ab.
17	CBT.ti,ab.
18	(aversi* adj3 (therap* or treat* or learn*)).ti,ab.
19	(psychotherap * or psychodynamic? Or psychoanal*).ti,ab.
20	exp COUNSELING/
21	counsel*.ti,ab.
22	(motivat * adj3 interview*).ti,ab.
23	HARM REDUCTION/
24	(harm* adj3 (reduc* or minimi*)).ti,ab.
25	(psychodrama* or psycho drama*).ti,ab.
26	(drama* adj3 therap*).ti,ab.
27	dramatherap*.ti,ab.
28	(addict* adj5 (intervention? Or treat* or therap* or rehab*)).ti,ab.
29	(trauma* adj5 (intervention? Or treat* or therap* or rehab*)).ti,ab.
30	(eye? Adj3 mov* adj5 (desensiti* or de-sensiti* or process* or reprocess* or therap* or program* or reprogram*)).ti,ab.
31	EMDR.ti,ab.
32	hypno*.ti,ab.
33	ELECTROSTIMULATION/
34	ELECTROTHERAPY/
35	exp TRANSCRANIAL MAGNETIC STIMULATION/
36	TRANSCRANIAL DIRECT CURRENT STIMULATION/
37	BRAIN DEPTH STIMULATION/
38	(stimulat* adj5 (intervention? Or treat* or therap* or rehab*)).ti,ab.
39	((neurolo* or brain? Or transcranial*) adj3 stimulat*).ti,ab.
40	neuromodulat *.ti,ab.
41	TMS.ti,ab.
42	(cognit* adj3 bias* adj3 modif*).ti,ab.
43	((resident* or inpatient) adj5 (intervention? Or treat* or therap* or rehab*)).ti,ab.
44	retreat?.ti,ab.
45	SELF CARE/
46	SELF HELP/
47	(self adj5 (help* or care or manag* or direct* or guid*)).ti,ab.
48	WEB-BASED INTERVENTION/
49	((digital* or computer* or online or web or internet or tele* or mobile or phone? Or app?) adj5 intervention?).ti,ab.
50	HOTLINE/
51	(hotline? Or helpline? Or help line?).ti,ab.

#	Searches
52	(web adj3 (service? Or support*)).ti,ab.
53	(personali* adj3 (feedback or feed back or intervention?)).ti,ab.
54	gamif*.ti,ab.
55	PSYCHOSOCIAL CARE/
56	((psychosocial* or psycho social*) adj5 (intervention? Or treat* or therap* or rehab*)).ti,ab.
57	SOCIAL COMPETENCE/
58	ASSERTIVENESS/
59	((life or social*) adj3 skill? Adj5 (intervention? Or treat* or therap* or rehab* or train*)).ti,ab.
60	((assertive* or function* or communicat*) adj5 (intervention? Or treat* or therap* or rehab* or train*)).ti,ab.
61	((parent? Or parental or mother? Or father? Or son? Or daughter? Or sibling? Or brother? Or sister? Or grandparent? Or grandfather? Or grandmother? Or family or families or relatives or cousin? Or uncle? Or aunt? Or auntie? Or caregiver? Or carer? Or friend? Or spouse? Or husband? Or wife or wives or couple or couples or partner or partners or boyfriend? Or girlfriend?) adj5 (intervention? Or treat* or therap* or rehab* or train* or model?)).ti,ab.
62	((affected or significant) adj3 other? Adj5 (intervention? Or treat* or therap* or rehab* or train* or model?)).ti,ab.
63	(loved one* adj5 (intervention? Or treat* or therap* or rehab* or train* or model?)).ti,ab.
64	SOCIAL SUPPORT/
65	((communit* or neighbo?r* or religious* or social* or cultur* or ethnic*) adj5 support*).ti,ab.
66	exp SUPPORT GROUP/
67	(support* adj5 (organization? Or organisation? Or program* or group?)).ti,ab.
68	(peer? Adj3 (support* or intervention*)).ti,ab.
69	(SMART adj3 recover*).ti,ab.
70	SECONDARY PREVENTION/
71	((prevent* or avoid*) adj5 (relaps* or recur* or dropout or drop* out or second*)).ti,ab.
72	(recover* adj3 capital*).ti,ab.
73	(mutual* adj3 aid*).ti,ab.
74	AFTERCARE/
75	((after* or followup or follow* up) adj3 care).ti,ab.
76	aftercare.ti,ab.
77	((followup or follow* up) adj3 treat*).ti,ab.
78	(support* adj5 therap*).ti,ab.
79	mindful*.ti,ab.
80	(self adj3 (compass* or forgiv*)).ti,ab.
81	MENTORING/
82	MENTOR/
83	mentor*.ti,ab.
84	((marital* or marriage?) adj5 therap*).ti,ab.
85	(systemic* adj5 (intervention? Or treat* or therap* or rehab* or model?)).ti,ab.
86	BANK ACCOUNT/
87	BUDGET/
88	((financ * or bank* or money or spend* or cash or budget*) adj5 (intervention? Or manag* or plan* or train* or educat* or limit* or restrict*)).ti,ab.
89	((self or volunt*) adj5 exclu*).ti,ab.
90	(access* adj3 restrict*).ti,ab.
91	Gamban.ti,ab.
92	((financ * or bank* or money or spend* or cash or budget*) adj5 (digital* or computer* or online or web or internet or tele* or mobile or phone? Or app?)).ti,ab.
93	(support* adj5 (react* or mechanism? Or intervention? Or model*)).ti,ab.
94	(post adj5 (resident* or treat* or intervention? Or therap* or rehab*)).ti,ab.
95	(postresident* or posttreat* or postintervention? Or posttherap * or postrehab*).ti,ab.
96	(relaps* adj5 (program* or educat* or train* or learn* or teach* or volunteer* or occupation* or work* or job? Or employ* or intervention?)).ti,ab.
97	(recover* adj3 college?).ti,ab.
98	((animal? Or equine or art or anger or acceptance or commitment? Or implosive or virtual reality or relaxation or dance or emotion* or gestalt or horticultur* or mentali?ation or music* or narrativ * or play or reality or schema or socioenvironmental or socio environmental or milieu or mind body or laugh*) adj3 therap*).ti,ab.
99	(chronotherapy* or bibliotherapy* or logotherap*).ti,ab.
100	(cognitive adj3 (restructur* or remediat* or refram *)).ti,ab.
101	(feedback adj3 (psycholog* or sensory or neuro*)).ti,ab.
102	(biofeedback or neurofeedback).ti,ab.
103	((autogenic or sensitivity or desensiti?ation or sensiti?ation) adj3 train*).ti,ab.
104	exp MEDITATION/
105	meditat *.ti,ab.
106	CRISIS INTERVENTION/
107	(cris?s adj3 intervention?).ti,ab.
108	(transaction* adj3 analys*).ti,ab.
109	role play*.ti,ab.
110	exp BREATHING EXERCISE/
111	(breath* adj3 (exercis * or therap*)).ti,ab.
112	TAI CHI/

#	Searches
113	exp YOGA/
114	(qigong or tai ji or tai chi or yoga).ti,ab.
115	(mental* adj3 (heal? Or healing)).ti,ab.
116	(therap* adj3 touch*).ti,ab.
117	(node? Adj3 link* adj3 map*).ti,ab.
118	or/13-117
119	12 and 118
120	limit 119 to english language
121	limit 120 to yr="2000 -Current"
122	letter.pt. or LETTER/
123	note.pt.
124	editorial.pt.
125	CASE REPORT/ or CASE STUDY/
126	(letter or comment*).ti.
127	or/122-126
128	RANDOMIZED CONTROLLED TRIAL/ or random*.ti,ab.
129	127 not 128
130	ANIMAL/ not HUMAN/
131	NONHUMAN/
132	exp ANIMAL EXPERIMENT/
133	exp EXPERIMENTAL ANIMAL/
134	ANIMAL MODEL/
135	exp RODENT/
136	(rat or rats or mouse or mice).ti.
137	or/129-136
138	121 not 137
139	SYSTEMATIC REVIEW/
140	META-ANALYSIS/
141	(meta analy* or metanaly* or metaanaly*).ti,ab.
142	((systematic or evidence) adj2 (review* or overview*)).ti,ab.
143	(reference list* or bibliograph* or hand search* or manual search* or relevant journals).ab.
144	(search strategy or search criteria or systematic search or study selection or data extraction).ab.
145	(search* adj4 literature).ab.
146	(medline or pubmed or cochrane or embase or psychlit or psyclit or psychinfo or psycinfo or cinahl or science citation index or bids or cancerlit).ab.
147	((pool* or combined) adj2 (data or trials or studies or results)).ab.
148	cochrane.jw.
149	or/139-148
150	random*.ti,ab.
151	factorial*.ti,ab.
152	(crossover* or cross over*).ti,ab.
153	((doubl* or singl*) adj blind*).ti,ab.
154	(assign* or allocat* or volunteer* or placebo*).ti,ab.
155	CROSSOVER PROCEDURE/
156	SINGLE BLIND PROCEDURE/
157	RANDOMIZED CONTROLLED TRIAL/
158	DOUBLE BLIND PROCEDURE/
159	or/150-158
160	EPIDEMIOLOGY/ or CONTROLLED STUDY/ or exp CASE CONTROL STUDY/ or PROSPECTIVE STUDY/ or RETROSPECTIVE STUDY/ or COHORT ANALYSIS/ or FOLLOW UP/ or CROSS-SECTIONAL STUDY/ or exp CLINICAL TRIAL/ or COMPARATIVE STUDY/
161	(control and study).mp.
162	program.mp.
163	or/160-162
164	(ANIMAL/ not HUMAN/) or EDITORIAL/ or REVIEW/ or META-ANALYSIS/ or CONSENSUS/ or PRACTICE GUIDELINE/
165	hi.fs. or case report.mp.
166	or/164-165
167	163 not 166
168	138 and 149
169	138 and 159
170	138 and 167
171	or/168-170

Database: Emcare [Ovid]

#	Searches
1	GAMBLING/
2	PATHOLOGICAL GAMBLING/
3	gambl*.ti,ab.

#	Searches
4	betting.ti,ab.
5	(bet or bets).ti,ab.
6	wager*.ti,ab.
7	((gaming or gambling or slot or fruit or poker or lottery or lotteries) adj5 (machine? Or terminal?)).ti,ab.
8	(pokies or pokey or puggy or fruities).ti,ab.
9	((dice or card? Or roulette or blackjack or poker or baccarat or crap or craps or keno or casino? Or bingo or bookmaker? Or book maker or bookie? Or lottery or lotteries or lotto or scratch card? Or scratchcard? Or raffle or raffles or sweepstak * or amusement arcade? Or slot?) adj5 (money or monetization or monetisation or monetary or currency or currencies or cryptocurrency or cryptocurrencies or reward* or win or wins or winning* or loss or losses or lose)).ti,ab.
10	((game or games or gaming or gamer?) adj5 (money or monetization or monetisation or monetary)).ti,ab.
11	(loot box* or lootbox*).ti,ab.
12	or/1-11
13	exp PSYCHOTHERAPY/
14	exp ALTERNATIVE MEDICINE/
15	(psycho* adj5 (intervention? Or treat* or therap*)).ti,ab.
16	((cogniti* or behavio*) adj5 (intervention? Or treat* or therap* or technique?)).ti,ab.
17	CBT.ti,ab.
18	(aversi* adj3 (therap* or treat* or learn*)).ti,ab.
19	(psychotherap * or psychodynamic? Or psychoanal*).ti,ab.
20	exp COUNSELING/
21	counsel*.ti,ab.
22	(motivat * adj3 interview*).ti,ab.
23	HARM REDUCTION/
24	(harm* adj3 (reduc* or minimi*)).ti,ab.
25	(psychodrama* or psycho drama*).ti,ab.
26	(drama* adj3 therap*).ti,ab.
27	dramatherap*.ti,ab.
28	(addict* adj5 (intervention? Or treat* or therap* or rehab*)).ti,ab.
29	(trauma* adj5 (intervention? Or treat* or therap* or rehab*)).ti,ab.
30	(eye? Adj3 mov* adj5 (desensiti* or de-sensiti* or process* or reprocess* or therap* or program* or reprogram*)).ti,ab.
31	EMDR.ti,ab.
32	hypno*.ti,ab.
33	ELECTROSTIMULATION/
34	ELECTROTHERAPY/
35	exp TRANSCRANIAL MAGNETIC STIMULATION/
36	TRANSCRANIAL DIRECT CURRENT STIMULATION/
37	BRAIN DEPTH STIMULATION/
38	(stimulat* adj5 (intervention? Or treat* or therap* or rehab*)).ti,ab.
39	((neurolo* or brain? Or transcranial*) adj3 stimulat*).ti,ab.
40	neuromodulat *.ti,ab.
41	TMS.ti,ab.
42	(cognit* adj3 bias* adj3 modif*).ti,ab.
43	((resident* or inpatient) adj5 (intervention? Or treat* or therap* or rehab*)).ti,ab.
44	retreat?.ti,ab.
45	SELF CARE/
46	SELF HELP/
47	(self adj5 (help* or care or manag* or direct* or guid*)).ti,ab.
48	WEB-BASED INTERVENTION/
49	((digital* or computer* or online or web or internet or tele* or mobile or phone? Or app?) adj5 intervention?).ti,ab.
50	HOTLINE/
51	(hotline? Or helpline? Or help line?).ti,ab.
52	(web adj3 (service? Or support*)).ti,ab.
53	(personali* adj3 (feedback or feed back or intervention?)).ti,ab.
54	gamif*.ti,ab.
55	PSYCHOSOCIAL CARE/
56	((psychosocial* or psycho social*) adj5 (intervention? Or treat* or therap* or rehab*)).ti,ab.
57	SOCIAL COMPETENCE/
58	ASSERTIVENESS/
59	((life or social*) adj3 skill? Adj5 (intervention? Or treat* or therap* or rehab* or train*)).ti,ab.
60	((assertive* or function* or communicat*) adj5 (intervention? Or treat* or therap* or rehab* or train*)).ti,ab.
61	((parent? Or parental or mother? Or father? Or son? Or daughter? Or sibling? Or brother? Or sister? Or grandparent? Or grandfather? Or grandmother? Or family or families or relatives or cousin? Or uncle? Or aunt? Or auntie? Or caregiver? Or carer? Or friend? Or spouse? Or husband? Or wife or wives or couple or couples or partner or partners or boyfriend? Or girlfriend?) adj5 (intervention? Or treat* or therap* or rehab* or train* or model?)).ti,ab.
62	((affected or significant) adj3 other? Adj5 (intervention? Or treat* or therap* or rehab* or train* or model?)).ti,ab.
63	(loved one* adj5 (intervention? Or treat* or therap* or rehab* or train* or model?)).ti,ab.
64	SOCIAL SUPPORT/
65	((communit* or neighbo?r* or religious* or social* or cultur* or ethnic*) adj5 support*).ti,ab.
66	exp SUPPORT GROUP/

#	Searches
67	(support* adj5 (organization? Or organisation? Or program* or group?)).ti,ab.
68	(peer? Adj3 (support* or intervention*)).ti,ab.
69	(SMART adj3 recover*).ti,ab.
70	SECONDARY PREVENTION/
71	((prevent* or avoid*) adj5 (relaps* or recur* or dropout or drop* out or second*)).ti,ab.
72	(recover* adj3 capital*).ti,ab.
73	(mutual* adj3 aid*).ti,ab.
74	AFTERCARE/
75	((after* or followup or follow* up) adj3 care).ti,ab.
76	aftercare.ti,ab.
77	((followup or follow* up) adj3 treat*).ti,ab.
78	(support* adj5 therap*).ti,ab.
79	mindful*.ti,ab.
80	(self adj3 (compass* or forgiv*)).ti,ab.
81	MENTORING/
82	MENTOR/
83	mentor*.ti,ab.
84	((marital* or marriage?) adj5 therap*).ti,ab.
85	(systemic* adj5 (intervention? Or treat* or therap* or rehab* or model?)).ti,ab.
86	BANK ACCOUNT/
87	BUDGET/
88	((financ * or bank* or money or spend* or cash or budget*) adj5 (intervention? Or manag* or plan* or train* or educat* or limit* or restrict*)).ti,ab.
89	((self or volunt*) adj5 exclu*).ti,ab.
90	(access* adj3 restrict*).ti,ab.
91	Gamban.ti,ab.
92	((financ * or bank* or money or spend* or cash or budget*) adj5 (digital* or computer* or online or web or internet or tele* or mobile or phone? Or app?)).ti,ab.
93	(support* adj5 (react* or mechanism? Or intervention? Or model*)).ti,ab.
94	(post adj5 (resident* or treat* or intervention? Or therap* or rehab*)).ti,ab.
95	(postresident* or posttreat* or postintervention? Or posttherap * or postrehab*).ti,ab.
96	(relaps* adj5 (program* or educat* or train* or learn* or teach* or volunteer* or occupation* or work* or job? Or employ* or intervention?)).ti,ab.
97	(recover* adj3 college?).ti,ab.
98	((animal? Or equine or art or anger or acceptance or commitment? Or implosive or virtual reality or relaxation or dance or emotion* or gestalt or horticultur* or mentali?ation or music* or narrativ * or play or reality or schema or socioenvironmental or socio environmental or milieu or mind body or laugh*) adj3 therap*).ti,ab.
99	(chronotherapy* or bibliotherapy* or logotherap*).ti,ab.
100	(cognitive adj3 (restructur* or remediat* or refram *)).ti,ab.
101	(feedback adj3 (psycholog* or sensory or neuro*)).ti,ab.
102	(biofeedback or neurofeedback).ti,ab.
103	((autogenic or sensitivity or desensiti?ation or sensiti?ation) adj3 train*).ti,ab.
104	exp MEDITATION/
105	meditat *.ti,ab.
106	CRISIS INTERVENTION/
107	(cris?s adj3 intervention?).ti,ab.
108	(transaction* adj3 analys*).ti,ab.
109	role play*.ti,ab.
110	exp BREATHING EXERCISE/
111	(breath* adj3 (exercis * or therap*)).ti,ab.
112	TAI CHI/
113	exp YOGA/
114	(qigong or tai ji or tai chi or yoga).ti,ab.
115	(mental* adj3 (heal? Or healing)).ti,ab.
116	(therap* adj3 touch*).ti,ab.
117	(node? Adj3 link* adj3 map*).ti,ab.
118	or/13-117
119	12 and 118
120	limit 119 to english language
121	limit 120 to yr="2000 -Current"
122	letter.pt. or LETTER/
123	note.pt.
124	editorial.pt.
125	CASE REPORT/ or CASE STUDY/
126	(letter or comment*).ti.
127	or/122-126
128	RANDOMIZED CONTROLLED TRIAL/ or random*.ti,ab.
129	127 not 128
130	ANIMAL/ not HUMAN/

#	Searches
131	NONHUMAN/
132	exp ANIMAL EXPERIMENT/
133	exp EXPERIMENTAL ANIMAL/
134	ANIMAL MODEL/
135	exp RODENT/
136	(rat or rats or mouse or mice).ti.
137	or/129-136
138	121 not 137
139	SYSTEMATIC REVIEW/
140	META-ANALYSIS/
141	(meta analy* or metanaly* or metaanaly*).ti,ab.
142	((systematic or evidence) adj2 (review* or overview*)).ti,ab.
143	(reference list* or bibliograph* or hand search* or manual search* or relevant journals).ab.
144	(search strategy or search criteria or systematic search or study selection or data extraction).ab.
145	(search* adj4 literature).ab.
146	(medline or pubmed or cochrane or embase or psychlit or psyclit or psychinfo or psycinfo or cinahl or science citation index or bids or cancerlit).ab.
147	((pool* or combined) adj2 (data or trials or studies or results)).ab.
148	cochrane.jw.
149	or/139-148
150	random*.ti,ab.
151	factorial*.ti,ab.
152	(crossover* or cross over*).ti,ab.
153	((doubl* or singl*) adj blind*).ti,ab.
154	(assign* or allocat* or volunteer* or placebo*).ti,ab.
155	CROSSOVER PROCEDURE/
156	SINGLE BLIND PROCEDURE/
157	RANDOMIZED CONTROLLED TRIAL/
158	DOUBLE BLIND PROCEDURE/
159	or/150-158
160	EPIDEMIOLOGY/ or CONTROLLED STUDY/ or exp CASE CONTROL STUDY/ or PROSPECTIVE STUDY/ or RETROSPECTIVE STUDY/ or COHORT ANALYSIS/ or FOLLOW UP/ or CROSS-SECTIONAL STUDY/ or exp CLINICAL TRIAL/ or COMPARATIVE STUDY/
161	(control and study).mp.
162	program.mp.
163	or/160-162
164	(ANIMAL/ not HUMAN/) or EDITORIAL/ or REVIEW/ or META-ANALYSIS/ or CONSENSUS/ or PRACTICE GUIDELINE/
165	[hi.fs. or case report.mp.]
166	or/164-165
167	163 not 166
168	138 and 149
169	138 and 159
170	138 and 167
171	or/168-170

Database: Epistemonikos

#	Searches
	title:((gambl* OR betting OR bet OR bets OR wager* OR “gaming machine*” OR “slot machine*” OR “fruit machine*” OR “poker machine*” OR “lottery machine*” OR “lotteries machine*” OR “gaming terminal*” OR “slot terminal*” OR “fruit terminal*” OR “poker terminal*” OR “lottery terminal*” OR “lotteries terminal*” OR pokies OR pokey OR puggy OR fruities) AND (psycho* or therap* or psychotherap * or cognitive or behaviour* or behavior* or CBT or aversi* or counsel* or “motivational interview*” or “harm reduction” or Psychodrama or dramatherap* or “eye movement” or EMDR or hypno* or “electric stimulat*” or electrostimulat* or electrotherapy or transcranial* or “brain stimulation” or neuromodulat * or TMS or “cognitive bias modification” or retreat or retreats or “self help” or “self care” or “self manage*” or “self directed” or “self guided” or “web based” or “internet based” or “phone based” or app or apps or hotline* or helpline* or “help line*” or “web support*” or “personali* feedback” or “personali* feed back” or gamif* or psychosocial or “psycho social” or “social skill” or “social skills” or assertiveness or “community support” or “social support” or “support program*” or “support group*” or “peer support” or “SMART recovery” or “relapse prevention” or “prevent* relapse” or “secondary prevention” or “recovery capital” or “mutual aid” or “after care” or aftercare or “followup treatment” or “follow up treatment” or “support therapy” or mindfulness or “self compassion” or mentor* or “systemic* intervention*” or finance* or banking or budget* or “self exclu*” or “voluntary exclu*” or “restrict* access” or Gamban or “support mechanism*” or “support model*” or “post resident*” or postresident* or “post treatment” or posttreatment or “relapse program*” or “recovery college*” or “cognitive restructuring*” or biofeedback or neurofeedback or “autogenic training” or meditate or meditation or “crisis intervention*” or “transaction analysis” or “role play” or “role playing” or “breathing exercise*” or qigong or “tai ji” or “tai chi” or yoga or “therapeutic touch” or “node link mapping”))<br>Publication year: 2000-2022

Database: Health Information Management Consortium (HMIC) [Ovid]

#	Searches
1	GAMBLING/
2	GAMBLERS/
3	GAMBLING MACHINES/
4	AMUSEMENT ARCADES/
5	CASINOS/
6	BOOKMAKERS/
7	LOTTERIES/
8	NATIONAL LOTTERY/
9	gambl*.ti,ab.
10	betting.ti,ab.
11	(bet or bets).ti,ab.
12	wager*.ti,ab.
13	((gaming or gambling or slot or fruit or poker or lottery or lotteries) adj5 (machine? Or terminal?)).ti,ab.
14	(pokies or pokey or puggy or fruities).ti,ab.
15	((dice or card? Or roulette or blackjack or poker or baccarat or crap or craps or keno or casino? Or bingo or bookmaker? Or book maker or bookie? Or lottery or lotteries or lotto or scratch card? Or scratchcard? Or raffle or raffles or sweepstak * or amusement arcade? Or slot?) adj5 (money or monetization or monetisation or monetary or currency or currencies or cryptocurrency or cryptocurrencies or reward* or win or wins or winning* or loss or losses or lose)).ti,ab.
16	((game or games or gaming or gamer?) adj5 (money or monetization or monetisation or monetary)).ti,ab.
17	(loot box* or lootbox*).ti,ab.
18	or/1-17
19	exp PSYCHOTHERAPY/
20	exp COMPLEMENTARY MEDICINE/
21	(psycho* adj5 (intervention? Or treat* or therap*)).ti,ab.
22	((cogniti* or behavio*) adj5 (intervention? Or treat* or therap* or technique?)).ti,ab.
23	CBT.ti,ab.
24	(aversi* adj3 (therap* or treat* or learn*)).ti,ab.
25	(psychotherap * or psychodynamic? Or psychoanal*).ti,ab.
26	exp COUNSELLING/
27	COUNSELLING SERVICES/
28	counsel*.ti,ab.
29	(motivat * adj3 interview*).ti,ab.
30	HARM REDUCTION/
31	(harm* adj3 (reduc* or minimi*)).ti,ab.
32	(psychodrama* or psycho drama*).ti,ab.
33	(drama* adj3 therap*).ti,ab.
34	dramatherap*.ti,ab.
35	(addict* adj5 (intervention? Or treat* or therap* or rehab*)).ti,ab.
36	(trauma* adj5 (intervention? Or treat* or therap* or rehab*)).ti,ab.
37	(eye? Adj3 mov* adj5 (desensiti* or de-sensiti* or process* or reprocess* or therap* or program* or reprogram*)).ti,ab.
38	EMDR.ti,ab.
39	HYPNOSIS/
40	hypno*.ti,ab.
41	ELECTROTHERAPY/
42	(stimulat* adj5 (intervention? Or treat* or therap* or rehab*)).ti,ab.
43	((neurolo* or brain? Or transcranial*) adj3 stimulat*).ti,ab.
44	neuromodulat *.ti,ab.
45	TMS.ti,ab.
46	(cognit* adj3 bias* adj3 modif*).ti,ab.
47	((resident* or inpatient) adj5 (intervention? Or treat* or therap* or rehab*)).ti,ab.
48	retreat?.ti,ab.
49	SELF MANAGEMENT/
50	exp SELF CARE/
51	SELF HELP/
52	SELF HELP ORGANISATIONS/
53	SELF HELP HEALTH ORGANISATIONS/
54	SELF HELP GROUPS/
55	SELF HELP CLUBS/
56	(self adj5 (help* or care or manag* or direct* or guid*)).ti,ab.
57	((digital* or computer* or online or web or internet or tele* or mobile or phone? Or app?) adj5 intervention?).ti,ab.
58	(hotline? Or helpline? Or help line?).ti,ab.
59	(web adj3 (service? Or support*)).ti,ab.
60	(personali* adj3 (feedback or feed back or intervention?)).ti,ab.
61	gamif*.ti,ab.
62	((psychosocial* or psycho social*) adj5 (intervention? Or treat* or therap* or rehab*)).ti,ab.
63	SOCIAL SKILLS TRAINING/
64	ASSERTIVENESS TRAINING/
65	((life or social*) adj3 skill? Adj5 (intervention? Or treat* or therap* or rehab* or train*)).ti,ab.

#	Searches
66	((assertive* or function* or communicat*) adj5 (intervention? Or treat* or therap* or rehab* or train*)).ti,ab.
67	((parent? Or parental or mother? Or father? Or son? Or daughter? Or sibling? Or brother? Or sister? Or grandparent? Or grandfather? Or grandmother? Or family or families or relatives or cousin? Or uncle? Or aunt? Or auntie? Or caregiver? Or carer? Or friend? Or spouse? Or husband? Or wife or wives or couple or couples or partner or partners or boyfriend? Or girlfriend?) adj5 (intervention? Or treat* or therap* or rehab* or train* or model?)).ti,ab.
68	((affected or significant) adj3 other? Adj5 (intervention? Or treat* or therap* or rehab* or train* or model?)).ti,ab.
69	(loved one* adj5 (intervention? Or treat* or therap* or rehab* or train* or model?)).ti,ab.
70	SOCIAL SUPPORT/
71	SUPPORT GROUPS/
72	((communit* or neighbo?r* or religious* or social* or cultur* or ethnic*) adj5 support*).ti,ab.
73	(support* adj5 (organization? Or organisation? Or program* or group?)).ti,ab.
74	(peer? Adj3 (support* or intervention*)).ti,ab.
75	(SMART adj3 recover*).ti,ab.
76	((prevent* or avoid*) adj5 (relaps* or recur* or dropout or drop* out or second*)).ti,ab.
77	(recover* adj3 capital*).ti,ab.
78	(mutual* adj3 aid*).ti,ab.
79	AFTER CARE/
80	MEDICAL AFTER CARE/
81	PSYCHIATRIC AFTER CARE/
82	((after* or followup or follow* up) adj3 care).ti,ab.
83	aftercare.ti,ab.
84	((followup or follow* up) adj3 treat*).ti,ab.
85	(support* adj5 therap*).ti,ab.
86	mindful*.ti,ab.
87	(self adj3 (compass* or forgiv*)).ti,ab.
88	MENTORING/
89	mentor*.ti,ab.
90	((marital* or marriage?) adj5 therap*).ti,ab.
91	(systemic* adj5 (intervention? Or treat* or therap* or rehab* or model?)).ti,ab.
92	PERSONAL FINANCE/
93	BUDGETS/
94	FAMILY BUDGETS/
95	INDIVIDUAL BUDGETS/
96	((financ * or bank* or money or spend* or cash or budget*) adj5 (intervention? Or manag* or plan* or train* or educat* or limit* or restrict*)).ti,ab.
97	((self or volunt*) adj5 exclu*).ti,ab.
98	(access* adj3 restrict*).ti,ab.
99	Gamban.ti,ab.
100	((financ * or bank* or money or spend* or cash or budget*) adj5 (digital* or computer* or online or web or internet or tele* or mobile or phone? Or app?)).ti,ab.
101	(support* adj5 (react* or mechanism? Or intervention? Or model*)).ti,ab.
102	(post adj5 (resident* or treat* or intervention? Or therap* or rehab*)).ti,ab.
103	(postresident* or posttreat* or postintervention? Or posttherap * or postrehab*).ti,ab.
104	(relaps* adj5 (program* or educat* or train* or learn* or teach* or volunteer* or occupation* or work* or job? Or employ* or intervention?)).ti,ab.
105	(recover* adj3 college?).ti,ab.
106	((animal? Or equine or art or anger or acceptance or commitment? Or implosive or virtual reality or relaxation or dance or emotion* or gestalt or horticultur* or mentali?ation or music* or narrativ * or play or reality or schema or socioenvironmental or socio environmental or milieu or mind body or laugh*) adj3 therap*).ti,ab.
107	(chronotherapy* or bibliotherapy* or logotherap*).ti,ab.
108	(cognitive adj3 (restructur* or remediat* or refram *)).ti,ab.
109	(feedback adj3 (psycholog* or sensory or neuro*)).ti,ab.
110	(biofeedback or neurofeedback).ti,ab.
111	((autogenic or sensitivity or desensiti?ation or sensiti?ation) adj3 train*).ti,ab.
112	meditat *.ti,ab.
113	(cris?s adj3 intervention?).ti,ab.
114	(transaction* adj3 analys*).ti,ab.
115	role play*.ti,ab.
116	BREATHING EXERCISES/
117	(breath* adj3 (exercis * or therap*)).ti,ab.
118	(qigong or tai ji or tai chi or yoga).ti,ab.
119	(mental* adj3 (heal? Or healing)).ti,ab.
120	(therap* adj3 touch*).ti,ab.
121	(node? Adj3 link* adj3 map*).ti,ab.
122	or/19-121
123	18 and 122
124	limit 123 to yr="2000 -Current"

Database: International Health Technology Assessment Database (INAHTA)

#	Searches
	All:(gamble or gambler or gamblers or gambling or gambled or betting or bet or bets or wager or wagers)
	AND Publication Year: 2000-2022

Database: Medline and Medline In-Process [Ovid]

#	Searches
1	GAMBLING/
2	gambl*.ti,ab.
3	betting.ti,ab.
4	(bet or bets).ti,ab.
5	wager*.ti,ab.
6	((gaming or gambling or slot or fruit or poker or lottery or lotteries) adj5 (machine? Or terminal?)).ti,ab.
7	(pokies or pokey or puggy or fruities).ti,ab.
8	((dice or card? Or roulette or blackjack or poker or baccarat or crap or craps or keno or casino? Or bingo or bookmaker? Or book maker or bookie? Or lottery or lotteries or lotto or scratch card? Or scratchcard? Or raffle or raffles or sweepstak * or amusement arcade? Or slot?) adj5 (money or monetization or monetisation or monetary or currency or currencies or cryptocurrency or cryptocurrencies or reward* or win or wins or winning* or loss or losses or lose)).ti,ab.
9	((game or games or gaming or gamer?) adj5 (money or monetization or monetisation or monetary)).ti,ab.
10	(loot box* or lootbox*).ti,ab.
11	or/1-10
12	exp PSYCHOTHERAPY/
13	exp MIND-BODY THERAPIES/
14	(psycho* adj5 (intervention? Or treat* or therap*)).ti,ab.
15	((cogniti* or behavio*) adj5 (intervention? Or treat* or therap* or technique?)).ti,ab.
16	CBT.ti,ab.
17	(aversi* adj3 (therap* or treat* or learn*)).ti,ab.
18	(psychotherap * or psychodynamic? Or psychoanal*).ti,ab.
19	exp COUNSELING/
20	counsel*.ti,ab.
21	(motivat * adj3 interview*).ti,ab.
22	HARM REDUCTION/
23	(harm* adj3 (reduc* or minimi*)).ti,ab.
24	exp PSYCHODRAMA/
25	(psychodrama* or psycho drama*).ti,ab.
26	(drama* adj3 therap*).ti,ab.
27	dramatherap*.ti,ab.
28	(addict* adj5 (intervention? Or treat* or therap* or rehab*)).ti,ab.
29	(trauma* adj5 (intervention? Or treat* or therap* or rehab*)).ti,ab.
30	(eye? Adj3 mov* adj5 (desensiti* or de-sensiti* or process* or reprocess* or therap* or program* or reprogram*)).ti,ab.
31	EMDR.ti,ab.
32	hypno*.ti,ab.
33	ELECTRIC STIMULATION/
34	ELECTRIC STIMULATION THERAPY/
35	TRANSCRANIAL MAGNETIC STIMULATION/
36	TRANSCRANIAL DIRECT CURRENT STIMULATION/
37	DEEP BRAIN STIMULATION/
38	(stimulat* adj5 (intervention? Or treat* or therap* or rehab*)).ti,ab.
39	((neurolo* or brain? Or transcranial*) adj3 stimulat*).ti,ab.
40	neuromodulat *.ti,ab.
41	TMS.ti,ab.
42	(cognit* adj3 bias* adj3 modif*).ti,ab.
43	((resident* or inpatient) adj5 (intervention? Or treat* or therap* or rehab*)).ti,ab.
44	retreat?.ti,ab.
45	SELF-MANAGEMENT/
46	SELF CARE/
47	SELF-HELP GROUPS/
48	(self adj5 (help* or care or manag* or direct* or guid*)).ti,ab.
49	INTERNET-BASED INTERVENTION/
50	((digital* or computer* or online or web or internet or tele* or mobile or phone? Or app?) adj5 intervention?).ti,ab.
51	HOTLINES/
52	(hotline? Or helpline? Or help line?).ti,ab.
53	(web adj3 (service? Or support*)).ti,ab.
54	(personali* adj3 (feedback or feed back or intervention?)).ti,ab.
55	gamif*.ti,ab.
56	((psychosocial* or psycho social*) adj5 (intervention? Or treat* or therap* or rehab*)).ti,ab.
57	SOCIAL SKILLS/
58	ASSERTIVENESS/

#	Searches
59	((life or social*) adj3 skill? Adj5 (intervention? Or treat* or therap* or rehab* or train*)).ti,ab.
60	((assertive* or function* or communicat*) adj5 (intervention? Or treat* or therap* or rehab* or train*)).ti,ab.
61	((parent? Or parental or mother? Or father? Or son? Or daughter? Or sibling? Or brother? Or sister? Or grandparent? Or grandfather? Or grandmother? Or family or families or relatives or cousin? Or uncle? Or aunt? Or auntie? Or caregiver? Or carer? Or friend? Or spouse? Or husband? Or wife or wives or couple or couples or partner or partners or boyfriend? Or girlfriend?) adj5 (intervention? Or treat* or therap* or rehab* or train* or model?)).ti,ab.
62	((affected or significant) adj3 other? Adj5 (intervention? Or treat* or therap* or rehab* or train* or model?)).ti,ab.
63	(loved one* adj5 (intervention? Or treat* or therap* or rehab* or train* or model?)).ti,ab.
64	COMMUNITY SUPPORT/
65	SOCIAL SUPPORT/
66	((communit* or neighbo?r* or religious* or social* or cultur* or ethnic*) adj5 support*).ti,ab.
67	(support* adj5 (organization? Or organisation? Or program* or group?)).ti,ab.
68	(peer? Adj3 (support* or intervention*)).ti,ab.
69	(SMART adj3 recover*).ti,ab.
70	SECONDARY PREVENTION/
71	((prevent* or avoid*) adj5 (relaps* or recur* or dropout or drop* out or second*)).ti,ab.
72	(recover* adj3 capital*).ti,ab.
73	(mutual* adj3 aid*).ti,ab.
74	AFTERCARE/
75	((after* or followup or follow* up) adj3 care).ti,ab.
76	aftercare.ti,ab.
77	((followup or follow* up) adj3 treat*).ti,ab.
78	(support* adj5 therap*).ti,ab.
79	MINDFULNESS/
80	mindful*.ti,ab.
81	(self adj3 (compass* or forgiv*)).ti,ab.
82	MENTORING/
83	MENTORS/
84	mentor*.ti,ab.
85	((marital* or marriage?) adj5 therap*).ti,ab.
86	(systemic* adj5 (intervention? Or treat* or therap* or rehab* or model?)).ti,ab.
87	BANKING, PERSONAL/
88	BUDGETS/
89	((financ * or bank* or money or spend* or cash or budget*) adj5 (intervention? Or manag* or plan* or train* or educat* or limit* or restrict*)).ti,ab.
90	((self or volunt*) adj5 exclu*).ti,ab.
91	(access* adj3 restrict*).ti,ab.
92	Gamban.ti,ab.
93	((financ * or bank* or money or spend* or cash or budget*) adj5 (digital* or computer* or online or web or internet or tele* or mobile or phone? Or app?)).ti,ab.
94	(support* adj5 (react* or mechanism? Or intervention? Or model*)).ti,ab.
95	(post adj5 (resident* or treat* or intervention? Or therap* or rehab*)).ti,ab.
96	(postresident* or posttreat* or postintervention? Or posttherap * or postrehab*).ti,ab.
97	(relaps* adj5 (program* or educat* or train* or learn* or teach* or volunteer* or occupation* or work* or job? Or employ* or intervention?)).ti,ab.
98	(recover* adj3 college?).ti,ab.
99	((animal? Or equine or art or anger or acceptance or commitment? Or implosive or virtual reality or relaxation or dance or emotion* or gestalt or horticultur* or mentali?ation or music* or narrativ * or play or reality or schema or socioenvironmental or socio environmental or milieu or mind body or laugh*) adj3 therap*).ti,ab.
100	(chronotherapy* or bibliotherapy* or logotherap*).ti,ab.
101	(cognitive adj3 (restructur* or remediat* or refram *)).ti,ab.
102	(feedback adj3 (psycholog* or sensory or neuro*)).ti,ab.
103	(biofeedback or neurofeedback).ti,ab.
104	((autogenic or sensitivity or desensiti?ation or sensiti?ation) adj3 train*).ti,ab.
105	meditat *.ti,ab.
106	(cris?s adj3 intervention?).ti,ab.
107	(transaction* adj3 analys*).ti,ab.
108	role play*.ti,ab.
109	(breath* adj3 (exercis * or therap*)).ti,ab.
110	(qigong or tai ji or tai chi or yoga).ti,ab.
111	(mental* adj3 (heal? Or healing)).ti,ab.
112	(therap* adj3 touch*).ti,ab.
113	(node? Adj3 link* adj3 map*).ti,ab.
114	or/12-113
115	11 and 114
116	limit 115 to english language
117	limit 116 to yr="2000 -Current"
118	LETTER/
119	EDITORIAL/

#	Searches
120	NEWS/
121	exp HISTORICAL ARTICLE/
122	ANECDOTES AS TOPIC/
123	COMMENT/
124	CASE REPORT/
125	(letter or comment*).ti.
126	or/118-125
127	RANDOMIZED CONTROLLED TRIAL/ or random*.ti,ab.
128	126 not 127
129	ANIMALS/ not HUMANS/
130	exp ANIMALS, LABORATORY/
131	exp ANIMAL EXPERIMENTATION/
132	exp MODELS, ANIMAL/
133	exp RODENTIA/
134	(rat or rats or mouse or mice).ti.
135	or/128-134
136	117 not 135
137	META-ANALYSIS/
138	META-ANALYSIS AS TOPIC/
139	(meta analy* or metanaly* or metaanaly*).ti,ab.
140	((systematic* or evidence*) adj2 (review* or overview*)).ti,ab.
141	(reference list* or bibliograph* or hand search* or manual search* or relevant journals).ab.
142	(search strategy or search criteria or systematic search or study selection or data extraction).ab.
143	(search* adj4 literature).ab.
144	(medline or pubmed or cochrane or embase or psychlit or psyclit or psychinfo or psycinfo or cinahl or science citation index or bids or cancerlit).ab.
145	cochrane.jw.
146	or/137-145
147	randomized controlled trial.pt.
148	controlled clinical trial.pt.
149	pragmatic clinical trial.pt.
150	randomi#ed.ab.
151	placebo.ab.
152	randomly.ab.
153	CLINICAL TRIALS AS TOPIC/
154	trial.ti.
155	or/147-154
156	exp EPIDEMIOLOGIC STUDIES/ or exp CLINICAL TRIAL/ or COMPARATIVE STUDY/
157	(control and study).mp.
158	program.mp.
159	or/156-158
160	(ANIMALS/ not HUMANS/) or COMMENT/ or EDITORIAL/ or exp REVIEW/ or META ANALYSIS/ or CONSENSUS/ or exp GUIDELINE/
161	hi.fs. or case report.mp.
162	or/160-161
163	159 not 162
164	136 and 146
165	136 and 155
166	136 and 163
167	or/164-166

Database: PsycInfo [Ovid]

#	Searches
1	GAMBLING/
2	GAMBLING DISORDER/
3	gambl*.ti,ab.
4	betting.ti,ab.
5	(bet or bets).ti,ab.
6	wager*.ti,ab.
7	((gaming or gambling or slot or fruit or poker or lottery or lotteries) adj5 (machine? Or terminal?)).ti,ab.
8	(pokies or pokey or puggy or fruities).ti,ab.
9	((dice or card? Or roulette or blackjack or poker or baccarat or crap or craps or keno or casino? Or bingo or bookmaker? Or book maker or bookie? Or lottery or lotteries or lotto or scratch card? Or scratchcard? Or raffle or raffles or sweepstak * or amusement arcade? Or slot?) adj5 (money or monetization or monetisation or monetary or currency or currencies or cryptocurrency or cryptocurrencies or reward* or win or wins or winning* or loss or losses or lose)).ti,ab.
10	((game or games or gaming or gamer?) adj5 (money or monetization or monetisation or monetary)).ti,ab.
11	(loot box* or lootbox*).ti,ab.
12	or/1-11

#	Searches
13	exp PSYCHOTHERAPY/
14	exp ADDICTION TREATMENT/
15	exp COGNITIVE BEHAVIOR THERAPY/
16	exp COGNITIVE TECHNIQUES/
17	exp CREATIVE ARTS THERAPY/
18	exp MAINTENANCE THERAPY/
19	exp RELAXATION THERAPY/
20	SOCIOTHERAPY/
21	TRAUMA-INFORMED CARE/
22	exp ALTERNATIVE MEDICINE/
23	MIND BODY THERAPY/
24	(psycho* adj5 (intervention? Or treat* or therap*)).ti,ab.
25	((cogniti* or behavio*) adj5 (intervention? Or treat* or therap* or technique?)).ti,ab.
26	CBT.ti,ab.
27	(aversi* adj3 (therap* or treat* or learn*)).ti,ab.
28	(psychotherap * or psychodynamic? Or psychoanal*).ti,ab.
29	exp COUNSELING/
30	counsel*.ti,ab.
31	(motivat * adj3 interview*).ti,ab.
32	HARM REDUCTION/
33	(harm* adj3 (reduc* or minimi*)).ti,ab.
34	(psychodrama* or psycho drama*).ti,ab.
35	(drama* adj3 therap*).ti,ab.
36	dramatherap*.ti,ab.
37	(addict* adj5 (intervention? Or treat* or therap* or rehab*)).ti,ab.
38	(trauma* adj5 (intervention? Or treat* or therap* or rehab*)).ti,ab.
39	(eye? Adj3 mov* adj5 (desensiti* or de-sensiti* or process* or reprocess* or therap* or program* or reprogram*)).ti,ab.
40	EMDR.ti,ab.
41	exp HYPNOSIS/
42	hypno*.ti,ab.
43	ELECTRICAL STIMULATION/
44	exp ELECTRICAL BRAIN STIMULATION/
45	TRANSCRANIAL MAGNETIC STIMULATION/
46	(stimulat* adj5 (intervention? Or treat* or therap* or rehab*)).ti,ab.
47	((neurolo* or brain? Or transcranial*) adj3 stimulat*).ti,ab.
48	neuromodulat *.ti,ab.
49	TMS.ti,ab.
50	(cognit* adj3 bias* adj3 modif*).ti,ab.
51	((resident* or inpatient) adj5 (intervention? Or treat* or therap* or rehab*)).ti,ab.
52	retreat?.ti,ab.
53	SELF-HELP TECHNIQUES/
54	exp SELF MANAGEMENT/
55	SELF-CARE/
56	(self adj5 (help* or care or manag* or direct* or guid*)).ti,ab.
57	DIGITAL INTERVENTIONS/
58	((digital* or computer* or online or web or internet or tele* or mobile or phone? Or app?) adj5 intervention?).ti,ab.
59	HOT LINE SERVICES/
60	(hotline? Or helpline? Or help line?).ti,ab.
61	(web adj3 (service? Or support*)).ti,ab.
62	(personali* adj3 (feedback or feed back or intervention?)).ti,ab.
63	gamif*.ti,ab.
64	((psychosocial* or psycho social*) adj5 (intervention? Or treat* or therap* or rehab*)).ti,ab.
65	SOCIAL SKILLS TRAINING/
66	ASSERTIVENESS TRAINING/
67	((life or social*) adj3 skill? Adj5 (intervention? Or treat* or therap* or rehab* or train*)).ti,ab.
68	((assertive* or function* or communicat*) adj5 (intervention? Or treat* or therap* or rehab* or train*)).ti,ab.
69	((parent? Or parental or mother? Or father? Or son? Or daughter? Or sibling? Or brother? Or sister? Or grandparent? Or grandfather? Or grandmother? Or family or families or relatives or cousin? Or uncle? Or aunt? Or auntie? Or caregiver? Or carer? Or friend? Or spouse? Or husband? Or wife or wives or couple or couples or partner or partners or boyfriend? Or girlfriend?) adj5 (intervention? Or treat* or therap* or rehab* or train* or model?)).ti,ab.
70	((affected or significant) adj3 other? Adj5 (intervention? Or treat* or therap* or rehab* or train* or model?)).ti,ab.
71	(loved one* adj5 (intervention? Or treat* or therap* or rehab* or train* or model?)).ti,ab.
72	SOCIAL SUPPORT/
73	SUPPORT GROUPS/
74	((communit* or neighbo?r* or religious* or social* or cultur* or ethnic*) adj5 support*).ti,ab.
75	(support* adj5 (organization? Or organisation? Or program* or group?)).ti,ab.
76	(peer? Adj3 (support* or intervention*)).ti,ab.
77	(SMART adj3 recover*).ti,ab.
78	RELAPSE PREVENTION/

#	Searches
79	((prevent* or avoid*) adj5 (relaps* or recur* or dropout or drop* out or second*)).ti,ab.
80	(recover* adj3 capital*).ti,ab.
81	(mutual* adj3 aid*).ti,ab.
82	AFTERCARE/
83	((after* or followup or follow* up) adj3 care).ti,ab.
84	aftercare.ti,ab.
85	POSTTREATMENT FOLLOWUP/
86	((followup or follow* up) adj3 treat*).ti,ab.
87	(support* adj5 therap*).ti,ab.
88	MINDFULNESS/
89	MINDFULNESS-BASED INTERVENTIONS/
90	mindful*.ti,ab.
91	SELF COMPASSION/
92	(self adj3 (compass* or forgiv*)).ti,ab.
93	MENTOR/
94	mentor*.ti,ab.
95	((marital* or marriage?) adj5 therap*).ti,ab.
96	(systemic* adj5 (intervention? Or treat* or therap* or rehab* or model?)).ti,ab.
97	BANKING/
98	PERSONAL FINANCE/
99	BUDGETS/
100	((financ * or bank* or money or spend* or cash or budget*) adj5 (intervention? Or manag* or plan* or train* or educat* or limit* or restrict*)).ti,ab.
101	((self or volunt*) adj5 exclu*).ti,ab.
102	(access* adj3 restrict*).ti,ab.
103	Gamban.ti,ab.
104	((financ * or bank* or money or spend* or cash or budget*) adj5 (digital* or computer* or online or web or internet or tele* or mobile or phone? Or app?)).ti,ab.
105	(support* adj5 (react* or mechanism? Or intervention? Or model*)).ti,ab.
106	(post adj5 (resident* or treat* or intervention? Or therap* or rehab*)).ti,ab.
107	(postresident* or posttreat* or postintervention? Or posttherap * or postrehab*).ti,ab.
108	(relaps* adj5 (program* or educat* or train* or learn* or teach* or volunteer* or occupation* or work* or job? Or employ* or intervention?)).ti,ab.
109	(recover* adj3 college?).ti,ab.
110	((animal? Or equine or art or anger or acceptance or commitment? Or implosive or virtual reality or relaxation or dance or emotion* or gestalt or horticultur* or mentali?ation or music* or narrativ * or play or reality or schema or socioenvironmental or socio environmental or milieu or mind body or laugh*) adj3 therap*).ti,ab.
111	(chronotherapy* or bibliotherapy* or logotherap*).ti,ab.
112	(cognitive adj3 (restructur* or remediat* or refram *)).ti,ab.
113	(feedback adj3 (psycholog* or sensory or neuro*)).ti,ab.
114	(biofeedback or neurofeedback).ti,ab.
115	((autogenic or sensitivity or desensiti?ation or sensiti?ation) adj3 train*).ti,ab.
116	MEDITATION/
117	meditat *.ti,ab.
118	CRISIS INTERVENTION/
119	(cris?s adj3 intervention?).ti,ab.
120	(transaction* adj3 analys*).ti,ab.
121	ROLE PLAYING/
122	ROLE PLAYING GAMES/
123	role play*.ti,ab.
124	(breath* adj3 (exercis * or therap*)).ti,ab.
125	YOGA/
126	(qigong or tai ji or tai chi or yoga).ti,ab.
127	(mental* adj3 (heal? Or healing)).ti,ab.
128	(therap* adj3 touch*).ti,ab.
129	(node? Adj3 link* adj3 map*).ti,ab.
130	or/13-129
131	12 and 130
132	limit 131 to english language
133	limit 132 to yr="2000 -Current"
134	(meta analysis or "systematic review").md. or META ANALYSIS/ or "SYSTEMATIC REVIEW"/
135	(meta analy* or metanaly* or metaanaly*).ti,ab.
136	((systematic* or evidence*) adj2 (review* or overview*)).ti,ab.
137	(reference list* or bibliograph* or hand search* or manual search* or relevant journals).ab.
138	(search strategy or search criteria or systematic search or study selection or data extraction).ab.
139	(search* adj4 literature).ab.
140	cochrane.jw.
141	((pool* or combined) adj2 (data or trials or studies or results)).ab.
142	(medline or pubmed or cochrane or embase or psychlit or psyclit or cinahl or science citation index or bids or cancerlit).ab.

#	Searches
143	or/134-142
144	clinical trial.md. or Clinical trials/ or Randomized controlled trials/ or Randomized clinical trials/ or (assign* or allocat* or crossover* or cross over* or ((doubl* or singl*) adj blind*) or factorial* or placebo* or random* or volunteer* or trial?).ti,ab.
145	EPIDEMIOLOGY/ or PROSPECTIVE STUDIES/ or RETROSPECTIVE STUDIES/ or COHORT ANALYSIS/ or FOLLOWUP STUDIES/ or exp CLINICAL TRIALS/
146	(control and study).mp.
147	program.mp.
148	or/145-147
149	133 and 143
150	133 and 144
151	133 and 148
152	or/149-151
153	limit 152 to (“0100 journal” or “0110 peer-reviewed journal”)

Database: Social Care Online

#	Searches
	AllFields:’gamble or gambler or gamblers or gambling or gambled or betting or bet or bets or wager or wagers or “gaming machine” or “slot machine” or “fruit machine” or “poker machine” or “lottery machine” or “lotteries machine” or “gaming terminal” or “slot terminal” or “fruit terminal” or “poker terminal” or “lottery terminal” or “lotteries terminal” or pokies or pokey or puggy or fruities’
	AND AllFields:’psychological or therapy or therapies or psychotherapy or cognitive or behavioural or behavioral or CBT or counsel or counselling or “motivational interviewing” or “harm reduction” or Psychodrama or dramatherapy or “eye movement” or EMDR or hypnosis or “electric stimulation” or electrostimulation or electrotherapy or transcranial or “brain stimulation” or neuromodulation or TMS or “cognitive bias modification” or retreat or retreats or “self help” or “self care” or “self management” or “self directed” or “self guided” or “web based” or “internet based” or “phone based” or app or hotline or helpline or “help line” or “web support” or “personalized feedback” or “personalized feed back” or gamification or psychosocial or “psycho social” or “social skill” or assertiveness or “community support” or “social support” or “support program” or “support group” or “peer support” or “SMART recovery” or “relapse prevention” or “prevent relapse” or “secondary prevention” or “recovery capital” or “mutual aid” or “after care” or aftercare or “followup treatment” or “follow up treatment” or mindfulness or “self compassion” or mentor or mentoring or “systemic intervention” or finance or banking or budget or “self exclusion” or “voluntary exclusion” or “restricting access” or Gamban or “support mechanism” or “support model” or “post resident” or postresident or “post treatment” or posttreatment or “relapse program” or “recovery college” or “cognitive restructuring” or biofeedback or neurofeedback or “autogenic training” or meditate or meditation or “crisis intervention” or “transaction analysis” or “role play” or “role playing” or “breathing exercise” or qigong or “tai ji” or “tai chi” or yoga or “therapeutic touch” or “node link mapping”’
	AND PublicationYear:’2000 2022’

Database: Social Policy and Practice (SPP) [Ovid]

#	Searches
1	gambl*.ti,ab.
2	betting.ti,ab.
3	(bet or bets).ti,ab.
4	wager*.ti,ab.
5	((gaming or gambling or slot or fruit or poker or lottery or lotteries) adj5 (machine? Or terminal?)).ti,ab.
6	(pokies or pokey or puggy or fruities).ti,ab.
7	((dice or card? Or roulette or blackjack or poker or baccarat or crap or craps or keno or casino? Or bingo or bookmaker? Or book maker or bookie? Or lottery or lotteries or lotto or scratch card? Or scratchcard? Or raffle or raffles or sweepstak * or amusement arcade? Or slot?) adj5 (money or monetization or monetisation or monetary or currency or currencies or cryptocurrency or cryptocurrencies or reward* or win or wins or winning* or loss or losses or lose)).ti,ab.
8	((game or games or gaming or gamer?) adj5 (money or monetization or monetisation or monetary)).ti,ab.
9	(loot box* or lootbox*).ti,ab.
10	or/1-9
11	(psycho* adj5 (intervention? Or treat* or therap*)).ti,ab.
12	((cogniti* or behavio*) adj5 (intervention? Or treat* or therap* or technique?)).ti,ab.
13	CBT.ti,ab.
14	(aversi* adj3 (therap* or treat* or learn*)).ti,ab.
15	(psychotherap * or psychodynamic? Or psychoanal*).ti,ab.
16	counsel*.ti,ab.
17	(motivat * adj3 interview*).ti,ab.
18	(harm* adj3 (reduc* or minimi*)).ti,ab.
19	(psychodrama* or psycho drama*).ti,ab.
20	(drama* adj3 therap*).ti,ab.
21	dramatherap*.ti,ab.
22	(addict* adj5 (intervention? Or treat* or therap* or rehab*)).ti,ab.
23	(trauma* adj5 (intervention? Or treat* or therap* or rehab*)).ti,ab.
24	(eye? Adj3 mov* adj5 (desensiti* or de-sensiti* or process* or reprocess* or therap* or program* or reprogram*)).ti,ab.
25	EMDR.ti,ab.
26	hypno*.ti,ab.
27	(stimulat* adj5 (intervention? Or treat* or therap* or rehab*)).ti,ab.

#	Searches
28	((neurolo* or brain? Or transcranial*) adj3 stimulat*).ti,ab.
29	neuromodulat *.ti,ab.
30	TMS.ti,ab.
31	(cognit* adj3 bias* adj3 modif*).ti,ab.
32	((resident* or inpatient) adj5 (intervention? Or treat* or therap* or rehab*)).ti,ab.
33	retreat?.ti,ab.
34	(self adj5 (help* or care or manag* or direct* or guid*)).ti,ab.
35	((digital* or computer* or online or web or internet or tele* or mobile or phone? Or app?) adj5 intervention?).ti,ab.
36	(hotline? Or helpline? Or help line?).ti,ab.
37	(web adj3 (service? Or support*)).ti,ab.
38	(personali* adj3 (feedback or feed back or intervention?)).ti,ab.
39	gamif*.ti,ab.
40	((psychosocial* or psycho social*) adj5 (intervention? Or treat* or therap* or rehab*)).ti,ab.
41	((life or social*) adj3 skill? Adj5 (intervention? Or treat* or therap* or rehab* or train*)).ti,ab.
42	((assertive* or function* or communicat*) adj5 (intervention? Or treat* or therap* or rehab* or train*)).ti,ab.
43	((parent? Or parental or mother? Or father? Or son? Or daughter? Or sibling? Or brother? Or sister? Or grandparent? Or grandfather? Or grandmother? Or family or families or relatives or cousin? Or uncle? Or aunt? Or auntie? Or caregiver? Or carer? Or friend? Or spouse? Or husband? Or wife or wives or couple or couples or partner or partners or boyfriend? Or girlfriend?) adj5 (intervention? Or treat* or therap* or rehab* or train* or model?)).ti,ab.
44	((affected or significant) adj3 other? Adj5 (intervention? Or treat* or therap* or rehab* or train* or model?)).ti,ab.
45	(loved one* adj5 (intervention? Or treat* or therap* or rehab* or train* or model?)).ti,ab.
46	((communit* or neighbo?r* or religious* or social* or cultur* or ethnic*) adj5 support*).ti,ab.
47	(support* adj5 (organization? Or organisation? Or program* or group?)).ti,ab.
48	(peer? Adj3 (support* or intervention*)).ti,ab.
49	(SMART adj3 recover*).ti,ab.
50	((prevent* or avoid*) adj5 (relaps* or recur* or dropout or drop* out or second*)).ti,ab.
51	(recover* adj3 capital*).ti,ab.
52	(mutual* adj3 aid*).ti,ab.
53	((after* or followup or follow* up) adj3 care).ti,ab.
54	aftercare.ti,ab.
55	((followup or follow* up) adj3 treat*).ti,ab.
56	(support* adj5 therap*).ti,ab.
57	mindful*.ti,ab.
58	(self adj3 (compass* or forgiv*)).ti,ab.
59	mentor*.ti,ab.
60	((marital* or marriage?) adj5 therap*).ti,ab.
61	(systemic* adj5 (intervention? Or treat* or therap* or rehab* or model?)).ti,ab.
62	((financ * or bank* or money or spend* or cash or budget*) adj5 (intervention? Or manag* or plan* or train* or educat* or limit* or restrict*)).ti,ab.
63	((self or volunt*) adj5 exclu*).ti,ab.
64	(access* adj3 restrict*).ti,ab.
65	Gamban.ti,ab.
66	((financ * or bank* or money or spend* or cash or budget*) adj5 (digital* or computer* or online or web or internet or tele* or mobile or phone? Or app?)).ti,ab.
67	(support* adj5 (react* or mechanism? Or intervention? Or model*)).ti,ab.
68	(post adj5 (resident* or treat* or intervention? Or therap* or rehab*)).ti,ab.
69	(postresident* or posttreat* or postintervention? Or posttherap * or postrehab*).ti,ab.
70	(relaps* adj5 (program* or educat* or train* or learn* or teach* or volunteer* or occupation* or work* or job? Or employ* or intervention?)).ti,ab.
71	(recover* adj3 college?).ti,ab.
72	((animal? Or equine or art or anger or acceptance or commitment? Or implosive or virtual reality or relaxation or dance or emotion* or gestalt or horticultur* or mentali?ation or music* or narrativ * or play or reality or schema or socioenvironmental or socio environmental or milieu or mind body or laugh*) adj3 therap*).ti,ab.
73	(chronotherapy* or bibliotherapy* or logotherap*).ti,ab.
74	(cognitive adj3 (restructur* or remediat* or refram *)).ti,ab.
75	(feedback adj3 (psycholog* or sensory or neuro*)).ti,ab.
76	(biofeedback or neurofeedback).ti,ab.
77	((autogenic or sensitivity or desensiti?ation or sensiti?ation) adj3 train*).ti,ab.
78	meditat *.ti,ab.
79	(cris?s adj3 intervention?).ti,ab.
80	(transaction* adj3 analys*).ti,ab.
81	role play*.ti,ab.
82	(breath* adj3 (exercis * or therap*)).ti,ab.
83	(qigong or tai ji or tai chi or yoga).ti,ab.
84	(mental* adj3 (heal? Or healing)).ti,ab.
85	(therap* adj3 touch*).ti,ab.
86	(node? Adj3 link* adj3 map*).ti,ab.
87	or/11-86
88	10 and 87

#	Searches
89	limit 88 to yr="2000 -Current"

Database: Social Science Citation Index (SSCI) [Web of Science]

#	Searches
	(gambl* or betting or bet or bets or wager* or "gaming machine*" or "slot machine*" or "fruit machine*" or "poker machine*" or "lottery machine*" or "lotteries machine*" or "gaming terminal*" or "slot terminal*" or "fruit terminal*" or "poker terminal*" or "lottery terminal*" or "lotteries terminal*" or pokies or pokey or puggy or fruities) and (psycho* or therap* or psychotherap * or cognitive or behavioural or behavioral or CBT or aversi* or counsel* or "motivational interview*" or "harm reduction" or Psychodrama or dramatherap* or "eye movement" or EMDR or hypno* or "electric stimulat*" or electrostimulat* or electrotherapy or transcranial* or "brain stimulation" or neuromodulat * or TMS or "cognitive bias modification" or retreat or retreats or "self help" or "self care" or "self manage*" or "self directed" or "self guided" or "web based" or "internet based" or "phone based" or app or apps or hotline* or helpline* or "help line*" or "web support*" or "personali* feedback" or "personali* feed back" or gamif* or psychosocial or "psycho social" or "social skill" or "social skills" or assertiveness or "community support" or "social support" or "support program*" or "support group*" or "peer support" or "SMART recovery" or "relapse prevention" or "prevent* relapse" or "secondary prevention" or "recovery capital" or "mutual aid" or "after care" or aftercare or "followup treatment" or "follow up treatment" or "support therapy" or mindfulness or "self compassion" or mentor* or "systemic* intervention*" or finance* or banking or budget* or "self exclu*" or "voluntary exclu*" or "restrict* access" or Gamban or "support mechanism*" or "support model*" or "post resident*" or postresident* or "post treatment" or posttreatment or "relapse program*" or "recovery college*" or "cognitive restructuring*" or biofeedback or neurofeedback or "autogenic training" or meditate or meditation or "crisis intervention*" or "transaction analysis" or "role play" or "role playing" or "breathing exercise*" or qigong or "tai ji" or "tai chi" or yoga or "therapeutic touch" or "node link mapping") (Title) Timespan: 2000-01-01 to 2022-07-01

Grey literature searches

Date of last search: 07/11/2022

Source	Link	Search terms
Kings Fund reports	https://www.kingsfund.org.uk/publications	gambling or betting or bet
Campbell Collaboration	https://onlinelibrary.wiley.com/journal/18911803	gambling or betting or bet
Gov.uk	https://www.gov.uk/	gambling or betting or bet
National Grey Literature Collection	https://allcatsrgrey.org.uk/wp/	gambling or betting or bet
Be gamble aware	https://www.begambleaware.org/for-professionals	psychotherapy or psychosocial or therapy or relapse
GamCare	https://www.gamcare.org.uk/	psychotherapy or psychosocial or therapy or relapse
Gambling Research Exchange Ontario	https://www.greo.ca/en/index.aspx	psychotherapy or psychosocial or therapy or relapse
Gambling Commission	https://www.gamblingcommission.gov.uk/	psychotherapy or psychosocial or therapy or relapse
Advisory Board for Safer Gambling	https://www.gamblingcommission.gov.uk/about-us/transparency/advisory-board-for-safer-gambling	psychotherapy or psychosocial or therapy or relapse
Gambling Watch UK	https://www.gamblingwatchuk.org/	psychotherapy or psychosocial or therapy or relapse
Australian Gambling Research Centre	https://aifs.gov.au/agrc/	psychotherapy or psychosocial or therapy or relapse
Gambling Compliance	https://vixio.com/gamblingcompliance/	psychotherapy or psychosocial or therapy or relapse

Gambling and Addictions Research Centre	https://garc.aut.ac.nz/	psychotherapy or psychosocial or therapy or relapse
Responsible Gambling Council	https://www.responsiblegambling.org/	psychotherapy or psychosocial or therapy or relapse
Victorian Responsible Gambling Foundation	https://responsiblegambling.vic.gov.au/	psychotherapy or psychosocial or therapy or relapse

SUPPLEMENTARY MATERIAL S2: Gambling symptom severity scales

- Addiction Severity Index-Gambling subscale (ASI-G)
- Canadian Problem Gambling Index (CPGI)
- Gambling Problems Index (GPI)
- Gambling Symptom Assessment Scale (GSAS)
- Pathological Gambling Adaptation of Yale-Brown Obsessive Compulsive Scale (PG-Y BOCS)
- Problem Gambling Severity Index (PGSI)
- South Oaks Gambling Screen (SOGS)
- Victorian Gambling Screen (VGS) harm to self subscale

SUPPLEMENTARY MATERIAL S3: WinBUGS codes

WinBUGS code for Symptom Severity, Random Study Effect, Random Class Effect Model

```
# Normal likelihood, identity link
# Random study effects, random intervention within class effects

model{                              # *** PROGRAM STARTS
#Likelihood
for(i in 1:ndata){                      #   LOOP THROUGH STUDIES
   w[i,1] <- 0      # adjustment for multi-arm trials is zero for control arm
   delta[i,1] <- 0            # treatment effect is zero for control arm
                    mu[i] ~ dnorm(0,.0001)         # vague priors for all baselines
   for (k in 1:na[i]) {             #  LOOP THROUGH ARMS
      prec[i,k] <- N[i,k]/pow(sdCFB[i,k],2)     # set precisions
                                       y[i,k]<- meanCFB[i,k]
      y[i,k] ~ dnorm(theta[i,k],prec[i,k]) # normal likelihood
# model for linear predictor
                                       theta[i,k]<-phi[i,k]*sd.pooled[i]
      phi[i,k] <- mu[i] + eta[i,k]
#Set relative effect for outcome 2 equal to outcome 1 for same study
#Pools outcomes from same study
                                       eta[i,k]<- delta[i-out[i]+1,k]
#Deviance contribution
      dev[i,k] <- (y[i,k]-theta[i,k])*(y[i,k]-theta[i,k])*prec[i,k]
     }
#  summed residual deviance contribution for this trial/outcome
   resdev[i] <- sum(dev[i,1:na[i]])
  }
totresdev <- sum(resdev[])           #Total Residual Deviance

#RE Model
for(i in 1:ndata){                   # LOOP THROUGH STUDIES WITH ARM DATA
   for (k in 2:na[i]) {            # LOOP THROUGH ARMS
# trial-specific RE distributions
#Note delta's for outcome 2 datapoints do not contribute to the likelihood, because eta is set
#equal to the delta for outcome 1. #deltas for outcome 2 simply draw from the predictive distribution
      delta[i,k] ~ dnorm(md[i,k],taud[i,k])
# mean of RE distributions, with multi-arm trial correction
      md[i,k] <-  d[t[i,k]] - d[t[i,1]] + sw[i,k]
# precision of RE distributions (with multi-arm trial correction)
      taud[i,k] <- tau *2*(k-1)/k
# adjustment, multi-arm RCTs
      w[i,k] <- (delta[i,k] - d[t[i,k]] + d[t[i,1]])
# cumulative adjustment for multi-arm trials
      sw[i,k] <- sum(w[i,1:k-1])/(k-1)
     }
}

#Random treatment effects within class
d[1]<-0      # treatment effect is zero for reference treatment / class
```

```
D[1]<-0
#Single treatment classes
for (j in 1:n1){
            d[j1[j]]<- D[classd[j1[j]]]
}
#Classes with 2 or more treatments
for (j in 1:nn){
            d[jn[j]]~dnorm(D[classd[jn[j]]],tauD)
}

#Priors
for (k in 2:nc){  D[k] ~ dnorm(0,.0001) }
tau<-1/pow(sd,2)
tauD<-1/pow(sdD,2)
sd~dunif(0,5)
sdD~dunif(0,5)

# SMDs for all possible pair-wise comparisons - Treatments
for (c in 1:(nt-1)) {
            for (k in (c+1):nt) {
      smd[c,k] <- d[k] - d[c]
  }
}

# SMDs for all possible pair-wise comparisons - Classes
for (c in 1:(nc-1)) {
           for (k in (c+1):nc) {
      smdC[c,k] <- D[k] - D[c]
  }
}

#Stop unused variables causing error message
dum[1]<-Industry[1]
dum[2]<-ITT[1]
dum[3]<-CC[1]
dum[4]<-out.type[1]
dum[5]<-class[1,1]
dum[6]<-s[1]
}                                  # *** PROGRAM ENDS
```

WinBUGS code for Symptom Severity, Random Study Effect, Fixed Class Effect Model

```
# Normal likelihood, identity link
# Random study effects, random intervention within class effects

model{                             # *** PROGRAM STARTS
#Likelihood
for(i in 1:ndata){                    #   LOOP THROUGH STUDIES
    w[i,1] <- 0      # adjustment for multi-arm trials is zero for control arm
    delta[i,1] <- 0            # treatment effect is zero for control arm
                        mu[i] ~ dnorm(0,.0001)          # vague priors for all baselines
```

```
   for (k in 1:na[i]) {            #  LOOP THROUGH ARMS
       prec[i,k] <- N[i,k]/pow(sdCFB[i,k],2)     # set precisions
                                                 y[i,k]<- meanCFB[i,k]
       y[i,k] ~ dnorm(theta[i,k],prec[i,k]) # normal likelihood
# model for linear predictor
                                                 theta[i,k]<-phi[i,k]*sd.pooled[i]
       phi[i,k] <- mu[i] + eta[i,k]
#Set relative effect for outcome 2 equal to outcome 1 for same study
#Pools outcomes from same study
                                                 eta[i,k]<- delta[i-out[i]+1,k]
#Deviance contribution
       dev[i,k] <- (y[i,k]-theta[i,k])*(y[i,k]-theta[i,k])*prec[i,k]
      }
#  summed residual deviance contribution for this trial/outcome
   resdev[i] <- sum(dev[i,1:na[i]])
  }
totresdev <- sum(resdev[])           #Total Residual Deviance

#RE Model
for(i in 1:ndata){                  # LOOP THROUGH STUDIES WITH ARM DATA
   for (k in 2:na[i]) {            # LOOP THROUGH ARMS
# trial-specific RE distributions
#Note delta's for outcome 2 datapoints do not contribute to the likelihood, because eta is set
#equal to the delta for outcome 1. #deltas for outcome 2 simply draw from the predictive distribution
       delta[i,k] ~ dnorm(md[i,k],taud[i,k])
# mean of RE distributions, with multi-arm trial correction
       md[i,k] <-  d[t[i,k]] - d[t[i,1]] + sw[i,k]
# precision of RE distributions (with multi-arm trial correction)
       taud[i,k] <- tau *2*(k-1)/k
# adjustment, multi-arm RCTs
       w[i,k] <- (delta[i,k] - d[t[i,k]] + d[t[i,1]])
# cumulative adjustment for multi-arm trials
       sw[i,k] <- sum(w[i,1:k-1])/(k-1)
      }
}

#Fixed treatment effects within class
d[1]<-0       # treatment effect is zero for reference treatment / class
D[1]<-0
#Single treatment classes
for (k in 2:nt){
            d[k]<- D[classd[k]]
}

#Priors
for (k in 2:nc){  D[k] ~ dnorm(0,.0001) }
tau<-1/pow(sd,2)
sd~dunif(0,5)

# SMDs for all possible pair-wise comparisons - Treatments
for (c in 1:(nt-1)) {
```

```
            for (k in (c+1):nt) {
       smd[c,k] <- d[k] - d[c]
  }
}

# SMDs for all possible pair-wise comparisons - Classes
for (c in 1:(nc-1)) {
           for (k in (c+1):nc) {
       smdC[c,k] <- D[k] - D[c]
  }
}

# rank classes
for (k in 1:nc){
  rkC[k] <- rank(D[],k)           # assumes lower values are "good"
            #rk[k] <- nc+1-rank(D[],k)           # assumes higher values are "good"
  bestC[k] <- equals(rkC[k],1)      #calculate probability that treat k is best
  # calculate probability that class k is h-th best
  for (h in 1:nc){ probC[h,k] <- equals(rkC[k],h) }
 }

#Stop unused variables causing error message
dum[1]<-Industry[1]
dum[2]<-ITT[1]
dum[3]<-CC[1]
dum[4]<-out.type[1]
dum[5]<-class[1,1]
dum[6]<-s[1]
}                                # *** PROGRAM ENDS
```

WinBUGS code for Frequency, Random Study Effect, Fixed Class Effect Model

```
# Normal likelihood, identity link
# Random study effects, random intervention within class effects

model{                            # *** PROGRAM STARTS
#Likelihood
for(i in 1:ns){                   #   LOOP THROUGH STUDIES
    w[i,1] <- 0      # adjustment for multi-arm trials is zero for control arm
    delta[i,1] <- 0            # treatment effect is zero for control arm
                      mu[i] ~ dnorm(0,.0001)          # vague priors for all baselines
    for (k in 1:na[i]) {             #  LOOP THROUGH ARMS
        prec[i,k] <- N[i,k]/pow(sdCFB[i,k],2)     # set precisions
                                          y[i,k]<- meanCFB[i,k]
        y[i,k] ~ dnorm(theta[i,k],prec[i,k]) # normal likelihood
# model for linear predictor
                                          theta[i,k]<-phi[i,k]*sd.pooled[i]
        phi[i,k] <- mu[i] + delta[i,k]
#Deviance contribution
        dev[i,k] <- (y[i,k]-theta[i,k])*(y[i,k]-theta[i,k])*prec[i,k]
      }
#  summed residual deviance contribution for this trial/outcome
```

```
   resdev[i] <- sum(dev[i,1:na[i]])
  }
totresdev <- sum(resdev[])            #Total Residual Deviance

#RE Model
for(i in 1:ns){                     # LOOP THROUGH STUDIES WITH ARM DATA
   for (k in 2:na[i]) {             # LOOP THROUGH ARMS
# trial-specific RE distributions
       delta[i,k] ~ dnorm(md[i,k],taud[i,k])
# mean of RE distributions, with multi-arm trial correction
       md[i,k] <-  d[t[i,k]] - d[t[i,1]] + sw[i,k]
# precision of RE distributions (with multi-arm trial correction)
       taud[i,k] <- tau *2*(k-1)/k
# adjustment, multi-arm RCTs
       w[i,k] <- (delta[i,k] - d[t[i,k]] + d[t[i,1]])
# cumulative adjustment for multi-arm trials
       sw[i,k] <- sum(w[i,1:k-1])/(k-1)
      }
}

#Fixed treatment effects within class
d[1]<-0       # treatment effect is zero for reference treatment / class
D[1]<-0
#Single treatment classes
for (k in 2:nt){
            d[k]<- D[classd[k]]
}

#Priors
for (k in 2:nc){  D[k] ~ dnorm(0,.0001) }
tau<-1/pow(sd,2)
sd~dunif(0,5)

# SMDs for all possible pair-wise comparisons - Treatments
for (c in 1:(nt-1)) {
            for (k in (c+1):nt) {
      smd[c,k] <- d[k] - d[c]
  }
}

# SMDs for all possible pair-wise comparisons - Classes
for (c in 1:(nc-1)) {
           for (k in (c+1):nc) {
      smdC[c,k] <- D[k] - D[c]
  }
}

# rank classes
for (k in 1:nc){
  rkC[k] <- rank(D[],k)           # assumes lower values are “good”
            #rk[k] <- nc+1-rank(D[],k)           # assumes higher values are “good”
```

```
  bestC[k] <- equals(rkC[k],1)      #calculate probability that treat k is best
  # calculate probability that class k is h-th best
  for (h in 1:nc){ probC[h,k] <- equals(rkC[k],h) }
 }

#Stop unused variables causing error message
dum[1]<-Industry[1]
dum[2]<-ITT[1]
dum[3]<-units[1]
dum[4]<-out.type[1]
dum[5]<-class[1,1]
dum[6]<-s[1]
dum[7]<-E[1]
}                                # *** PROGRAM ENDS
```

WinBUGS code for Symptom Severity, Unrelated Mean Effects (Inconsistency) Model

```
# Normal likelihood, identity link
# Random study effects, random intervention within class effects

model{                           # *** PROGRAM STARTS
#Likelihood
for(i in 1:ndata){                    #   LOOP THROUGH STUDIES
    w[i,1] <- 0      # adjustment for multi-arm trials is zero for control arm
    delta[i,1] <- 0             # treatment effect is zero for control arm
                      mu[i] ~ dnorm(0,.0001)          # vague priors for all baselines
    for (k in 1:na[i]) {             #  LOOP THROUGH ARMS
        prec[i,k] <- N[i,k]/pow(sdCFB[i,k],2)     # set precisions
                                      y[i,k]<- meanCFB[i,k]
        y[i,k] ~ dnorm(theta[i,k],prec[i,k]) # normal likelihood
# model for linear predictor
                                      theta[i,k]<-phi[i,k]*sd.pooled[i]
        phi[i,k] <- mu[i] + eta[i,k]
#Set relative effect for outcome 2 equal to outcome 1 for same study
#Pools outcomes from same study
                                      eta[i,k]<- delta[i-out[i]+1,k]
#Deviance contribution
        dev[i,k] <- (y[i,k]-theta[i,k])*(y[i,k]-theta[i,k])*prec[i,k]
      }
#  summed residual deviance contribution for this trial/outcome
    resdev[i] <- sum(dev[i,1:na[i]])
  }
totresdev <- sum(resdev[])           #Total Residual Deviance

#RE Model
for(i in 1:ndata){                   # LOOP THROUGH STUDIES WITH ARM DATA
    for (k in 2:na[i]) {            # LOOP THROUGH ARMS
# trial-specific RE distributions
#Note delta's for outcome 2 datapoints do not contribute to the likelihood, because eta is set
#equal to the delta for outcome 1. #deltas for outcome 2 simply draw from the predictive distribution
        delta[i,k] ~ dnorm(md[i,k],taud[i,k])
```

```
# mean of RE distributions, with multi-arm trial correction
        md[i,k] <-  D[classd[t[i,1]],classd[t[i,k]]] + sw[i,k]
# precision of RE distributions (with multi-arm trial correction)
        taud[i,k] <- tau *2*(k-1)/k
# adjustment, multi-arm RCTs
        w[i,k] <- (delta[i,k] - D[classd[t[i,1]],classd[t[i,k]]] )
# cumulative adjustment for multi-arm trials
        sw[i,k] <- sum(w[i,1:k-1])/(k-1)
      }
}

#Fixed treatment effects within class
#Priors
for (c in 1:nc-1){
            D[c,c]<-0
            for (k in (c+1):nc){
            D[c,k]~dnorm(0,.0001)
            D[k,c]<- -D[c,k]
            }
}
D[nc,nc]<-0
tau<-1/pow(sd,2)
sd~dunif(0,5)

#Stop unused variables causing error message
dum[1]<-Industry[1]
dum[2]<-ITT[1]
dum[3]<-CC[1]
dum[4]<-out.type[1]
dum[5]<-class[1,1]
dum[6]<-s[1]
}                              # *** PROGRAM ENDS
```

WinBUGS code for Symptom Severity, Random Study Effect, Fixed Class Effect Model, Meta-Regression for Industry Funding

```
# Normal likelihood, identity link
# Random study effects, random intervention within class effects

model{                         # *** PROGRAM STARTS
#Likelihood
for(i in 1:ndata){                  #   LOOP THROUGH STUDIES
    w[i,1] <- 0      # adjustment for multi-arm trials is zero for control arm
    delta[i,1] <- 0            # treatment effect is zero for control arm
                      mu[i] ~ dnorm(0,.0001)         # vague priors for all baselines
    for (k in 1:na[i]) {            #  LOOP THROUGH ARMS
        prec[i,k] <- N[i,k]/pow(sdCFB[i,k],2)     # set precisions
                                          y[i,k]<- meanCFB[i,k]
        y[i,k] ~ dnorm(theta[i,k],prec[i,k]) # normal likelihood
# model for linear predictor
                                          theta[i,k]<-phi[i,k]*sd.pooled[i]
        phi[i,k] <- mu[i] + eta[i,k]
```

```
#Set relative effect for outcome 2 equal to outcome 1 for same study
#Pools outcomes from same study
                                        eta[i,k]<- delta[i-out[i]+1,k]
#Deviance contribution
      dev[i,k] <- (y[i,k]-theta[i,k])*(y[i,k]-theta[i,k])*prec[i,k]
     }
#  summed residual deviance contribution for this trial/outcome
   resdev[i] <- sum(dev[i,1:na[i]])
  }
totresdev <- sum(resdev[])           #Total Residual Deviance

#RE Model
for(i in 1:ndata){                    # LOOP THROUGH STUDIES WITH ARM DATA
   for (k in 2:na[i]) {             # LOOP THROUGH ARMS
# trial-specific RE distributions
#Note delta's for outcome 2 datapoints do not contribute to the likelihood, because eta is set
#equal to the delta for outcome 1. #deltas for outcome 2 simply draw from the predictive distribution
      delta[i,k] ~ dnorm(md[i,k],taud[i,k])
# mean of RE distributions, with multi-arm trial correction
      md[i,k] <-  d[t[i,k]] - d[t[i,1]] + (beta[t[i,k]] - beta[t[i,1]])*step(Industry[i]-1) + sw[i,k]
# precision of RE distributions (with multi-arm trial correction)
      taud[i,k] <- tau *2*(k-1)/k
# adjustment, multi-arm RCTs
      w[i,k] <- (delta[i,k] - (d[t[i,k]] - d[t[i,1]])- (beta[t[i,k]] - beta[t[i,1]])*step(Industry[i]-1))
# cumulative adjustment for multi-arm trials
      sw[i,k] <- sum(w[i,1:k-1])/(k-1)
     }
}

#Fixed treatment effects within class
d[1]<-0       # treatment effect is zero for reference treatment / class
D[1]<-0
#Single treatment classes
for (k in 2:nt){
          d[k]<- D[classd[k]]
}

#Priors
for (k in 2:nc){  D[k] ~ dnorm(0,.0001) }
tau<-1/pow(sd,2)
sd~dunif(0,5)

for (k in 1:4){ beta[k]<-0}#No bias for control conditions
for (k in 5:nt){ beta[k]<- B}
B~dnorm(0,.0001)

# SMDs for all possible pair-wise comparisons - Treatments
for (c in 1:(nt-1)) {
          for (k in (c+1):nt) {
      smd[c,k] <- d[k] - d[c]
  }
```

```
}

# SMDs for all possible pair-wise comparisons - Classes
for (c in 1:(nc-1)) {
        for (k in (c+1):nc) {
    smdC[c,k] <- D[k] - D[c]
 }
}

# rank classes
for (k in 1:nc){
 rkC[k] <- rank(D[],k)          # assumes lower values are “good”
        #rk[k] <- nc+1-rank(D[],k)          # assumes higher values are “good”
 bestC[k] <- equals(rkC[k],1)     #calculate probability that treat k is best
 # calculate probability that class k is h-th best
 for (h in 1:nc){ probC[h,k] <- equals(rkC[k],h) }
 }

#Stop unused variables causing error message
dum[1]<-Industry[1]
dum[2]<-ITT[1]
dum[3]<-CC[1]
dum[4]<-out.type[1]
dum[5]<-class[1,1]
dum[6]<-s[1]
}                              # *** PROGRAM ENDS
```

WinBUGS code for Symptom Severity, Random Study Effect, Fixed Class Effect Model, Meta-Regression for Sample Size

```
# Normal likelihood, identity link
# Random study effects, random intervention within class effects

model{                         # *** PROGRAM STARTS
#Likelihood
for(i in 1:ndata){                 #   LOOP THROUGH STUDIES
  w[i,1] <- 0      # adjustment for multi-arm trials is zero for control arm
  delta[i,1] <- 0          # treatment effect is zero for control arm
              mu[i] ~ dnorm(0,.0001)        # vague priors for all baselines
  for (k in 1:na[i]) {          #  LOOP THROUGH ARMS
    prec[i,k] <- N[i,k]/pow(sdCFB[i,k],2)     # set precisions
                              y[i,k]<- meanCFB[i,k]
    y[i,k] ~ dnorm(theta[i,k],prec[i,k]) # normal likelihood
# model for linear predictor
                              theta[i,k]<-phi[i,k]*sd.pooled[i]
    phi[i,k] <- mu[i] + eta[i,k]
#Set relative effect for outcome 2 equal to outcome 1 for same study
#Pools outcomes from same study
                              eta[i,k]<- delta[i-out[i]+1,k]
#Deviance contribution
    dev[i,k] <- (y[i,k]-theta[i,k])*(y[i,k]-theta[i,k])*prec[i,k]
   }
```

```
#  summed residual deviance contribution for this trial/outcome
    resdev[i] <- sum(dev[i,1:na[i]])
  }
totresdev <- sum(resdev[])            #Total Residual Deviance

#RE Model
for(i in 1:ndata){                    # LOOP THROUGH STUDIES WITH ARM DATA
    for (k in 2:na[i]) {              # LOOP THROUGH ARMS
# trial-specific RE distributions
#Note delta's for outcome 2 datapoints do not contribute to the likelihood, because eta is set
#equal to the delta for outcome 1. #deltas for outcome 2 simply draw from the predictive distribution
        delta[i,k] ~ dnorm(md[i,k],taud[i,k])
# mean of RE distributions, with multi-arm trial correction
        md[i,k] <-  d[t[i,k]] - d[t[i,1]] + (beta[t[i,k]] - beta[t[i,1]])/N[i,k] + sw[i,k]
# precision of RE distributions (with multi-arm trial correction)
        taud[i,k] <- tau *2*(k-1)/k
# adjustment, multi-arm RCTs
        w[i,k] <- (delta[i,k] - (d[t[i,k]] - d[t[i,1]])- (beta[t[i,k]] - beta[t[i,1]])/N[i,k])
# cumulative adjustment for multi-arm trials
        sw[i,k] <- sum(w[i,1:k-1])/(k-1)
      }
}

#Fixed treatment effects within class
d[1]<-0       # treatment effect is zero for reference treatment / class
D[1]<-0
#Single treatment classes
for (k in 2:nt){
            d[k]<- D[classd[k]]
}

#Priors
for (k in 2:nc){  D[k] ~ dnorm(0,.0001) }
tau<-1/pow(sd,2)
sd~dunif(0,5)

for (k in 1:4){ beta[k]<-0}#No bias for control conditions
for (k in 5:nt){ beta[k]<- B}
B~dnorm(0,.0001)

# SMDs for all possible pair-wise comparisons - Treatments
for (c in 1:(nt-1)) {
            for (k in (c+1):nt) {
       smd[c,k] <- d[k] - d[c]
  }
}

# SMDs for all possible pair-wise comparisons - Classes
for (c in 1:(nc-1)) {
           for (k in (c+1):nc) {
       smdC[c,k] <- D[k] - D[c]
```

```
  }
}

# rank classes
for (k in 1:nc){
  rkC[k] <- rank(D[],k)            # assumes lower values are “good”
            #rk[k] <- nc+1-rank(D[],k)            # assumes higher values are “good”
  bestC[k] <- equals(rkC[k],1)      #calculate probability that treat k is best
  # calculate probability that class k is h-th best
  for (h in 1:nc){ probC[h,k] <- equals(rkC[k],h) }
 }

#Stop unused variables causing error message
dum[1]<-Industry[1]
dum[2]<-ITT[1]
dum[3]<-CC[1]
dum[4]<-out.type[1]
dum[5]<-class[1,1]
dum[6]<-s[1]
}                                    # *** PROGRAM ENDS
```

SUPPLEMENTARY MATERIAL S4: Included studies, study characteristics and references

#	Study ID, country, & industry funding	Population	Classes and interventions	Outcomes	Reference
1.	Abbott 2012/2018<br>New Zealand<br><br>No industry funding	N randomized: 462<br>Age in years (mean, SD): 39.2 (12.9)<br>Sex (% female): 53<br>Employment status (% FT/PT): 56<br>Marital status (% married/cohabiting): 43<br>Age in years first gambled (mean, SD): NR<br>Age in years first problem with gambling (mean, SD): NR<br>Type of gambling activity: 72% pub gaming machines; 9% club gaming machines; 7% casino gaming machines; 5% track; 3% casino tables; 2% sports betting; 2% other<br>Coexisting conditions: 63% meet AUDIT-C criteria for alcohol abuse<br>Baseline severity scale and score (mean, SD): PGSI 17.55 (5.13)	<u>Arm 1</u><br>Class: Motivational interviewing<br>Intervention: Brief motivational interviewing<br>N: 112<br>Number of sessions: 1<br><br><u>Arm 2</u><br>Class: Guided self-help<br>Intervention: Brief motivational interviewing + CBT workbook<br>N: 118<br>Number of sessions: 1<br><br><u>Arm 3</u><br>Class: Guided self-help<br>Intervention: CBT workbook with support<br>N: 116<br>Number of sessions: 5<br><br><u>Arm 4</u><br>Class: TAU<br>Intervention: Information + referral<br>N: 116<br>Number of sessions: NR<br><br>Timepoint for endpoint measurement (weeks): 13	Gambling symptom severity: PGSI<br><br>Gambling frequency: Days spent gambling in past 4 weeks	Abbott MW, Bellringer M, Vandal A, et al. Effectiveness of problem gambling brief telephone interventions: a randomised controlled trial. Gambling & Addictions Research Centre 2012. Final report (accessed June 05, 2025).<br><br>Abbott M, Hodgins DC, Bellringer M, et al. Brief telephone interventions for problem gambling: a randomized controlled trial. *Addiction 2018*; **113**: 883-95.
2.	Bouchard 2017	N randomized: 25	<u>Arm 1</u><br>Class: CBT individual (face-to-face)	Gambling symptom	Bouchard S, Robillard G,

#	Study ID, country, & industry funding	Population	Classes and interventions	Outcomes	Reference
	Canada<br><br>Data only extracted for study 3<br><br>No industry funding	Age in years (mean, SD): 47 (12.8)<br>Sex (% female): 50<br>Employment status (% FT/PT): NR<br>Marital status (% married/cohabiting): NR<br>Age in years first gambled (mean, SD): NR<br>Age in years first problem with gambling (mean, SD): NR<br>Type of gambling activity: NR<br>Coexisting conditions: NR<br>Baseline severity scale and score (mean, SD): CPGI 19.96 (3.7)	Intervention: CBT individual (face-to-face)<br>N: 14<br>Number of sessions: NR<br><br><u>Arm 2</u><br>Class: CBT individual (face-to-face)<br>Intervention: CBT individual (face-to-face)<br>N: 11<br>Number of sessions: NR<br><br>Within-class comparison (both face-to-face CBT, with or without the use of virtual reality)<br><br>Timepoint for endpoint measurement (weeks): NR	severity: CPGI and number of diagnostic criteria met (DIG: DSM-5 gambling disorder criteria)	Giroux I, et al. Using virtual reality in the treatment of gambling disorder: the development of a new tool for cognitive behavior therapy. *Frontiers in Psychiatry 2017*; **8**: 27.
3.	Boudreault 2018<br>Canada<br><br>No industry funding	N randomized: 62<br>Age in years (mean, SD): 51.5 (11.7)<br>Sex (% female): 39<br>Employment status (% FT/PT): 37<br>Marital status (% married/cohabiting): NR<br>Age in years first gambled (mean, SD): NR<br>Age in years first problem with gambling (mean, SD): NR	<u>Arm 1</u><br>Class: Guided self-help<br>Intervention: CBT workbook with support<br>N: 31<br>Number of sessions: 3<br><br><u>Arm 2</u><br>Class: Waitlist<br>Intervention: Waitlist<br>N: 31<br>Number of sessions: NA<br><br>Timepoint for endpoint measurement (weeks): 11	Gambling symptom severity: Number of diagnostic criteria met (PGDI: DSM-5 gambling disorder criteria)<br><br>Gambling frequency: Number of gambling	Boudreault C, Giroux I, Jacques C, Goulet A, Simoneau H, Ladouceur R. Efficacy of a self-help treatment for at-risk and pathological gamblers. *Journal of Gambling*

#	Study ID, country, & industry funding	Population	Classes and interventions	Outcomes	Reference
		Type of gambling activity: 87% video lottery terminals/slot machines; 13% other<br>Coexisting conditions: NR<br>Baseline severity scale and score (mean, SD): PGDI DSM-5 criteria 5.49 (2.06)		sessions in past 4 weeks	*Studies 2018*; **34**: 561-80.
4.	Boumparis 2023<br><br>Switzerland<br><br>No industry funding	N randomized: 360<br>Age in years (mean, SD): 33.4 (10.0)<br>Sex (% female): 31.7<br>Employment status (% FT/PT): NR<br>Marital status (% married/cohabiting): NR<br>Age in years first gambled (mean, SD): NR<br>Age in years first problem with gambling (mean, SD): NR<br>Type of gambling activity: NR<br>Coexisting conditions: NR<br>Baseline severity scale and score (mean, SD): PGSI 16.74 (2.74)	<u>Arm 1</u><br>Class: Self-help (with no or minimal support)<br>Intervention: Computerised CBT<br>N: 185<br>Number of sessions: NR<br><br><u>Arm 2</u><br>Class: Self-help (with no or minimal support)<br>Intervention: CBT workbook<br>N: 175<br>Number of sessions: NR<br><br>Timepoint for endpoint measurement (weeks): 8	Gambling symptom severity: PGSI and GSAS	Boumparis N, Baumgartner C, Malischnig D, Wenger A, Achab S, Khazaal Y, Keough MT, Hodgins DC, Bilevicius E, Single A, Haug S, Schaub MP. Effectiveness of a web-based self-help tool to reduce problem gambling: A randomized controlled trial. *Journal of Behavioral Addictions 2023*; **12**: 744-757.
5.	Bucker 2018 Germany	N randomized: 140	<u>Arm 1</u><br>Class: Self-help (with no or minimal support)	Gambling symptom	Bücker L, Bierbrodt J,

#	Study ID, country, & industry funding	Population	Classes and interventions	Outcomes	Reference
	Any industry funding	Age in years (mean, SD): 35.7 (10.2)<br>Sex (% female): 24<br>Employment status (% FT/PT): 69<br>Marital status (% married/cohabiting): 49<br>Age in years first gambled (mean, SD): 20.5 (8.6)<br>Age in years first problem with gambling (mean, SD): 23.8 (9.2)<br>Type of gambling activity: 100% slot machines<br>Coexisting conditions: NR<br>Baseline severity scale and score (mean, SD): SOGS 9.73 (3.18)	Intervention: Computerised CBT for depression<br>N: 71<br>Number of sessions: NR<br><br><u>Arm 2</u><br>Class: Waitlist<br>Intervention: Waitlist<br>N: 69<br>Number of sessions: NA<br><br>Timepoint for endpoint measurement (weeks): 8	severity: SOGS and PG-Y BOCS	Hand I, Wittekind C, Moritz S. Effects of a depression-focused internet intervention in slot machine gamblers: a randomized controlled trial. *PloS One 2018*; **13**: e0198859.
6.	Brazeau 2024 Canada<br><br>No industry funding	N randomized: 323<br>Age in years (mean, SD): 39.0 (11.9)<br>Sex (% female): 57<br>Employment status (% FT/PT): 62<br>Marital status (% married/cohabiting): 34<br>Age in years first gambled (mean, SD): NR<br>Age in years first problem with gambling (mean, SD): NR	<u>Arm 1</u><br>Class: Self-help (with no or minimal support)<br>Intervention: Computerised CBT<br>N: 162<br>Number of sessions: NR<br><br><u>Arm 2</u><br>Class: Guided self-help<br>Intervention: Brief motivational interviewing + computerised CBT<br>N: 161<br>Number of sessions: 1<br><br>Timepoint for endpoint measurement (weeks): 52	Gambling symptom severity: Number of diagnostic criteria met (NODS: DSM-5 gambling disorder criteria)<br><br>Gambling frequency: Hours spent	Brazeau BW, Cunningham JA, Hodgins DC. Evaluating the impact of motivational interviewing on engagement and outcomes in a web-based self-help intervention for gambling disorder: A

#	Study ID, country, & industry funding	Population	Classes and interventions	Outcomes	Reference
		Type of gambling activity: NR<br>Coexisting conditions: NR<br>Baseline severity scale and score (mean, SD): NODS 7.1 (1.9)		gambling in past 4 weeks	randomised controlled trial. *Internet Interventions 2024*; **35**: 100707.
7.	Bucker 2021<br>Germany<br><br>No industry funding	N randomized: 156<br>Age in years (mean, SD): 35.0 (11.3)<br>Sex (% female): 33<br>Employment status (% FT/PT): 65<br>Marital status (% married/cohabiting): NR<br>Age in years first gambled (mean, SD): 20.6 (13.4)<br>Age in years first problem with gambling (mean, SD): 22.3 (19.8)<br>Type of gambling activity: NR<br>Coexisting conditions: Lifetime diagnoses: 18% depression; 8% anxiety disorder; 6% alcohol/drug dependency; 5% PTSD; 1% OCD<br>Baseline severity scale and score (mean, SD): SOGS 10.34 (2.91)	<u>Arm 1</u><br>Class: Self-help (with no or minimal support)<br>Intervention: Computerised CBT<br>N: 79<br>Number of sessions: NR<br><br><u>Arm 2</u><br>Class: Waitlist<br>Intervention: Waitlist<br>N: 77<br>Number of sessions: NA<br><br>Timepoint for endpoint measurement (weeks): 8	Gambling symptom severity: SOGS and PG-Y BOCS	Bücker L, Gehlenborg J, Moritz S, Westermann S. A randomized controlled trial on a self-guided Internet-based intervention for gambling problems. *Scientific Reports 2021*; **11**: 13033.

#	Study ID, country, & industry funding	Population	Classes and interventions	Outcomes	Reference
8.	Campos 2016<br>US<br><br>No industry funding	N randomized: 87<br>Age in years (mean, SD): 44.3 (10.9)<br>Sex (% female): 24<br>Employment status (% FT/PT): 62<br>Marital status (% married/cohabiting): 23<br>Age in years first gambled (mean, SD): 20.2 (9.8)<br>Age in years first problem with gambling (mean, SD): 28.4 (13.3)<br>Type of gambling activity: NR<br>Coexisting conditions: 18% major depressive disorder; 14% generalised anxiety disorder; 10% dysthymia; 5% marijuana abuse/dependence; 3% alcohol abuse/dependence; 2% psychotic disorders<br>Baseline severity scale and score (mean, SD): NODS 6.70 (2.38)	<u>Arm 1</u><br>Class: Self-help (with no or minimal support)<br>Intervention: CBT workbook<br>N: 47<br>Number of sessions: NR<br><br><u>Arm 2</u><br>Class: Guided self-help<br>Intervention: CBT workbook with support<br>N: 40<br>Number of sessions: 5<br><br>Timepoint for endpoint measurement (weeks): 20	Gambling symptom severity: GSAS	Campos MD, Rosenthal RJ, Chen Q, Moghaddam J, Fong TW. A self-help manual for problem gamblers: The impact of minimal therapist guidance on outcome. *International Journal of Mental Health and Addiction 2016*; **14**: 579-96.
9.	Carlbring 2008<br>Sweden	N randomized: 66<br>Age in years (mean, SD): 31.9 (9.8)<br>Sex (% female): 6	<u>Arm 1</u><br>Class: Guided self-help<br>Intervention: Computerised CBT with support<br>N: 34<br>Number of sessions: 8	Gambling symptom severity: Number of diagnostic	Carlbring P, Smit F. Randomized trial of internet-delivered self-

#	Study ID, country, & industry funding	Population	Classes and interventions	Outcomes	Reference
	No industry funding	Employment status (% FT/PT): NR<br>Marital status (% married/cohabiting): NR<br>Age in years first gambled (mean, SD): 23.2 (9.5)<br>Age in years first problem with gambling (mean, SD): NR<br>Type of gambling activity: 36% video lottery terminals; 21% internet poker; 12% sports betting; 11% horses; 20% other<br>Coexisting conditions: NR<br>Baseline severity scale and score (mean, SD): NODS 8.03 (1.36)	<u>Arm 2</u><br>Class: Waitlist<br>Intervention: Waitlist<br>N: 32<br>Number of sessions: NA<br><br>Timepoint for endpoint measurement (weeks): 8	criteria met (NODS: DSM-IV pathological gambling criteria)	help with telephone support for pathological gamblers. *Journal of Consulting and Clinical Psychology 2008*; **76**: 1090.
10.	Cunningham 2009<br>Canada<br><br>Any industry funding	N randomized: NR (baseline data reported for completers only, N=45)<br>Age in years (mean, SD): 44.4 (10.9)<br>Sex (% female): 52<br>Employment status (% FT/PT): 43<br>Marital status (% married/cohabiting): 28<br>Age in years first gambled (mean, SD): NR	<u>Arm 1</u><br>Class: Self-help (with no or minimal support)<br>Intervention: Personalised feedback intervention<br>N: 22<br>Number of sessions: NR<br><br><u>Arm 2</u><br>Class: Waitlist<br>Intervention: Waitlist<br>N: 23<br>Number of sessions: NA<br><br>Timepoint for endpoint measurement (weeks): 13	Gambling symptom severity: CPGI	Cunningham JA, Hodgins DC, Toneatto T, Rai A, Cordingley J. Pilot study of a personalized feedback intervention for problem gamblers. *Behavior Therapy 2009*; **40**: 219-24.

#	Study ID, country, & industry funding	Population	Classes and interventions	Outcomes	Reference
		Age in years first problem with gambling (mean, SD): NR<br>Type of gambling activity: NR<br>Coexisting conditions: NR<br>Baseline severity scale and score (mean, SD): CPGI 14.94 (5.27)			
11.	Cunningham 2019<br>US (& Canada for 1% of N randomised)<br><br>No industry funding	N randomized: 321<br>Age in years (mean, SD): 36.5 (10.9)<br>Sex (% female): 55<br>Employment status (% FT/PT): 72<br>Marital status (% married/cohabiting): 50<br>Age in years first gambled (mean, SD): NR<br>Age in years first problem with gambling (mean, SD): NR<br>Type of gambling activity: Primary gambling concerns (each participant can select more than 1): 67% instant or scratch tickets; 54% slot machines; 51% lottery-type games; 32% casino games	Arm 1<br>Class: Self-help (with no or minimal support)<br>Intervention: Computerised personalised feedback intervention<br>N: 151<br>Number of sessions: NR<br><br>Arm 2<br>Class: No treatment<br>Intervention: No treatment<br>N: 170<br>Number of sessions: NA<br><br>Timepoint for endpoint measurement (weeks): 6	Gambling symptom severity: GSAS<br><br>Gambling frequency: Days spent gambling in past 4 weeks	Cunningham JA, Godinho A, Hodgins DC. Pilot randomized controlled trial of an online intervention for problem gamblers. *Addictive Behaviors Reports 2019*; **9**: 100175.

#	Study ID, country, & industry funding	Population	Classes and interventions	Outcomes	Reference
		Coexisting conditions: NR<br>Baseline severity scale and score (mean, SD): PGSI 11.5 (5.0)			
12.	Diskin 2009 Canada<br><br>Unclear funding source	N randomized: NR (baseline data reported for completers only, N=81)<br>Age in years (mean, SD): 45 (10.6)<br>Sex (% female): 43<br>Employment status (% FT/PT): 75<br>Marital status (% married/cohabiting): 33<br>Age in years first gambled (mean, SD): NR<br>Age in years first problem with gambling (mean, SD): NR<br>Type of gambling activity: Concerns by gambling type (several participants selected more than 1 type): 61% video lottery terminals; 33% slot machines; 19% card and dice games (either in casinos or with friends); 6% bingo; 6% lottery type games and sports select lotteries	Arm 1<br>Class: Motivational interviewing<br>Intervention: Brief motivational interviewing<br>N: 42<br>Number of sessions: 1<br><br>Arm 2<br>Class: Attention placebo<br>Intervention: Brief semi-structured interview<br>N: 39<br>Number of sessions: 1<br><br>Timepoint for endpoint measurement (weeks): 4	Gambling frequency: Days spent gambling in past 4 weeks	Diskin KM, Hodgins DC. A randomized controlled trial of a single session motivational intervention for concerned gamblers. *Behaviour Research and Therapy 2009*; **47**: 382-8.

#	Study ID, country, & industry funding	Population	Classes and interventions	Outcomes	Reference
		Coexisting conditions: NR<br>Baseline severity scale and score (mean, SD): PGSI 15.7 (5.1)			
13.	Dowling 2007<br>Australia<br><br>Unclear funding source	N randomized: 56<br>Age in years (mean, SD): 43.6 (10.4)<br>Sex (% female): 100<br>Employment status (% FT/PT): 62<br>Marital status (% married/cohabiting): 45<br>Age in years first gambled (mean, SD): NR<br>Age in years first problem with gambling (mean, SD): NR<br>Type of gambling activity: 100% gaming machines (inclusion criterion). Additional gambling types: 38% lotto; 11% phone betting; 5% scratch tickets; 4% bingo; 2% cards/casino; 2% horse/dog track betting<br>Coexisting conditions: NR<br>Baseline severity scale and score (mean, SD): DSM-IV-TR diagnostic criteria 7.02 (1.57)	Arm 1<br>Class: CBT individual (face-to-face)<br>Intervention: CBT individual (face-to-face)<br>N: 14<br>Number of sessions: 12<br><br>Arm 2<br>Class: CBT group (face-to-face)<br>Intervention: CBT group<br>N: 17<br>Number of sessions: 12<br><br>Arm 3<br>Class: Waitlist<br>Intervention: Waitlist<br>N: 25<br>Number of sessions: NA<br><br>Timepoint for endpoint measurement (weeks): 12	Gambling frequency: Gambling sessions in past week	Dowling N, Smith D, Thomas T. A comparison of individual and group cognitive-behavioural treatment for female pathological gambling. *Behaviour Research and Therapy 2007*; **45**: 2192-202.

#	Study ID, country, & industry funding	Population	Classes and interventions	Outcomes	Reference
14.	Dowling 2021<br>Australia<br><br>Any industry funding	N randomized: 207<br>Age in years (mean, SD): NR<br>Sex (% female): 35<br>Employment status (% FT/PT): 88<br>Marital status (% married/cohabiting): NR<br>Age in years first gambled (mean, SD): NR<br>Age in years first problem with gambling (mean, SD): NR<br>Type of gambling activity: Problematic gambling activities (each participant can select more than 1): 74% electronic gaming machines; 45% horse and greyhound racing; 27% sports and events betting; 16% casino table games (e.g. blackjack); 12% number games (e.g. lotteries, keno, bingo); 4% informal private games<br>Coexisting conditions: NR<br>Baseline severity scale and score (mean, SD): PGSI 17.8 (5.4)	<u>Arm 1</u><br>Class: Guided self-help<br>Intervention: Computerised CBT with support<br>N: 101<br>Number of sessions: NR<br><br><u>Arm 2</u><br>Class: Self-help (with no or minimal support)<br>Intervention: Computerised CBT<br>N: 106<br>Number of sessions: NR<br><br>Timepoint for endpoint measurement (weeks): 8	Gambling symptom severity: GSAS<br><br>Gambling frequency: Days spent gambling in past 4 weeks	Dowling NA, Merkouris SS, Rodda SN, et al. GamblingLess: a randomised trial comparing guided and unguided internet-based gambling interventions. *Journal of Clinical Medicine 2021*; **10**: 2224.

#	Study ID, country, & industry funding	Population	Classes and interventions	Outcomes	Reference
15.	Ede 2020<br>Nigeria<br><br>No industry funding	N randomized: 40<br>Age in years (mean, SD): 23.0 (3.6)<br>Sex (% female): 30<br>Employment status (% FT/PT): NR<br>Marital status (% married/cohabiting): NR<br>Age in years first gambled (mean, SD): NR<br>Age in years first problem with gambling (mean, SD): NR<br>Type of gambling activity: NR<br>Coexisting conditions: NR<br>Baseline severity scale and score (mean, SD): GSAS 36.31 (1.79)	<u>Arm 1</u><br>Class: CBT group (face-to-face)<br>Intervention: CBT group<br>N: 20<br>Number of sessions: 8<br><br><u>Arm 2</u><br>Class: Waitlist<br>Intervention: Waitlist<br>N: 20<br>Number of sessions: NA<br><br>Timepoint for endpoint measurement (weeks): 8	Gambling symptom severity: GSAS*<br><br>* made assumption that standard errors (not standard deviations) reported as implausibly small	Ede MO, Omeje JC, Ncheke DC, Agah JJ, Chinweuba NH, Amoke CV. Assessment of the effectiveness of group cognitive behavioural therapy in reducing pathological gambling. *Journal of Gambling Studies 2020*; **36**: 1325-39.
16.	Grant 2009<br>US<br><br>No industry funding	N randomized: 68<br>Age in years (mean, SD): 48.7 (12.8)<br>Sex (% female): 63<br>Employment status (% FT/PT): NR<br>Marital status (% married/cohabiting): NR<br>Age in years first gambled (mean, SD): NR<br>Age in years first problem with gambling (mean, SD): NR	<u>Arm 1</u><br>Class: CBT individual (face-to-face)<br>Intervention: Brief CBT individual (face-to-face)<br>N: 33<br>Number of sessions: 6<br><br><u>Arm 2</u><br>Class: TAU<br>Intervention: Referral to Gamblers anonymous (GA) group<br>N: 35<br>Number of sessions: NR<br><br>Timepoint for endpoint measurement (weeks): 8	Gambling symptom severity: GSAS and PG-YBOCS	Grant JE, Donahue CB, Odlaug BL, Kim SW, Miller MJ, Petry NM. Imaginal desensitisation plus motivational interviewing for pathological gambling: randomised controlled trial.

#	Study ID, country, & industry funding	Population	Classes and interventions	Outcomes	Reference
		Type of gambling activity: NR<br>Coexisting conditions: NR<br>Baseline severity scale and score (mean, SD): GSAS 29.76 (7.81)			*The British Journal of Psychiatry 2009*; **195**: 266-7.
17.	Hodgins 2001/2004<br>Canada<br><br>No industry funding	N randomized: 102<br>Age in years (mean, SD): 46 (9)<br>Sex (% female): 52<br>Employment status (% FT/PT): 64<br>Marital status (% married/cohabiting): 59<br>Age in years first gambled (mean, SD): NR<br>Age in years first problem with gambling (mean, SD): NR<br>Type of gambling activity: Major gambling problems (each participant can select more than 1): 86% video lottery terminal play (electronic slot machines); 19% casinos; 10% bingo; 4% horse racing; 4% lotteries; 3% slot machines; 3% card games; 1% speculative	Arm 1<br>Class: Self-help (with no or minimal support)<br>Intervention: CBT workbook<br>N: 35<br>Number of sessions: NR<br><br>Arm 2<br>Class: Guided self-help<br>Intervention: Brief motivational interviewing + CBT workbook<br>N: 32<br>Number of sessions: 1<br><br>Arm 3<br>Class: Waitlist<br>Intervention: Waitlist<br>N: 35<br>Number of sessions: NA<br><br>Timepoint for endpoint measurement (weeks): 4	Gambling frequency: Days spent gambling in past 4 weeks	Hodgins DC, Currie SR, el-Guebaly N. Motivational enhancement and self-help treatments for problem gambling. *Journal of Consulting and Clinical Psychology 2001*; **69**: 50.<br><br>Hodgins DC, Currie S, el-Guebaly N, Peden N. Brief motivational treatment for problem gambling: a 24-month follow-up. *Psychology of Addictive Behaviors 2004*; **18**: 293.

#	Study ID, country, & industry funding	Population	Classes and interventions	Outcomes	Reference
		investments; 1% games of skill<br>Coexisting conditions: NR<br>Baseline severity scale and score (mean, SD): SOGS 12 (3.7)			
18.	Hodgins 2009<br>Canada<br><br>No industry funding	N randomized: 314<br>Age in years (mean, SD): NR<br>Sex (% female): 55<br>Employment status (% FT/PT): 71<br>Marital status (% married/cohabiting): 46<br>Age in years first gambled (mean, SD): NR<br>Age in years first problem with gambling (mean, SD): 40.4 (11.6)<br>Type of gambling activity: Types of gambling causing problems (each participant can select more than 1): 76% video lottery terminals; 51% slot machines; 13% casinos; 6% bingo; 5% lottery type games; 5% instant or scratch tickets; 4% horse/dog racing; 2% Nevada tickets; 11% other	<u>Arm 1</u><br>Class: Guided self-help<br>Intervention: Brief motivational interviewing + CBT workbook<br>N: 83<br>Number of sessions: 1<br><br><u>Arm 2</u><br>Class: Guided self-help<br>Intervention: CBT workbook with support<br>N: 84<br>Number of sessions: 7<br><br><u>Arm 3</u><br>Class: Self-help (with no or minimal support)<br>Intervention: CBT workbook<br>N: 82<br>Number of sessions: NR<br><br><u>Arm 4</u><br>Class: Waitlist<br>Intervention: Waitlist<br>N: 65<br>Number of sessions: NA<br><br>Timepoint for endpoint measurement (weeks): 6	Gambling frequency: Days spent gambling in past 4 weeks	Hodgins DC, Currie SR, Currie G, Fick GH. Randomized trial of brief motivational treatments for pathological gamblers: More is not necessarily better. Journal of Consulting and Clinical Psychology 2009; **77**: 950.

#	Study ID, country, & industry funding	Population	Classes and interventions	Outcomes	Reference
		Coexisting conditions: 50% current major depressive disorder; 27% alcohol abuse or dependence (last 6 months); 8% lifetime diagnosis of bipolar disorder<br>Baseline severity scale and score (mean, SD): SOGS 11.1 (3.0)			
19.	Hodgins 2019<br>Canada<br><br>No industry funding	N randomized: 187<br>Age in years (mean, SD): 46.8 (12.0)<br>Sex (% female): 47<br>Employment status (% FT/PT): NR<br>Marital status (% married/cohabiting): 30<br>Age in years first gambled (mean, SD): NR<br>Age in years first problem with gambling (mean, SD): NR<br>Type of gambling activity: Types of gambling respondents engaged in within the past 3 months: 60% electronic gambling machines; 37% casino games<br>Coexisting conditions: NR	Arm 1<br>Class: Self-help (with no or minimal support)<br>Intervention: Computerised personalised feedback intervention<br>N: 92<br>Number of sessions: NR<br><br>Arm 2<br>Class: Self-help (with no or minimal support)<br>Intervention: Computerised CBT<br>N: 95<br>Number of sessions: NR<br><br>Timepoint for endpoint measurement (weeks): 13	Gambling symptom severity: Number of diagnostic criteria met (NODS: DSM-IV pathological gambling criteria)<br><br>Gambling frequency: Days spent gambling in past 4 weeks	Hodgins DC, Cunningham JA, Murray R, Hagopian S. Online self-directed interventions for gambling disorder: Randomized controlled trial. *Journal of Gambling Studies 2019*; **35**: 635-51.

#	Study ID, country, & industry funding	Population	Classes and interventions	Outcomes	Reference
		Baseline severity scale and score (mean, SD): PGSI 17.0 (5.3)			
20.	Jonas 2020 Germany<br><br>No industry funding	N randomized: 167<br>Age in years (mean, SD): 33.5 (10.6)<br>Sex (% female): 28<br>Employment status (% FT/PT): 67<br>Marital status (% married/cohabiting): NR<br>Age in years first gambled (mean, SD): NR<br>Age in years first problem with gambling (mean, SD): NR<br>Type of gambling activity: Types of gambling during the past 30 days: 67% gaming machines; 65% online gambling; 21% lotteries; 11% betting (offline); 9% other<br>Coexisting conditions: NR<br>Baseline severity scale and score (mean, SD): PGSI 16.3 (4.8)	<u>Arm 1</u><br>Class: Guided self-help<br>Intervention: Computerised counselling with support<br>N: 54<br>Number of sessions: NR<br><br><u>Arm 2</u><br>Class: Guided self-help<br>Intervention: Psychoeducational materials with email support<br>N: 56<br>Number of sessions: NR<br><br><u>Arm 3</u><br>Class: Waitlist<br>Intervention: Waitlist<br>N: 57<br>Number of sessions: NA<br><br>Timepoint for endpoint measurement (weeks): 13	Gambling symptom severity: PGSI<br>Gambling frequency: Days spent gambling in past 4 weeks	Jonas B, Leuschner F, Eiling A, Schoelen C, Soellner R, Tossmann P. Web-based intervention and email-counseling for problem gamblers: Results of a randomized controlled trial. *Journal of Gambling Studies 2020*; **36**: 1341-58.
21.	Korman 2008 Canada	N randomized: 42<br>Age in years (mean, SD): 47.6 (11.1)<br>Sex (% female): 14	<u>Arm 1</u><br>Class: Behavioural therapies individual (face-to-face)<br>Intervention: Dialectical behavior therapy (DBT), modified for anger and addiction<br>N: 20	Gambling symptom severity: PGSI	Korman L, Collins J, Littman-Sharp N, Skinner W, McMain S,

#	Study ID, country, & industry funding	Population	Classes and interventions	Outcomes	Reference
	Any industry funding	Employment status (% FT/PT): 60<br>Marital status (% married/cohabiting): 28<br>Age in years first gambled (mean, SD): NR<br>Age in years first problem with gambling (mean, SD): NR<br>Type of gambling activity: NR<br>Coexisting conditions: 45% met criteria for substance abuse. The most commonly reported substances of abuse were alcohol (84%), cannabis (42%), cocaine (32%), and opiates (26%)<br>Baseline severity scale and score (mean, SD): PGSI 15.26 (4.57)	Number of sessions: 14<br><br><u>Arm 2</u><br>Class: CBT individual (face-to-face)<br>Intervention: Brief CBT individual (face-to-face)<br>N: 22<br>Number of sessions: NR<br><br>Timepoint for endpoint measurement (weeks): 14		Mercado V. Randomized control trial of an integrated therapy for comorbid anger and gambling. *Psychotherapy Research 2008*; **18**: 454-65.
22.	LaBrie 2012 US<br><br>Any industry funding	N randomized: 315<br>Age in years (mean, SD): 46.3 (12.6)<br>Sex (% female): 42<br>Employment status (% FT/PT): NR<br>Marital status (% married/cohabiting): NR<br>Age in years first gambled (mean, SD): NR	<u>Arm 1</u><br>Class: Self-help (with no or minimal support)<br>Intervention: Psychoeducational workbook<br>N: 105<br>Number of sessions: NR<br><br><u>Arm 2</u><br>Class: Self-help (with no or minimal support)<br>Intervention: Psychoeducational workbook<br>N: 105<br>Number of sessions: NR	Gambling frequency: Days spent gambling in past 4 weeks	LaBrie RA, Peller AJ, LaPlante DA, et al. A brief self-help toolkit intervention for gambling problems: a randomized multisite trial. *American*

#	Study ID, country, & industry funding	Population	Classes and interventions	Outcomes	Reference
		Age in years first problem with gambling (mean, SD): NR<br>Type of gambling activity: NR<br>Coexisting conditions: NR<br>Baseline severity scale and score (mean, SD): NR	<u>Arm 3</u><br>Class: Waitlist<br>Intervention: Waitlist<br>N: 102<br>Number of sessions: NA<br><br>Participants in arm 2 received a phone call to introduce the psychoeducation materials but followed a guided script and only 5 mins so does not meet criteria for guided self-help<br><br>Timepoint for endpoint measurement (weeks): 4		*Journal of Orthopsychiatry 2012*; **82**: 278.
23.	Ladouceur 2001<br>Canada<br><br>Any industry funding	N randomized: 88<br>Age in years (mean, SD): 42.0 (10.2)<br>Sex (% female): 17<br>Employment status (% FT/PT): NR<br>Marital status (% married/cohabiting): NR<br>Age in years first gambled (mean, SD): NR<br>Age in years first problem with gambling (mean, SD): NR<br>Type of gambling activity: Gambling activites during previous 12 months: 93% gambling on machines (85% problems with this type of gambling); 92% lottery; 28% gambling	<u>Arm 1</u><br>Class: CBT individual (face-to-face)<br>Intervention: CBT individual (face-to-face)<br>N: 59<br>Number of sessions: ≤20<br><br><u>Arm 2</u><br>Class: Waitlist<br>Intervention: Waitlist<br>N: 29<br>Number of sessions: NA<br><br>Timepoint for endpoint measurement (weeks): 26	Gambling symptom severity: Number of diagnostic criteria met (DSM-IV pathological gambling criteria)<br><br>Gambling frequency: Gambling sessions in past week	Ladouceur R, Sylvain C, Boutin C, et al. Cognitive treatment of pathological gambling. *The Journal of Nervous and Mental Disease 2001*; **189**: 774-80.

#	Study ID, country, & industry funding	Population	Classes and interventions	Outcomes	Reference
		with cards; 15% betting on horse races; 13% sports; 13% playing blackjack; 12% bingo; 10% skill games; 10% keno<br>Coexisting conditions: NR<br>Baseline severity scale and score (mean, SD): DSM-IV diagnostic criteria 7.42 (1.60)			
24.	Ladouceur 2003<br>Canada<br><br>Unclear funding source	N randomized: NR (baseline data reported for completers only, N=59)<br>Age in years (mean, SD): 43.4 (10.5)<br>Sex (% female): 22<br>Employment status (% FT/PT): NR<br>Marital status (% married/cohabiting): NR<br>Age in years first gambled (mean, SD): NR<br>Age in years first problem with gambling (mean, SD): NR<br>Type of gambling activity: NR<br>Coexisting conditions: NR<br>Baseline severity scale and score (mean, SD):	<u>Arm 1</u><br>Class: CBT group (face-to-face)<br>Intervention: CBT group<br>N: 34<br>Number of sessions: 10<br><br><u>Arm 2</u><br>Class: Waitlist<br>Intervention: Waitlist<br>N: 25<br>Number of sessions: NA<br><br>Timepoint for endpoint measurement (weeks): 17	Gambling symptom severity: Number of diagnostic criteria met (DSM-IV pathological gambling criteria)	Ladouceur R, Sylvain C, Boutin C, Lachance S, Doucet C, Leblond J. Group therapy for pathological gamblers: a cognitive approach. *Behaviour Research and Therapy 2003*; **41**: 587-96.

#	Study ID, country, & industry funding	Population	Classes and interventions	Outcomes	Reference
		DSM-IV diagnostic criteria 7.57 (1.47)			
25.	Larimer 2012<br>US<br><br>No industry funding	N randomized: 147<br>Age in years (mean, SD): 21.2 (1.4)<br>Sex (% female): 35<br>Employment status (% FT/PT): NR<br>Marital status (% married/cohabiting): NR<br>Age in years first gambled (mean, SD): NR<br>Age in years first problem with gambling (mean, SD): NR<br>Type of gambling activity: NR<br>Coexisting conditions: NR<br>Baseline severity scale and score (mean, SD): NODS 2.27 (1.69)	Arm 1<br>Class: Motivational interviewing<br>Intervention: Brief motivational interviewing<br>N: 52<br>Number of sessions: 1<br><br>Arm 2<br>Class: CBT group (face-to-face)<br>Intervention: CBT group<br>N: 44<br>Number of sessions: 4 or 6<br><br>Arm 3<br>Class: No treatment<br>Intervention: No treatment<br>N: 51<br>Number of sessions: NA<br><br>Timepoint for endpoint measurement (weeks): 26	Gambling symptom severity: GPI and number of diagnostic criteria met (NODS DSM-IV pathological gambling criteria)	Larimer ME, Neighbors C, Lostutter TW, et al. Brief motivational feedback and cognitive behavioral interventions for prevention of disordered gambling: a randomized clinical trial. *Addiction 2012*; **107**: 1148-58.
26.	Lee 2015<br>Canada<br><br>Any industry funding	N randomized: 18<br>Age in years (mean, SD): 49.3 (SD NR)<br>Sex (% female): 34<br>Employment status (% FT/PT): 87<br>Marital status (% married/cohabiting): 100<br>Age in years first gambled (mean, SD): NR	Arm 1<br>Class: Couple interventions (face-to-face)<br>Intervention: Congruence couple therapy<br>N: 9<br>Number of sessions: 12<br><br>Arm 2<br>Class: TAU<br>Intervention: TAU<br>N: 9<br>Number of sessions: NR	Gambling symptom severity: GSAS	Lee BK, Awosoga O. Congruence couple therapy for pathological gambling: a pilot randomized controlled trial. *Journal of Gambling*

#	Study ID, country, & industry funding	Population	Classes and interventions	Outcomes	Reference
		Age in years first problem with gambling (mean, SD): NR<br>Type of gambling activity: Most common form of gambling was lottery and slots at casinos<br>Coexisting conditions: NR<br>Baseline severity scale and score (mean, SD): DSM-IV-TR diagnostic criteria 8.7 (SD NR)	Timepoint for endpoint measurement (weeks): 12		*Studies 2015*; **31**: 1047-68.
27.	Luquiens 2016<br>France<br><br>Any industry funding	N randomized: 1122<br>Age in years (mean, SD): 34.7 (10.1)<br>Sex (% female): 8<br>Employment status (% FT/PT): NR<br>Marital status (% married/cohabiting): NR<br>Age in years first gambled (mean, SD): NR<br>Age in years first problem with gambling (mean, SD): NR<br>Type of gambling activity: 100% online poker gamblers<br>Coexisting conditions: NR	<u>Arm 1</u><br>Class: Self-help (with no or minimal support)<br>Intervention: Personalised feedback intervention<br>N: 293<br>Number of sessions: NR<br><br><u>Arm 2</u><br>Class: Self-help (with no or minimal support)<br>Intervention: CBT workbook<br>N: 264<br>Number of sessions: NR<br><br><u>Arm 3</u><br>Class: Guided self-help<br>Intervention: CBT workbook with email support<br>N: 301<br>Number of sessions: NR<br><br><u>Arm 4</u><br>Class: Waitlist<br>Intervention: Waitlist	Gambling symptom severity: PGSI*<br><br>* baseline standard deviation NR, used an average of the baseline standard deviations from the 4 other studies that reported on the PGSI<br><br>Gambling frequency outcome not included in the	Luquiens A, Tanguy ML, Lagadec M, Benyamina A, Aubin HJ, Reynaud M. The efficacy of three modalities of Internet-based psychotherapy for non–treatment-seeking online problem gamblers: a randomized controlled trial. *Journal of Medical Internet*

#	Study ID, country, & industry funding	Population	Classes and interventions	Outcomes	Reference
		Baseline severity scale and score (mean, SD): PGSI 9 (4.7)	N: 264<br>Number of sessions: NA<br><br>Timepoint for endpoint measurement (weeks): 6	NMA as there was no baseline standard deviation to use for standardisation	*Research 2016*; **18**: e36.
28.	Luquiens 2025<br>France<br><br>No industry funding	N randomized: 185<br>Age in years (mean, SD): 39.2 (12.4)<br>Sex (% female): 21<br>Employment status (% FT/PT): 66<br>Marital status (% married/cohabiting): NR<br>Age in years first gambled (mean, SD): NR<br>Age in years first problem with gambling (mean, SD): NR<br>Type of gambling activity: 78% online gambling; 91% offline gambling<br>Coexisting conditions: 50% concomitant alcohol use disorder; 3.8% depression<br>Baseline severity scale and score (mean, SD): PGSI 15.2 (5.5)	<u>Arm 1</u><br>Class: Guided self-help<br>Intervention: Computerised cognitive training with support<br>N: 93<br>Number of sessions: 12<br><br><u>Arm 2</u><br>Class: Attention placebo<br>Intervention: Sham computerised cognitive training with support<br>N: 92<br>Number of sessions: 12<br><br>Timepoint for endpoint measurement (weeks): 6	Gambling symptom severity: PGSI	Luquiens A, Benyamina A, Perney P, Carré A. Computerized cognitive training for problem gambling: A randomized controlled trial (TRAIN-online). *Journal of Behavioral Addictions 2025*; **14**: 445-456.
29.	Marceaux 2011<br>US	N randomized: 38<br>Age in years (mean, SD): 46.6 (10.4)	<u>Arm 1</u><br>Class: CBT group (face-to-face)<br>Intervention: CBT group	Gambling symptom severity:	Marceaux JC, Melville CL. Twelve-step

#	Study ID, country, & industry funding	Population	Classes and interventions	Outcomes	Reference
	Unclear funding source	Sex (% female): 65<br>Employment status (% FT/PT): NR<br>Marital status (% married/cohabiting): NR<br>Age in years first gambled (mean, SD): NR<br>Age in years first problem with gambling (mean, SD): NR<br>Type of gambling activity: NR<br>Coexisting conditions: NR<br>Baseline severity scale and score (mean, SD): SOGS 13.00 (2.68)	N: 18<br>Number of sessions: 16<br><br><u>Arm 2</u><br>Class: Twelve step group programme<br>Intervention: Twelve-step facilitated group therapy<br>N: 11<br>Number of sessions: 16<br><br><u>Arm 3</u><br>Class: Waitlist<br>Intervention: Waitlist<br>N: 9<br>Number of sessions: NA<br><br>Timepoint for endpoint measurement (weeks): 8	Number of diagnostic criteria met (DSM-IV pathological gambling criteria)<br><br>Gambling frequency outcome not included in NMA as time period over which the frequency was measured was NR	facilitated versus mapping-enhanced cognitive-behavioral therapy for pathological gambling: a controlled study. *Journal of Gambling Studies 2011*; **27**: 171-90.
30.	Martens 2015<br>US<br><br>Any industry funding	N randomized: 333<br>Age in years (mean, SD): 21.9 (4.3)<br>Sex (% female): 40<br>Employment status (% FT/PT): NR<br>Marital status (% married/cohabiting): NR<br>Age in years first gambled (mean, SD): NR<br>Age in years first problem with gambling (mean, SD): NR<br>Type of gambling activity: NR	<u>Arm 1</u><br>Class: Self-help (with no or minimal support)<br>Intervention: Personalised feedback intervention<br>N: 111<br>Number of sessions: NR<br><br><u>Arm 2</u><br>Class: Self-help (with no or minimal support)<br>Intervention: Psychoeducational materials<br>N: 113<br>Number of sessions: NR<br><br><u>Arm 3</u><br>Class: No treatment<br>Intervention: No treatment<br>N: 109<br>Number of sessions: NA	Gambling symptom severity: CPGI<br><br>Gambling frequency: Days spent gambling in past 9 weeks	Martens MP, Arterberry BJ, Takamatsu SK, Masters J, Dude K. The efficacy of a personalized feedback-only intervention for at-risk college gamblers. *Journal of Consulting and Clinical Psychology 2015*; **83**: 494.

#	Study ID, country, & industry funding	Population	Classes and interventions	Outcomes	Reference
		Coexisting conditions: NR<br>Baseline severity scale and score (mean, SD): SOGS 4.77 (2.51)	Timepoint for endpoint measurement (weeks): 13		
31.	McIntosh 2016<br>Australia<br><br>Any industry funding	N randomized: 77<br>Age in years (mean, SD): 38.5 (1.7)<br>Sex (% female): 29<br>Employment status (% FT/PT): NR<br>Marital status (% married/cohabiting): NR<br>Age in years first gambled (mean, SD): NR<br>Age in years first problem with gambling (mean, SD): NR<br>Type of gambling activity: Primary gambling type: 65% electronic gaming machines; 19% horses/dogs; 16% sports<br>Coexisting conditions: NR<br>Baseline severity scale and score (mean, SD): DSM-5 gambling disorder criteria 7.51 (1.41)	<u>Arm 1</u><br>Class: CBT individual (face-to-face)<br>Intervention: Brief CBT individual (face-to-face)<br>N: 23<br>Number of sessions: 4<br><br><u>Arm 2</u><br>Class: CBT individual (face-to-face)<br>Intervention: Brief Mindfulness-based cognitive therapy (MBCT) individual<br>N: 28<br>Number of sessions: 4<br><br><u>Arm 3</u><br>Class: CBT individual (face-to-face)<br>Intervention: Brief CBT individual (face-to-face)<br>N: 26<br>Number of sessions: 7<br><br>Cross-over trial, data extracted at cross-over point (after 4 sessions; for arm 3 [referred to as TAU in paper] this is after 7 sessions)<br><br>Within-class comparison: 3 CBT arms (in arm 1 manualised CBT, in arm 2 MBCT, and in arm 3 formulation-driven)<br><br>Timepoint for endpoint measurement (weeks): 4	Gambling symptom severity: Number of diagnostic criteria met (DSM-5 gambling disorder criteria)<br><br>Gambling frequency outcome not included in NMA as time period over which the frequency was measured was NR	McIntosh CC, Crino RD, O'Neill K. Treating problem gambling samples with cognitive behavioural therapy and mindfulness-based interventions: a clinical trial. *Journal of Gambling Studies 2016*; **32**: 1305-25.
32.	Mide 2023<br>Sweden	N randomized: 60	<u>Arm 1</u><br>Class: Guided self-help	Gambling symptom	Mide M, Mattiasson J,

#	Study ID, country, & industry funding	Population	Classes and interventions	Outcomes	Reference
	Any industry funding	Age in years (mean, SD): 34.0 (9.58)<br>Sex (% female): 18.3<br>Employment status (% FT/PT): 90.0<br>Marital status (% married/cohabiting): 66.7<br>Age in years first gambled (mean, SD): NR<br>Age in years first problem with gambling (mean, SD): NR<br>Type of gambling activity: Primary gambling problem: NR<br>Coexisting conditions: NR<br>Baseline severity scale and score (mean, SD): NODS 1.4 (2.16)	Intervention: Computerised CBT with support<br>N: 30<br>Number of sessions: NR<br><br><u>Arm 2</u><br>Class: Guided self-help<br>Intervention: Computerised motivational interviewing with support<br>N: 30<br>Number of sessions: NR<br><br>Timepoint for endpoint measurement (weeks): 8	severity: NODS<br><br>Gambling frequency: Minutes spent gambling in the past week	Norlin D, Sehlin H, Rasmusson J, Ljung S, Lindskog A, Petersson J, Saavedra F, Gordh AS. Internet-delivered therapist-assisted cognitive behavioral therapy for gambling disorder: a randomized controlled trial. *Frontiers in Psychiatry 2023*; **14**: 1243826.
33.	Milton 2002 Australia<br><br>Any industry funding	N randomized: 40<br>Age in years (mean, SD): 37.6 (10.2)<br>Sex (% female): 28<br>Employment status (% FT/PT): NR<br>Marital status (% married/cohabiting): NR<br>Age in years first gambled (mean, SD): NR	<u>Arm 1</u><br>Class: CBT individual (face-to-face)<br>Intervention: Brief CBT individual (face-to-face)<br>N: 20<br>Number of sessions: 7<br><br><u>Arm 2</u><br>Class: CBT individual (face-to-face)<br>Intervention: Brief CBT individual (face-to-face)<br>N: 20<br>Number of sessions: 7<br>Within-class comparison (both CBT, intervention in arm 2 had a compliance-improving component added)	Gambling symptom severity: SOGS and number of diagnostic criteria met (SCIP DSM-IV pathological gambling criteria)	Milton S, Crino R, Hunt C, Prosser E. The effect of compliance-improving interventions on the cognitive-behavioural treatment of pathological gambling.

#	Study ID, country, & industry funding	Population	Classes and interventions	Outcomes	Reference
		Age in years first problem with gambling (mean, SD): NR<br>Type of gambling activity: Primary gambling problem: 75% poker machines; 15% off-course horse racing; 5% casino blackjack; 2.5% sports gambling; 2.5% lotto-type game, keno<br>Coexisting conditions: NR<br>Baseline severity scale and score (mean, SD): SCIP DSM-IV pathological gambling criteria 6.18 (0.69)	Timepoint for endpoint measurement (weeks): 14		*Journal of Gambling Studies 2002*; **18**: 207-29.
34.	Myrseth 2009 Norway<br><br>Unclear funding source	N randomized: 14<br>Age in years (mean, SD): 37.4 (9.5)<br>Sex (% female): 21<br>Employment status (% FT/PT): 29<br>Marital status (% married/cohabiting): 50<br>Age in years first gambled (mean, SD): NR<br>Age in years first problem with gambling (mean, SD): NR<br>Type of gambling activity: Primary	<u>Arm 1</u><br>Class: CBT group (face-to-face)<br>Intervention: CBT group<br>N: 7<br>Number of sessions: 6<br><br><u>Arm 2</u><br>Class: Waitlist<br>Intervention: Waitlist<br>N: 7<br>Number of sessions: NA<br><br>Timepoint for endpoint measurement (weeks): 8	Gambling symptom severity: Number of diagnostic criteria met (DSM-IV pathological gambling criteria)	Myrseth H, Litlerè I, Støylen IJ, Pallesen S. A controlled study of the effect of cognitive–behavioural group therapy for pathological gamblers. *Nordic Journal of Psychiatry 2009*; **63**: 22-31.

#	Study ID, country, & industry funding	Population	Classes and interventions	Outcomes	Reference
		gambling type: 71% slot machines; 29% horse racing and soccer<br>Coexisting conditions: NR<br>Baseline severity scale and score (mean, SD): DSM-IV diagnostic criteria 8.29 (1.27)			
35.	Myrseth 2011 Norway<br><br>No industry funding	N randomized: 35<br>Age in years (mean, SD): 32.8 (9.9)<br>Sex (% female): 13<br>Employment status (% FT/PT): NR<br>Marital status (% married/cohabiting): NR<br>Age in years first gambled (mean, SD): NR<br>Age in years first problem with gambling (mean, SD): NR<br>Type of gambling activity: Types of gambling played at least once a week: 33% poker/other card games; 33% sports gambling; 23% slot machines; 23% lottery/scratch cards; 20% casino games; 13% horse racing; 10% bingo; 3% Stock,	<u>Arm 1</u><br>Class: CBT individual (face-to-face)<br>Intervention: Brief CBT individual (face-to-face)<br>N: 19<br>Number of sessions: 8<br><br><u>Arm 2</u><br>Class: SSRIs<br>Intervention: Escitalopram<br>N: 16<br>Number of sessions: NA<br><br>Timepoint for endpoint measurement (weeks): 8	Gambling symptom severity: GSAS	Myrseth H, Molde H, Støylen IJ, Johnsen BH, Holsten F, Pallesen S. A pilot study of CBT versus escitalopram combined with CBT in the treatment of pathological gamblers. *International Gambling Studies 2011*; **11**: 121-41.

#	Study ID, country, & industry funding	Population	Classes and interventions	Outcomes	Reference
		options and/or commodities market<br>Coexisting conditions: Mean number of coexisting Axis I disorders: 0.75 (1.00)<br>Baseline severity scale and score (mean, SD): NODS 7.85 (1.28)			
36.	Neighbors 2015<br>US<br><br>Any industry funding	N randomized: 252<br>Age in years (mean, SD): 23.1 (5.3)<br>Sex (% female): 41<br>Employment status (% FT/PT): NR<br>Marital status (% married/cohabiting): NR<br>Age in years first gambled (mean, SD): NR<br>Age in years first problem with gambling (mean, SD): NR<br>Type of gambling activity: Gambling types (participants can select more than 1): 87% playing cards for money; 81% lotteries; 71% bingo; 67% casino gambling; 67% slots, poker, or gambling machines. Few students (<5%) endorsed daily	<u>Arm 1</u><br>Class: Self-help (with no or minimal support)<br>Intervention: Personalised feedback intervention<br>N: 124<br>Number of sessions: NR<br><br><u>Arm 2</u><br>Class: Attention placebo<br>Intervention: Attention-control (non-gambling) feedback<br>N: 128<br>Number of sessions: NR<br><br>Timepoint for endpoint measurement (weeks): 13	Gambling symptom severity: GPI	Neighbors C, Rodriguez LM, Rinker DV, et al. Efficacy of personalized normative feedback as a brief intervention for college student gambling: a randomized controlled trial. *Journal of Consulting and Clinical Psychology 2015*; **83**: 500.

#	Study ID, country, & industry funding	Population	Classes and interventions	Outcomes	Reference
		gambling on any activity. About 10% of participants reported gambling weekly or more frequently on cards (11%) and lotteries (10%)<br>Coexisting conditions: NR<br>Baseline severity scale and score (mean, SD): GPI 3.69 (6.49)			
37.	Nilsson 2020<br>Sweden<br><br>Any industry funding	N randomized: 136<br>Age in years (mean, SD): 35.6 (11.8)<br>Sex (% female): 18<br>Employment status (% FT/PT): NR<br>Marital status (% married/cohabiting): NR<br>Age in years first gambled (mean, SD): NR<br>Age in years first problem with gambling (mean, SD): NR<br>Type of gambling activity: Most problematic game: 46% online casino; 34% online betting; 5% online poker; 4% bookmaker betting; 3% slot machines; 1% horse track racing; 1%	<u>Arm 1</u><br>Class: Guided self-help<br>Intervention: Computerised CBT with support<br>N: 68<br>Number of sessions: 10<br><br><u>Arm 2</u><br>Class: Guided self-help<br>Intervention: Computerised behavioural couples therapy with support<br>N: 68<br>Number of sessions: 10<br><br>Timepoint for endpoint measurement (weeks): 12	Gambling symptom severity: Number of diagnostic criteria met (NODS DSM-IV pathological gambling criteria)	Nilsson A, Magnusson K, Carlbring P, Andersson G, Hellner C. Behavioral couples therapy versus cognitive behavioral therapy for problem gambling: a randomized controlled trial. *Addiction 2020*; **115**: 1330-42.

#	Study ID, country, & industry funding	Population	Classes and interventions	Outcomes	Reference
		trading; 7% several different<br>Coexisting conditions: NR<br>Baseline severity scale and score (mean, SD): NODS 6.5 (2.3)			
38.	Oei 2018<br>Australia<br><br>No industry funding	N randomized: 110<br>Age in years (mean, SD): 49.4 (14.1)<br>Sex (% female): 51<br>Employment status (% FT/PT): 65<br>Marital status (% married/cohabiting): 46<br>Age in years first gambled (mean, SD): NR<br>Age in years first problem with gambling (mean, SD): NR<br>Type of gambling activity: NR<br>Coexisting conditions: NR<br>Baseline severity scale and score (mean, SD): NR (baseline data only reported for 1 arm and assumed same for both arms; CPGI 16.0 (6.95))	<u>Arm 1</u><br>Class: Self-help (with no or minimal support)<br>Intervention: CBT workbook<br>N: 62<br>Number of sessions: NR<br><br><u>Arm 2</u><br>Class: Waitlist<br>Intervention: Waitlist<br>N: 48<br>Number of sessions: NA<br><br>Timepoint for endpoint measurement (weeks): 6	Gambling symptom severity: CPGI*<br><br>*baseline data only reported for 1 study arm, assumed same baseline values for both arms	Oei TP, Raylu N, Lai WW. Effectiveness of a self help cognitive behavioural treatment program for problem gamblers: a randomised controlled trial. *Journal of Gambling Studies 2018*; **34**: 581-95.
39.	Petry 2006<br>US	N randomized: 231<br>Age in years (mean, SD): 44.9 (10.9)<br>Sex (% female): 45	<u>Arm 1</u><br>Class: CBT individual (face-to-face) + TAU<br>Intervention: Brief CBT individual (face-to-face) + referral to Gamblers anonymous (GA) group	Gambling symptom severity: SOGS	Petry NM, Ammerman Y, Bohl J, et al. Cognitive-

#	Study ID, country, & industry funding	Population	Classes and interventions	Outcomes	Reference
	No industry funding	Employment status (% FT/PT): 72<br>Marital status (% married/cohabiting): 42<br>Age in years first gambled (mean, SD): 20.5 (11.1)<br>Age in years first problem with gambling (mean, SD): NR<br>Type of gambling activity: Preferred gambling type: 43% slot machines; 17% cards; 13% scratch/lottery; 9% sports; 7% animal races; 5% craps or dice game; 8% other<br>Coexisting conditions: NR<br>Baseline severity scale and score (mean, SD): DSM-IV diagnostic criteria 7.34 (1.73)	N: 84<br>Number of sessions: 8<br><br>Arm 2<br>Class: Self-help (with no or minimal support) + TAU<br>Intervention: CBT workbook + referral to Gamblers anonymous (GA) group<br>N: 84<br>Number of sessions: NR<br><br>Arm 3<br>Class: TAU<br>Intervention: Referral to Gamblers anonymous (GA) group<br>N: 63<br>Number of sessions: NR<br><br>Timepoint for endpoint measurement (weeks): 8	Gambling frequency: Days spent gambling in the past 4 weeks	behavioral therapy for pathological gamblers. *Journal of Consulting and Clinical Psychology 2006*; **74**: 555.
40.	Petry 2008<br>US<br><br>No industry funding	N randomized: 180<br>Age in years (mean, SD): 43.5 (12.8)<br>Sex (% female): 40<br>Employment status (% FT/PT): NR<br>Marital status (% married/cohabiting): 29<br>Age in years first gambled (mean, SD): NR	Arm 1<br>Class: CBT individual (face-to-face)<br>Intervention: Brief motivational interviewing + brief CBT individual (face-to-face)<br>N: 40<br>Number of sessions: 4<br><br>Arm 2<br>Class: Motivational interviewing<br>Intervention: Brief motivational interviewing<br>N: 55	Gambling symptom severity: ASI-G	Petry NM, Weinstock J, Ledgerwood DM, Morasco B. A randomized trial of brief interventions for problem and pathological gamblers.

#	Study ID, country, & industry funding	Population	Classes and interventions	Outcomes	Reference
		Age in years first problem with gambling (mean, SD): NR<br>Type of gambling activity: Preferred gambling type: 43% scratch/lottery; 18% slot machines; 17% cards; 11% sports; 8% dice; 3% other<br>Coexisting conditions: NR<br>Baseline severity scale and score (mean, SD): NODS 5.34 (3.02)	Number of sessions: 1<br><br>Arm 3<br>Class: Self-help (with no or minimal support)<br>Intervention: Personalised feedback intervention<br>N: 37<br>Number of sessions: 1<br><br>Arm 4<br>Class: No treatment<br>Intervention: No treatment<br>N: 48<br>Number of sessions: NA<br><br>Timepoint for endpoint measurement (weeks): 6		*Journal of Consulting and Clinical Psychology 2008*; **76**: 318.
41.	Petry 2009<br>US<br><br>No industry funding	N randomized: 117<br>Age in years (mean, SD): 20.4 (1.7)<br>Sex (% female): 15<br>Employment status (% FT/PT): NR<br>Marital status (% married/cohabiting): NR<br>Age in years first gambled (mean, SD): 14.5 (3.5)<br>Age in years first problem with gambling (mean, SD): NR<br>Type of gambling activity: Preferred gambling activity: 53% cards/poker; 13% sports; 10% scratch	Arm 1<br>Class: CBT individual (face-to-face)<br>Intervention: Brief motivational interviewing + brief CBT individual (face-to-face)<br>N: 21<br>Number of sessions: 4<br><br>Arm 2<br>Class: Motivational interviewing<br>Intervention: Brief motivational interviewing<br>N: 30<br>Number of sessions: 1<br><br>Arm 3<br>Class: Self-help (with no or minimal support)<br>Intervention: Personalised feedback intervention<br>N: 32<br>Number of sessions: 1<br><br>Arm 4	Gambling symptom severity: ASI-G<br><br>Gambling frequency: Days spent gambling in the past 4 weeks	Petry NM, Weinstock J, Morasco BJ, Ledgerwood DM. Brief motivational interventions for college student problem gamblers. *Addiction 2009*; **104**: 1569-78.

#	Study ID, country, & industry funding	Population	Classes and interventions	Outcomes	Reference
		tickets/lottery; 9% internet; 15% other<br>Coexisting conditions: NR<br>Baseline severity scale and score (mean, SD): NODS 3.89 (2.34)	Class: No treatment<br>Intervention: No treatment<br>N: 34<br>Number of sessions: NA<br><br>Timepoint for endpoint measurement (weeks): 6		
42.	Petry 2016<br>US<br><br>Unclear funding source	N randomized: 217<br>Age in years (mean, SD): 42.0 (10.7)<br>Sex (% female): 31<br>Employment status (% FT/PT): 15<br>Marital status (% married/cohabiting): 14<br>Age in years first gambled (mean, SD): 16.1 (6.3)<br>Age in years first problem with gambling (mean, SD): NR<br>Type of gambling activity: Preferred gambling type: 36% scratch/instant tickets; 25% card games; 22% lottery tickets; 5% sports; 5% slot machines; 5% craps or dice; 3% other<br>Coexisting conditions: 100% DSM-IV alcohol, cocaine, opioid or marijuana use disorders	<u>Arm 1</u><br>Class: CBT individual (face-to-face)<br>Intervention: Brief motivational interviewing + brief CBT individual (face-to-face)<br>N: 82<br>Number of sessions: 4<br><br><u>Arm 2</u><br>Class: Self-help (with no or minimal support)<br>Intervention: Psychoeducational materials<br>N: 69<br>Number of sessions: 1<br><br><u>Arm 3</u><br>Class: Self-help (with no or minimal support)<br>Intervention: Personalised feedback intervention<br>N: 66<br>Number of sessions: 1<br><br>Timepoint for endpoint measurement (weeks): 8	Gambling symptom severity: SOGS<br><br>Gambling frequency: Days spent gambling in the past 4 weeks	Petry NM, Rash CJ, Alessi SM. A randomized controlled trial of brief interventions for problem gambling in substance abuse treatment patients. *Journal of Consulting and Clinical Psychology 2016*; **84**: 874.

#	Study ID, country, & industry funding	Population	Classes and interventions	Outcomes	Reference
		Baseline severity scale and score (mean, SD): SOGS 7.81 (3.19)			
43.	Rodda 2018 Australia<br><br>Any industry funding	N randomized: 198<br>Age in years (mean, SD): 39.3 (12.2)<br>Sex (% female): 40<br>Employment status (% FT/PT): NR<br>Marital status (% married/cohabiting): NR<br>Age in years first gambled (mean, SD): NR<br>Age in years first problem with gambling (mean, SD): NR<br>Type of gambling activity: Type of gambling (participants can select more than 1): 71% electronic gaming machines; 45% racing (e.g. horses, dogs); 37% numbers games (e.g. lotto/bingo); 27% sports betting (e.g. football or events); 13% table games (e.g. poker, roulette); 3% private betting (e.g. cards at home)<br>Coexisting conditions: NR	<u>Arm 1</u><br>Class: Self-help (with no or minimal support) + TAU<br>Intervention: Behaviour change SMS + accessing internet mental health service<br>N: 99<br>Number of sessions: NR<br><br><u>Arm 2</u><br>Class: TAU<br>Intervention: Accessing internet mental health service<br>N: 99<br>Number of sessions: NR<br><br>Timepoint for endpoint measurement (weeks): 4	Gambling symptom severity: GSAS<br><br>Gambling frequency: Days spent gambling in the past 4 weeks	Rodda SN, Dowling NA, Knaebe B, Lubman DI. Does SMS improve gambling outcomes over and above access to other e-mental health supports? A feasibility study. *International Gambling Studies 2018*; **18**: 343-57.

#	Study ID, country, & industry funding	Population	Classes and interventions	Outcomes	Reference
		Baseline severity scale and score (mean, SD): GSAS 29.7 (7.8)			
44.	Rolvien 2024 Germany<br><br>Any industry funding	N randomized: 248<br>Age in years (mean, SD): 34.73 (10.33)<br>Sex (% female): 36.6<br>Employment status (% FT/PT): 50.6<br>Marital status (% married/cohabiting): NR<br>Age in years first gambled (mean, SD): NR<br>Age in years first problem with gambling (mean, SD): NR<br>Type of gambling activity: NR<br>Coexisting conditions: NR<br>Baseline severity scale and score (mean, SD): PGDI 17.35 (7.92)	Arm 1<br>Class: Self-help (with no or minimal support)<br>Intervention: Computerised CBT<br>N: 119<br>Number of sessions: NR<br><br>Arm 2<br>Class: Waitlist<br>Intervention: Waitlist<br>N: 124<br>Number of sessions: NA<br><br>Timepoint for endpoint measurement (weeks): 6	Gambling symptom severity: PG-YBOCS and SOGS	Rolvien L, Buddeberg L, Gehlenborg J, Borsutzky S, Moritz S. A Self-Guided Internet-Based Intervention for the Reduction of Gambling Symptoms: A Randomized Clinical Trial. *JAMA Network Open 2024*; **7**: e2417282.
45.	Smith 2015 Australia<br><br>Any industry funding	N randomized: 99<br>Age in years (mean, SD): 46.5 (13.0)<br>Sex (% female): 50<br>Employment status (% FT/PT): 53<br>Marital status (% married/cohabiting): 38<br>Age in years first gambled (mean, SD): NR	Arm 1<br>Class: CBT individual (face-to-face)<br>Intervention: CBT individual (face-to-face)<br>N: 50<br>Number of sessions: 12<br><br>Arm 2<br>Class: Behavioural therapies individual (face-to-face)<br>Intervention: Exposure therapy individual (face-to-face)<br>N: 49<br>Number of sessions: 12	Gambling symptom severity: VGS harm to self subscale	Smith DP, Battersby MW, Harvey PW, Pols RG, Ladouceur R. Cognitive versus exposure therapy for problem gambling:

#	Study ID, country, & industry funding	Population	Classes and interventions	Outcomes	Reference
		Age in years first problem with gambling (mean, SD): NR<br>Type of gambling activity: 100% electronic gaming machines (inclusion criterion)<br>Coexisting conditions: NR<br>Baseline severity scale and score (mean, SD): VGS harm to self subscale 40.67 (10.46)	Timepoint for endpoint measurement (weeks): 12		randomised controlled trial. *Behaviour Research and Therapy 2015*; **69**: 100-10.
46.	So 2020<br>Japan<br><br>No industry funding	N randomized: 254<br>Age in years (mean, SD): 36.3 (9.8)<br>Sex (% female): 21<br>Employment status (% FT/PT): 84<br>Marital status (% married/cohabiting): 14<br>Age in years first gambled (mean, SD): 19.4 (4.5)<br>Age in years first problem with gambling (mean, SD): NR<br>Type of gambling activity: Gambling preferences: 44% Pachinko; 38% slots; 9% horse races; 3% boat races; 1% bicycle	Arm 1<br>Class: Self-help (with no or minimal support)<br>Intervention: Chatbot-delivered CBT<br>N: 123<br>Number of sessions: NR<br><br>Arm 2<br>Class: No treatment<br>Intervention: No treatment<br>N: 131<br>Number of sessions: NA<br><br>Timepoint for endpoint measurement (weeks): 4	Gambling symptom severity: PGSI*<br><br>Gambling frequency: Gambling sessions in the past 4 weeks<br><br>*Paper also reports endpoint data for the GSAS but not included in the NMA as no baseline standard deviation for that scale	So R, Furukawa TA, Matsushita S, et al. Unguided chatbot-delivered cognitive behavioural intervention for problem gamblers through messaging app: a randomised controlled trial. *Journal of Gambling Studies 2020*; **36**: 1391-407.

#	Study ID, country, & industry funding	Population	Classes and interventions	Outcomes	Reference
		races; 1% speculation; 4% other<br>Coexisting conditions: NR<br>Baseline severity scale and score (mean, SD): PGSI 16.80 (4.74)			
47.	So 2024<br>Japan<br><br>No industry funding	N randomized: 139<br>Age in years (mean, SD): 42 (NR)<br>Sex (% female): 23<br>Employment status (% FT/PT): 82<br>Marital status (% married/cohabiting): 49<br>Age in years first gambled (mean, SD): NR<br>Age in years first problem with gambling (mean, SD): NR<br>Type of gambling activity: NR<br>Coexisting conditions: NR<br>Baseline severity scale and score (mean, SD): PGDI 14.6 (NR)	<u>Arm 1</u><br>Class: Guided self-help<br>Intervention: Chatbot-delivered CBT with support<br>N: 72<br>Number of sessions: NR<br><br><u>Arm 2</u><br>Class: Self-help (with no or minimal support)<br>Intervention: Chatbot-delivered CBT<br>N: 67<br>Number of sessions: NR<br><br>Timepoint for endpoint measurement (weeks): 4	Gambling symptom severity: GSAS	So R, Emura N, Okazaki K, Takeda S, Sunami T, Kitagawa K, Takebayashi Y, Furukawa TA. Guided versus unguided chatbot-delivered cognitive behavioral intervention for individuals with moderate-risk and problem gambling: A randomized controlled trial (GAMBOT2 study). *Addictive Behaviors 2024*; **149**: 107889.

#	Study ID, country, & industry funding	Population	Classes and interventions	Outcomes	Reference
48.	Thomas 2017<br>Australia<br><br>Any industry funding	N randomized: 297<br>Age in years (mean, SD): 50.5 (14.3)<br>Sex (% female): 45<br>Employment status (% FT/PT): 56<br>Marital status (% married/cohabiting): 38<br>Age in years first gambled (mean, SD): NR<br>Age in years first problem with gambling (mean, SD): NR<br>Type of gambling activity: 65% poker machines/EGMs as preferred mode of gambling<br>Coexisting conditions: NR<br>Baseline severity scale and score (mean, SD): GSAS 26.39 (8.05)	<u>Arm 1</u><br>Class: CBT individual (face-to-face)<br>Intervention: Brief CBT individual (face-to-face)<br>N: 74<br>Number of sessions: 6<br><br><u>Arm 2</u><br>Class: Behavioural therapies individual (face-to-face)<br>Intervention: Behavioural therapy individual (face-to-face)<br>N: 74<br>Number of sessions: 6<br><br><u>Arm 3</u><br>Class: Motivational interviewing<br>Intervention: Motivational interviewing<br>N: 73<br>Number of sessions: 6<br><br><u>Arm 4</u><br>Class: Counselling individual (face-to-face)<br>Intervention: Client-centred therapy (CCT)<br>N: 76<br>Number of sessions: 6<br><br>Timepoint for endpoint measurement (weeks): 12	Gambling symptom severity: GSAS<br><br>Gambling frequency: Gambling sessions in the past 4 weeks	Thomas S, Jackson A, Browning C, et al. Psychological treatments for problem gambling (PROGRESS) study: a pragmatic randomised controlled trial and qualitative study - 6 month outcomes. Victoria, Australia: Victorian Responsible Gambling Foundation, 2015.
49.	Toneatto 2009/2016<br>Canada<br><br>Any industry funding	N randomized: 99<br>Age in years (mean, SD): 47.5 (13.5)<br>Sex (% female): 26<br>Employment status (% FT/PT): 55<br>Marital status (% married/cohabiting): 38	<u>Arm 1</u><br>Class: CBT individual (face-to-face)<br>Intervention: Brief CBT individual (face-to-face)<br>N: 25<br>Number of sessions: 6<br><br><u>Arm 2</u><br>Class: Behavioural therapies individual (face-to-face)<br>Intervention: Behavioural therapy individual (face-to-face)<br>N: 24	Gambling frequency: Percentage of days spent gambling in the past 4 weeks*<br><br>* baseline data only reported for 1 study	Toneatto T, Gunaratne M. Does the treatment of cognitive distortions improve clinical outcomes for problem gambling?

#	Study ID, country, & industry funding	Population	Classes and interventions	Outcomes	Reference
		Age in years first gambled (mean, SD): 24.0 (12.3)<br>Age in years first problem with gambling (mean, SD): 35.0 (14.6)<br>Type of gambling activity: Most frequently reported gambling problems (some participants reported more than 1): 51% slot machines; 35% casino card games; 34% race track; 32% lotteries<br>Coexisting conditions: NR<br>Baseline severity scale and score (mean, SD): DSM-IV diagnostic criteria 6.5 (2.2)	Number of sessions: 6<br><br><u>Arm 3</u><br>Class: Motivational interviewing<br>Intervention: Motivational interviewing<br>N: 22<br>Number of sessions: 6<br><br><u>Arm 4</u><br>Class: Motivational interviewing<br>Intervention: Brief motivational interviewing<br>N: 28<br>Number of sessions: 1<br><br>Timepoint for endpoint measurement (weeks): 10	arm, assumed the same baseline values for all arms	*Journal of Contemporary Psychotherapy 2009*; **39**: 221-9.<br><br>Toneatto T. Single-session interventions for problem gambling may be as effective as longer treatments: results of a randomized control trial. *Addictive Behaviors 2016*; **52**: 58-65.
50.	Wittekind 2019<br>Germany<br><br>Any industry funding	N randomized: 131<br>Age in years (mean, SD): 35.2 (11.0)<br>Sex (% female): 25<br>Employment status (% FT/PT): NR<br>Marital status (% married/cohabiting): NR<br>Age in years first gambled (mean, SD): NR<br>Age in years first problem with gambling (mean, SD): NR	<u>Arm 1</u><br>Class: Self-help (with no or minimal support)<br>Intervention: Computerised attentional bias modification<br>N: 66<br>Number of sessions: NR<br><br><u>Arm 2</u><br>Class: Attention placebo<br>Intervention: Sham computerised attentional bias modification<br>N: 65<br>Number of sessions: NR<br><br>Timepoint for endpoint measurement (weeks): 8	Gambling symptom severity: PG-YBOCS	Wittekind CE, Bierbrodt J, Luedecke D, Feist A, Hand I, Moritz S. Cognitive bias modification in problem and pathological gambling using a web-based approach-avoidance task: a pilot trial.

#	Study ID, country, & industry funding	Population	Classes and interventions	Outcomes	Reference
		Type of gambling activity: 100% slot machines (inclusion criterion)<br>Coexisting conditions: NR<br>Baseline severity scale and score (mean, SD): SOGS 10.17 (3.28)			*Psychiatry Research 2019*; **272**: 171-81.
51.	Wong 2015<br>Hong Kong<br><br>Unclear funding source	N randomized: 40<br>Age in years (mean, SD): NR<br>Sex (% female): 0<br>Employment status (% FT/PT): NR<br>Marital status (% married/cohabiting): NR<br>Age in years first gambled (mean, SD): NR<br>Age in years first problem with gambling (mean, SD): NR<br>Type of gambling activity: NR<br>Coexisting conditions: NR<br>Baseline severity scale and score (mean, SD): SOGS 12.73 (4.75)	<u>Arm 1</u><br>Class: CBT group (face-to-face) + TAU<br>Intervention: CBT group + routine individual counselling<br>N: 20<br>Number of sessions: 10<br><br><u>Arm 2</u><br>Class: TAU<br>Intervention: Routine individual counselling<br>N: 20<br>Number of sessions: NR<br><br>Timepoint for endpoint measurement (weeks): 10	Gambling symptom severity: SOGS<br><br>Gambling frequency: Gambling sessions in the past 4 weeks	Wong DF, Chung CL, Wu J, Tang J, Lau P. A preliminary study of an integrated and culturally attuned cognitive behavioral group treatment for Chinese problem gamblers in Hong Kong. *Journal of Gambling Studies 2015*; **31**: 1015-27.
52.	Yokotani 2026<br>Japan	N randomized: 320<br>Age in years (mean, SD): 46.5 (11.8)<br>Sex (% female): NR	<u>Arm 1</u><br>Class: Self-help (with no or minimal support)<br>Intervention: Computerised virtual peer support<br>N: 69<br>Number of sessions: NR	Gambling symptom severity: GSAS	Yokotani K, Seki Y, Abe N, Takamura M, Yamamoto T, Takahashi H.

#	Study ID, country, & industry funding	Population	Classes and interventions	Outcomes	Reference
	No industry funding	Employment status (% FT/PT): NR<br>Marital status (% married/cohabiting): NR<br>Age in years first gambled (mean, SD): NR<br>Age in years first problem with gambling (mean, SD): NR<br>Type of gambling activity: NR<br>Coexisting conditions: NR<br>Baseline severity scale and score (mean, SD): GSAS 18.42 (9.52)	<u>Arm 2</u><br>Class: Self-help (with no or minimal support)<br>Intervention: Computerised virtual peer support<br>N: 86<br>Number of sessions: NR<br><br><u>Arm 3</u><br>Class: Self-help (with no or minimal support)<br>Intervention: Computerised virtual peer support<br>N: 75<br>Number of sessions: NR<br><br><u>Arm 4</u><br>Class: No treatment<br>Intervention: No treatment<br>N: 90<br>Number of sessions: NA<br><br>Timepoint for endpoint measurement (weeks): 8	Gambling frequency: Days spent gambling in the past week	Virtual peers reduce gambling symptoms and related problems of moderate-risk gamblers: A randomized controlled trial. *Computers in Human Behaviour 2026*; **180**: 108956

Abbreviations: ASI-G: Addiction Severity Index-Gambling subscale; AUDIT-C: Alcohol Use Disorders Identification Test; CPGI: Canadian Problem Gambling Index; DIG: Diagnostic Interview of Gambling; DSM: Diagnostic and Statistical Manual of Mental Disorders; EGM: electronic gambling machines; FT: full-time employment; GPI: Gambling Problems Index; GSAS: Gambling Symptom Assessment Scale; NA: not applicable; NODS: National Opinion Research Center DSM-IV/DSM-5 Screen For Gambling Problems; NR: not reported; OCD: obsessive-compulsive disorder; PG-Y BOCS: Pathological Gambling Adaptation of Yale-Brown Obsessive Compulsive Scale; PGDI: Pathological Gambling Diagnostic Interview-DSM-5 gambling disorder criteria; PGSI: Problem Gambling Severity Index; PT: part-time employment; PTSD: post-traumatic stress disorder; SCIP: Structured Clinical Interview for Pathological Gambling; SMS: short message service; SD: standard deviation; SOGS: South Oaks Gambling Screen; SSRIs: selective serotonin reuptake inhibitors; TAU: treatment as usual; VGS: Victorian Gambling Screen

SUPPLEMENTARY MATERIAL S5: Excluded studies, reason for exclusion and references

#	Reference	Reason for exclusion
1.	ACTRN12610000826044. 'XGAMBLE'- the effect of counselling on gambling behaviours in four New Zealand population groups. 2010. https://anzctr.org.au/ACTRN12610000826044.aspx	Publication type<br>*Trial registry protocol*
2.	ACTRN12617000646347. Comparison of two versions of psychological therapy for gambling disorder. 2017. https://anzctr.org.au/ACTRN12617000646347.aspx	Publication type<br>*Trial registry protocol*
3.	ACTRN12620000279921. A randomised control trial comparing face-to-face with online problem gambling treatment. 2020. https://anzctr.org.au/ACTRN12620000279921.aspx	Publication type<br>*Trial registry protocol*
4.	Ahluwalia, Y, Sarkar, S, Kaloiya, GS et al. A randomized controlled trial to evaluate the efficacy of inhibitory control training for aggressive behaviours among individuals with co-occurring substance use disorder and gambling behaviour. *Journal of Behavioral Addictions 2025*; **14**	Publication type<br>*Conference abstract*
5.	Amandine L, Marie-Laure T, Henri-Jean A. Online psychotherapy among problem poker gamblers: 3 years of follow up. *Journal of Behavioral Addictions 2017*; **6 (suppl 1)**: 3 (abstr).	Publication type<br>*Conference abstract*
6.	Andersson G, Rozental A, Shafran R, Carlbring P. Long-term effects of internet-supported cognitive behaviour therapy. *Expert Review of Neurotherapeutics 2018*; **18**: 21-8.	Study design<br>*Non-systematic review*
7.	Arden-Close E, Thomas S, Bush-Evans R, Hamson-Ford A, Bolat E, Hodge S, Phalp K, Wang R, McAlaney J. Feasibility and acceptability of safer gambling interventions (social norms and goal setting) delivered via text message for those at low-to-moderate risk of gambling harm: a four-arm randomised controlled feasibility study. *BMJ Open 2025*; **15**: e104784.	Population<br>*Indicated prevention - 'at risk' gamblers*
8.	Armstrong T, Rockloff M, Browne M, Blaszczynski A. Training gamblers to re-think their gambling choices: how contextual analytical thinking may be useful in promoting safer gambling. *Journal of Behavioral Addictions 2020*; **9**: 766-84.	Outcome data could not be included in the NMA<br>*Only reported time spent gambling*
9.	Auer MM, Griffiths MD. The use of personalized behavioral feedback for online gamblers: an empirical study. *Frontiers in Psychology 2015*; **6**: 1406.	Study design<br>*Non-randomised trial*
10.	Auer MM, Griffiths MD. Personalized behavioral feedback for online gamblers: a real world empirical study. *Frontiers in Psychology 2016*; **7**: 1875.	Study design<br>*Non-randomised trial*
11.	Augner C, Vlasak T, Aichhorn W, Barth A. Psychological online interventions for problem gambling and gambling disorder–a meta-analytic approach. *Journal of Psychiatric Research 2022*; **151**: 86-94.	Systematic review<br>*Individual studies included in this review have been assessed for eligibility*
12.	Battersby M. Cognitive versus exposure therapy for problem gambling: a randomised controlled trial. *Australian and New Zealand Journal of Psychiatry 2015*; **49 (suppl 1)**: 76 (abstr).	Publication type<br>*Conference abstract*

13.	Bellringer ME, Palmer du Preez K, Vandal AC, et al. Effectiveness of face-to-face gambling interventions: a randomised controlled trial. 2021. Auckland University of Technology, Gambling and Addictions Research Centre. Ministry of Health NZ (accessed May 19, 2025).	Data cannot be extracted<br>*Only results of statistical analysis reported*
14.	Bellringer ME, Palmer du Preez K, Vandal AC et al. Effectiveness of face-to-face gambling interventions: Two years later. 2022. Auckland University of Technology, Gambling and Addictions Research Centre. Ministry of Health NZ (accessed June 6, 2026).	Data cannot be extracted<br>*Only results of statistical analysis reported*
15.	Bergeron PY, Giroux I, Chrétien M, Bouchard S. Exposure therapy for gambling disorder: systematic review and meta-analysis. *Current Addiction Reports 2022*; **9**: 179-94.	Systematic review<br>*Individual studies included in this review have been assessed for eligibility*
16.	Bergeron PY, Giroux I, Chrétien M, Bouchard S. Exposure therapy for gambling disorder: systematic review and meta-analysis. *Current Addiction Reports 2022*; **9**: 179-94.	Duplicate
17.	Bouchard AE, Dickler M, Renauld E, et al. Concurrent transcranial direct current stimulation and resting-state functional magnetic resonance imaging in patients with gambling disorder. *Brain Connectivity 2021*; **11**: 815-21.	Intervention<br>*Intervention is not psychological or psychosocial*
18.	Boughton RR, Jindani F, Turner NE. Group treatment for women gamblers using web, teleconference and workbook: effectiveness pilot. *International Journal of Mental Health and Addiction 2016*; **14**: 1074-95.	Study design<br>*Non-randomised trial*
19.	Boughton R, Jindani F, Turner NE. Closing a treatment gap in Ontario: pilot of a tutorial workbook for women gamblers. *Journal of Gambling Issues 2017*; **36**: 199-231.	Study design<br>*Non-randomised trial*
20.	Boumparis N, Haug S, Abend S, Billieux J, Riper H, Schaub MP. Internet-based interventions for behavioral addictions: a systematic review. *Journal of Behavioral Addictions 2022*; **11**: 620-42.	Systematic review<br>*Individual studies included in this review have been assessed for eligibility*
21.	Bui V, Baumgartner C, Bilevicius E, Single A, Vedelago L, Morris V, Kempe T, Schaub MP, Stewart SH, MacKillop J, Hodgins DC, Wardell JD, O'Connor R, Read J, Hadjistavropoulos HD, Sundström C, Dermody S, Kim AH, Keough MT. Efficacy of a novel online integrated treatment for problem gambling and tobacco smoking: Results of a randomized controlled trial. *Journal of Behavioral Addictions 2023*; **12**: 168-181.	Intervention<br>*Integrated gambling and tobacco addiction*
22.	Canale N, Vieno A, Griffiths MD, et al. The efficacy of a web-based gambling intervention program for high school students: a preliminary randomized study. *Computers in Human Behavior 2016*; **55**: 946-54.	Population<br>*Participants aged<18 years*
23.	Carlbring P, Jonsson J, Josephson H, Forsberg L. Motivational interviewing versus cognitive behavioral group therapy in the treatment of problem and pathological gambling: a randomized controlled trial. *Cognitive Behaviour Therapy 2010*; **39**: 92-103.	Data cannot be extracted<br>*Data not presented per arm*

24.	Casey LM, Oei TP, Raylu N, et al. Internet-based delivery of cognitive behaviour therapy compared to monitoring, feedback and support for problem gambling: a randomised controlled trial. *Journal of Gambling Studies 2017*; **33**: 993-1010.	Data cannot be extracted<br>*Ns not reported ('Available data at post ranged between n = 18–27 for I-CBT, n = 18–30 for I-MFS, and n = 38–44 for Waitlist')*
25.	Chebli JL, Blaszczynski A, Gainsbury SM. Internet-based interventions for addictive behaviours: a systematic review. *Journal of Gambling Studies 2016*; **32**: 1279-304.	Systematic review<br>*Individual studies included in this review have been assessed for eligibility*
26.	Choi YS. Effectiveness of psychosocial rehabilitation program for the pathologic gamblers in Korea. *International Journal of Neuropsychology 2010*; **13 (suppl 1)**: 48 (abstr).	Publication type<br>*Conference abstract*
27.	Christensen DR, Dowling NA, Jackson AC, et al. A proof of concept for using brief dialectical behavior therapy as a treatment for problem gambling. *Behaviour Change 2013*; **30**: 117-37.	Study design<br>*Non-randomised trial*
28.	Clarke C, Skokauskas N. CBT for adolescent pathological gambling–lessons from adult research. *Irish Journal of Psychological Medicine 2009*; **26**: 140-6.	Systematic review<br>*Individual studies included in this review have been assessed for eligibility*
29.	Cowlishaw S, Merkouris S, Dowling N, Anderson C, Jackson A, Thomas S. Psychological therapies for pathological and problem gambling. *Cochrane Database of Systematic Reviews 2012*; **11**: CD008937.	Systematic review<br>*Individual studies included in this review have been assessed for eligibility*
30.	Cunningham JA, Hodgins DC, Toneatto T, Murphy M. A randomized controlled trial of a personalized feedback intervention for problem gamblers. *PLoS One 2012*; **7**: e31586.	Population<br>*Population below threshold for problem gambling*
31.	Cunningham JA, Hodgins DC, Sharman S, Walker H, Schell C. Pilot Randomised Trial of a Brief Online Personalised Feedback Intervention for the UK Context Designed To Prevent, Reduce, and Address Gambling Harm. *Journal of Gambling Studies 2025*; **41**: 977-991.	Population<br>*Indicated prevention – population below threshold for problem gambling*
32.	Danielsson AK, Eriksson AK, Allebeck P. Technology-based support via telephone or web: a systematic review of the effects on smoking, alcohol use and gambling. *Addictive Behaviors 2014*; **39**: 1846-68.	Systematic review<br>*Individual studies included in this review have been assessed for eligibility*
33.	Di Nicola M, De Crescenzo F, D'Alò GL, et al. Pharmacological and psychosocial treatment of adults with gambling disorder: a meta-review. *Journal of Addiction Medicine 2020*; **14**: e15-23.	Systematic review<br>*Individual studies included in this review have been assessed for eligibility*
34.	Dickinson P, Gerling K, Wilson L, Parke A. Virtual reality as a platform for research in gambling behaviour. *Computers in Human Behavior 2020*; **107**: 106293.	Study design<br>*Non-randomised trial*

35.	DiClemente CC, Corno CM, Graydon MM, Wiprovnick AE, Knoblach DJ. Motivational interviewing, enhancement, and brief interventions over the last decade: a review of reviews of efficacy and effectiveness. *Psychology of Addictive Behaviors 2017*; **31**: 862.	Systematic review<br>*Individual studies included in this review have been assessed for eligibility*
36.	Doiron JP, Nicki RM. Prevention of pathological gambling: a randomized controlled trial. *Cognitive Behaviour Therapy 2007*; **36**: 74-84.	Population<br>*Indicated prevention - 'at risk' gamblers, but those with pathological/problem gambling excluded*
37.	Donati MA, Iozzi A, Fusi G, et al. A cognitive group therapy for patients in case of gambling disorder: the utility of the dual-process model. *Journal of Behavioral Addictions 2022*; **11 (suppl 1)**: 259 (abstr).	Study design<br>*Non-randomised trial*
38.	Dowling N, Smith D, Thomas T. Efficacy of a cognitive-behavioural approach in the treatment of female pathological gambling. *Australian Journal of Psychology 2004*; **56 (suppl 1)**: 179 (abstr).	Publication type<br>*Conference abstract*
39.	Dowling N, Smith D, Thomas T. Treatment of female pathological gambling: the efficacy of a cognitive-behavioural approach. *Journal of Gambling Studies 2006*; **22**: 355-72.	Data cannot be extracted<br>*Ns not reported (data for all treated participants combined for intervention group [including those initially on waitlist], also only includes participants who selected abstinence as treatment goal)*
40.	Dowling N, Jackson AC, Thomas SA. Behavioral interventions in the treatment of pathological gambling: a review of activity scheduling and desensitization. International *Journal of Behavioral Consultation and Therapy 2008*; **4**: 172-87.	Study design<br>*Non-systematic review*
41.	Dowling N, Merkouris S, Rodda S, et al. Development and evaluation of an online gambling self-directed program: effective integration into existing services. 2018. Victorian Responsible Gambling Foundation. https://responsiblegambling.vic.gov.au/resources/publications/development-and-evaluation-of-an-online gambling-self-directed-program-effective-integration into-existing-services-348/ (accessed May 19, 2025)	Study design<br>*Non-randomised trial*
42.	Dowling NA, Merkouris SS, Greenwood CJ, Youssef GJ, Thomas AC, Hawker CO, Lubman DI, Rodda SN. GamblingLess: In-The-Moment: a mixed-methods acceptability and engagement evaluation of a gambling just-in-time adaptive intervention. *Addiction Science & Clinical Practice 2025*; **20**: 80.	Data cannot be extracted<br>*No relevant outcomes reported*
43.	DRKS00011569. Evaluation of the effectiveness of "Check dein Spiel" (CDS), an internet-based intervention for pathological gambling. 2017. https://drks.de/search/en/trial/DRKS00011569	Publication type<br>*Trial registry protocol*

44.	DRKS00024840. Further development of the Internet-based self-help program "Restart" for individuals with gambling problems: a randomized controlled trial. 2021. https://drks.de/search/en/trial/DRKS00024840	Publication type<br>*Trial registry protocol*
45.	DRKS00013888. Efficacy of a depression-focused internet intervention in slot machine gamblers: a randomized controlled trial. 2018. https://drks.de/search/en/trial/DRKS00013888/details	Publication type<br>*Trial registry protocol*
46.	Echeburúa E, Gómez M, Freixa M. Cognitive-behavioural treatment of pathological gambling in individuals with chronic schizophrenia: a pilot study. *Behaviour Research and Therapy 2011*; **49**: 808-14.	Study design<br>*Non-randomised trial*
47.	Eriksen JW, Fiskaali A, Zachariae R, Wellnitz KB, Oernboel E, Stenbro AW, Marcussen T, Petersen MW. Psychological intervention for gambling disorder: A systematic review and meta-analysis. *Journal of Behavioral Addictions 2023*; **12**: 613-630.	Systematic review<br>*Individual studies included in this review have been assessed for eligibility*
48.	Fiskaali A, Stenbro AW, Marcussen T, Rask MT. Preventive interventions and harm reduction in online and electronic gambling: a systematic review. *Journal of Gambling Studies 2023*; **39**: 883-911.	Systematic review<br>*Individual studies included in this review have been assessed for eligibility*
49.	Forman DP, Boughter JR, McAfee NW, Ginley MK, Whelan JP, Pfund RA. Motivational-interviewing-informed interventions for problem gambling and gambling disorder: A systematic review and meta-analysis. Psychol Addict Behav. 2025 Jun;39(4):365-374.	Systematic review<br>*Individual studies included in this review have been assessed for eligibility*
50.	Free BL, Halle Smith E, Ginley MK, Whelan JP, Pfund RA. Does cognitive-behavioral treatment affect putative mechanisms of change among individuals with problem gambling? A systematic review and exploratory meta-analysis. *Addictive Behaviors 2024*; **158**: 108110	Systematic review<br>*Individual studies included in this review have been assessed for eligibility*
51.	Ghosh A, Dhawan L, Basu D. Treating gambling disorder (GD): a biofeedback based exposure therapy. *Indian Journal of Psychiatry 2015*; **57**: S139-S140 (abstr).	Publication type<br>*Conference abstract*
52.	Giroux I, Faucher-Gravel A, St-Hilaire A, Boudreault C, Jacques C, Bouchard S. Gambling exposure in virtual reality and modification of urge to gamble. *Cyberpsychology, Behavior, and Social Networking 2013*; **16**: 224-31.	Study design<br>*Non-randomised trial*
53.	Gooding P, Tarrier N. A systematic review and meta-analysis of cognitive-behavioural interventions to reduce problem gambling: hedging our bets? *Behaviour Research and Therapy 2009*; **47**: 592-607.	Systematic review<br>*Individual studies included in this review have been assessed for eligibility*
54.	Gordh, A.S. Internet-delivered therapist-assisted cognitive behavioral therapy for gambling disorder: A randomized controlled trial. *Journal of Behavioral Addictions 2024*; 13(supplement1): 70	Duplicate of Mide 2023

55.	Goslar M, Leibetseder M, Muench HM, Hofmann SG, Laireiter AR. Efficacy of face-to-face versus self-guided treatments for disordered gambling: a meta-analysis. *Journal of Behavioral Addictions 2017*; **6**: 142-62.	Systematic review<br>*Individual studies included in this review have been assessed for eligibility*
56.	Grande-Gosende A, Lopez-Nunez C, Garcia-Fernandez G, Derevensky J, Fernandez-Hermida JR. Systematic review of preventive programs for reducing problem gambling behaviors among young adults. *Journal of Gambling Studies 2020*; **36**: 1-22.	Systematic review<br>*Individual studies included in this review have been assessed for eligibility*
57.	Grant JE, Donahue CB, Odlaug BL, Kim SW. A 6-month follow-up of imaginal desensitization plus motivational interviewing in the treatment of pathological gambling. *Annals of Clinical Psychiatry 2011*; **23**: 3-10.	Data cannot be extracted<br>*Follow-up data for Grant 2009 but only for intervention arm (so no comparator) as waitlist arm received intervention after endpoint*
58.	Harris N, Mazmanian D. Cognitive behavioural group therapy for problem gamblers who gamble over the internet: a controlled study. *Journal of Gambling Issues 2016*; **33**: 170-88.	Study design<br>*Quasi-randomised study (participants assigned sequentially according to the order of study entry)*
59.	Higueruela-Ahijado M, López-Espuela F, Caro-Alonso PÁ, Novo A, Rodríguez-Martín B. Efficacy of cognitive-behavioral therapy in improving the quality of life of people with compulsive gambling, a systematic review. *Archives of Psychiatric Nursing 2023*; **43**: 127-142.	Systematic review<br>*Individual studies included in this review have been assessed for eligibility*
60.	Hodgins DC, Toneatto T, Makarchuk K, Skinner W, Vincent S. Minimal treatment approaches for concerned significant others of problem gamblers: a randomized controlled trial. *Journal of Gambling Studies 2007*; **23**: 215-30.	Population<br>*Concerned significant others rather than the people who participate in harmful gambling*
61.	Humphreys G, Evans R, Makin H, Cooke R, Jones A. Identification of behavior change techniques from successful web-based interventions targeting alcohol consumption, binge eating, and gambling: systematic review. *Journal of Medical Internet Research 2021*; **23**: e22694.	Systematic review<br>*Individual studies included in this review have been assessed for eligibility*
62.	Hutchison P, Cox S, Frings D. Helping you helps me: giving and receiving social support in recovery groups for problem gamblers. *Group Dynamics: Theory, Research, and Practice 2018*; **22**: 187-199.	Study design<br>*Non-randomised trial*
63.	Iriki A. Our group therapy session has helped the people with gambling disorder. *Journal of Behavioral Addictions 2019*; **8**: 120-121.	Study design<br>*Non-randomised trial*
64.	ISRCTN92322614. Is motivational interviewing and/or cognitive behavioural group therapy an effective treatment for pathological gambling? 2008. https://doi.org/10.1186/ISRCTN92322614	Publication type<br>*Trial registry protocol*
65.	ISRCTN38692394. A randomized controlled trial of an Internet-based psychological treatment for disordered gambling. 2019. https://doi.org/10.1186/ISRCTN38692394	Publication type<br>*Trial registry protocol*

66.	Jimenez-Murcia S, Aymamí N, Gómez-Peña M, et al. Does exposure and response prevention improve the results of group cognitive-behavioural therapy for male slot machine pathological gamblers? *British Journal of Clinical Psychology 2012*; **51**: 54-71.	Study design<br>*Non-randomised trial*
67.	Jiménez-Murcia S, Tremblay J, Stinchfield R, et al. The involvement of a concerned significant other in gambling disorder treatment outcome. *Journal of Gambling Studies 2017*; **33**: 937-53.	Study design<br>*Non-randomised trial*
68.	Jonsson J, Hodgins DC, Munck I, Carlbring P. Reaching out to big losers: a randomized controlled trial of brief motivational contact providing gambling expenditure feedback. *Psychology of Addictive Behaviors 2019*; **33**: 179-189.<br>Jonsson J, Hodgins DC, Munck I, Carlbring P. Reaching out to big losers leads to sustained reductions in gambling over 1 year: a randomized controlled trial of brief motivational contact. *Addiction 2020*; **115**: 1522-31.	Data cannot be extracted<br>*Unclear if endpoint data is ITT or completer analysis and follow-up data are not reported by arm*
69.	Josephson H, Carlbring P, Forsberg L, Rosendahl I. People with gambling disorder and risky alcohol habits benefit more from motivational interviewing than from cognitive behavioral group therapy. *PeerJ 2016*; **4**: e1899.	Data cannot be extracted<br>*Secondary analysis of Carlbring et al. 2010 (excluded see above), and data presented for subgroups with no Ns*
70.	Kotter R, Kraeplin A, Pittig A, Buehringer G. A systematic review of land-based self-exclusion programs: demographics, gambling behavior, gambling problems, mental symptoms, and mental health. *Journal of Gambling Studies 2019*; **35**: 367-94.	Intervention<br>*Population-level intervention*
71.	Ladouceur R, Sylvain C, Gosselin P. Self-exclusion program: a longitudinal evaluation study. *Journal of Gambling Studies 2007*; **23**: 85-94.	Intervention<br>*Population-level intervention*
72.	Lee BK, Ofori Dei SM, Brown MM, Awosoga OA, Shi Y, Greenshaw AJ. Congruence couple therapy for alcohol use and gambling disorders with comorbidities (part I): outcomes from a randomized controlled trial. *Family Process 2023*; **62**: 124-59.	Population<br>*68% of sample have alcohol use disorder (AUD) only, and data not reported separately for those that have gambling disorder (alone [5%] or together with AUD [27%])*
73.	Lee BK, Ofori Dei SM, Brown MM, Awosoga OA, Shi Y, Greenshaw AJ. Congruence couple therapy for alcohol use and gambling disorders with comorbidities (part I): outcomes from a randomized controlled trial. *Family Process 2023*; **62**: 124-59.	Duplicate
74.	Lee BK, Ofori Dei SM, Isik E. Congruence couple therapy for alcohol use and gambling disorders with comorbidities (part II): targeted areas and mechanisms of change. *Family Process 2023*; **62**: 534-56.	Population<br>*68% of sample have alcohol use disorder (AUD) only, and data not reported separately for those that have gambling disorder (alone [5%] or together with AUD [27%])*

75.	Lee BK, Ofori Dei SM, Isik E. Congruence couple therapy for alcohol use and gambling disorders with comorbidities (part II): targeted areas and mechanisms of change. *Family Process 2023*; **62**: 534-56.	Duplicate
76.	Lee BK, Ofori Dei SM. Changes in Work Status, Couple Adjustment, and Recovery Capital: Secondary Analysis of Data From a Congruence Couple Therapy Randomized Controlled Trial. *Substance Abuse 2022*; **16**: 11782218221088875.	Population<br>*68% of sample have alcohol use disorder (AUD) only, and data not reported separately for those that have gambling disorder (alone [5%] or together with AUD [27%])*
77.	Lee EJ. Effects of Positive Psychology Counselling Combined with Auricular Acupressure on Resilience and Gambling Withdrawal Symptoms. *Issues in Mental Health Nursing 2025*; **46**: 1025-1033.	Data cannot be extracted for outcomes of interest
78.	Leibetseder M, Laireiter AR, Vierhauser M, Hittenberger B. Efficacy and effectiveness of psychological and psycho-pharmacological treatments in pathological gambling–a meta-analysis. *Sucht 2011*; **57**: 275-85.	Non-English language paper
79.	Leurent M, Ducasse D, Courtet P, Olié E. Efficacy of 12-step mutual-help groups other than Alcoholics Anonymous: a systematic review and meta-analysis. *European Archives of Psychiatry & Clinical Neuroscience 2024*; **274**: 375-422.	Systematic review<br>*Individual studies included in this review have been assessed for eligibility*
80.	Linardatou C, Parios A, Varvogli L, Chrousos G, Darviri C. An 8-week stress management program in pathological gamblers: a pilot randomized controlled trial. *Journal of Psychiatric Research 2014*; **56**: 137-43.	Intervention<br>*Intervention not targeted at gambling and no relevant gambling outcomes*
81.	Luquiens A, Lagadec M, Tanguy ML, Aubin HJ, Reynaud M. OR-57: Efficacy of online psychotherapies in poker gambling disorder: an online randomized clinical trial. *Journal of Behavioral Addictions 2015*; **4 (suppl 1)**: 27-9 (abstr).	Publication type<br>*Conference abstract*
82.	Luquiens A, Lagadec M, Tanguy M, Reynaud M. Efficacy of online psychotherapies in poker gambling disorder: an online randomized clinical trial. *European Psychiatry 2015*; **30 (suppl 1)**: 1 (abstr).	Publication type<br>*Conference abstract*
83.	Luquiens A, Lagadec M, Tanguy M, Reynaud M. Efficacy of online psychotherapies in poker gambling disorder: an online randomized clinical trial. *European Psychiatry 2015*; **30 (suppl 1)**: 1 (abstr).	Duplicate
84.	Luquiens A. Big data to track and treat? Proposing online therapy to problem gamblers: a randomized clinical trial. *European Psychiatry 2018*; **48 (suppl)**: 19-20 (abstr).	Publication type<br>*Conference abstract*
85.	Luquiens A, Dugravot A, Panjo H, Benyamina A, Gaïffas S, Bacry E. Self-exclusion among online poker gamblers: Effects on expenditure in time and money as compared to matched controls. *International Journal of Environmental Research and Public Health 2019*; **16**: 4399.	Intervention<br>*Population-level intervention*

86.	Luquiens, A., Benyamina, A., Perney, P. et al. Cognitive control training in problem gambling: An online randomized controlled trial. *Journal of Behavioral Addictions 2024*; **13(supplement1)**: 69	Publication type<br>*Conference abstract*
87.	Makani R, Pradhan B, Shah U, Parikh T. Role of repetitive transcranial magnetic stimulation (rTMS) in treatment of addiction and related disorders: a systematic review. *Current Drug Abuse Reviews 2017*; **10**: 31-43.	Intervention<br>*Intervention is not psychological or psychosocial*
88.	Marchica L, Derevensky JL. Examining personalized feedback interventions for gambling disorders: a systematic review. *Journal of Behavioral Addictions 2016*; **5**: 1-10.	Systematic review<br>*Individual studies included in this review have been assessed for eligibility*
89.	Martinotti G, Pettorruso M. Brain stimulation and gambling disorder: new therapeutic perspectives. *Journal of Behavioral Addictions 2018*; **7 (suppl 1)**: 111 (abstr).	Intervention<br>*Intervention is not psychological or psychosocial*
90.	Martinotti G, Lupi M, Montemitro C, et al. Transcranial direct current stimulation reduces craving in substance use disorders: a double-blind, placebo-controlled study. *The Journal of ECT 2019*; **35**: 207-11.	Intervention<br>*Intervention is not psychological or psychosocial*
91.	Matsuzaki T, Matsushita S, Nishimura K, Furuno S, Okada H, Higuchi S. Effectiveness of CBT-based outpatient treatment program for gambling disorder: multi-study site randomized control trial in Japan. *Journal of Behavioral Addictions 2019*; **8 (suppl 1)**: 68 (abstr).	Publication type<br>*Conference abstract*
92.	Maynard BR, Wilson AN, Labuzienski E, Whiting SW. Mindfulness-based approaches in the treatment of disordered gambling: a systematic review and meta-analysis. *Research on Social Work Practice 2018*; **28**: 348-62.	Systematic review<br>*Individual studies included in this review have been assessed for eligibility*
93.	McCormick AV, Cohen IM, Davies G. Differential effects of formal and informal gambling on symptoms of problem gambling during voluntary self-exclusion. *Journal of Gambling Studies 2018*; **34**: 1013-31.	Intervention<br>*Population-level intervention*
94.	McMahon N, Thomson K, Kaner E, Bambra C. Effects of prevention and harm reduction interventions on gambling behaviours and gambling related harm: an umbrella review. *Addictive Behaviors 2019*; **90**: 380-8.	Study design<br>*Systematic review of reviews (umbrella review)*
95.	Melville CL, Davis CS, Matzenbacher DL, Clayborne J. Node-link-mapping-enhanced group treatment for pathological gambling. *Addictive Behaviors 2004*; **29**: 73-87.	Data cannot be extracted<br>*No measure of variance reported*
96.	Mena-Moreno T, Munguía L, Granero R, Lucas I, Fernández-Aranda F, Gómez-Peña M, Moragas L, Verdejo-García A, Menchón JM, Jiménez-Murcia S. e-Estesia: A Serious Game for Reducing Arousal, Improving Emotional Regulation and Increasing Wellbeing in Individuals with Gambling Disorder. *Journal of Clinical Medicine 2022*; **11**: 6798.	Intervention<br>*Add-on intervention not primarily targeted at gambling but, more generally, at impulsivity*

97.	Morefield K, Walker C, Smith D, Harvey P, Dunn K, Battersby M. An inpatient treatment program for people with gambling problems: synopsis and early outcomes. *International Journal of Mental Health and Addiction 2014*; **12**: 367-79.	Study design<br>*Non-randomised trial*
98.	Muller K, Koch A, Dickenhorst U, et al. OR-61: Effects of inpatient treatment of pathological gamblers: first results of a multicenter follow-up study. *Journal of Behavioral Addictions 2015*; **4 (suppl 1)**: 29-30 (abstr).	Publication type<br>*Conference abstract*
99.	Myrseth H, Brunborg GS, Eidem M, Pallesen S. Description and pre-post evaluation of a telephone and Internet based treatment programme for pathological gambling in Norway: a pilot study. *International Gambling Studies 2013*; **13**: 205-20.	Study design<br>*Non-randomised trial*
100.	Nagar M, Gueta K, van Mierlo T. Reducing gambling harm through digital self-help intervention: a pilot study in mild to moderate gambling disorder. *Harm Reduction Journal 2026*; **23**: 43.	Data cannot be extracted<br>*Only results of statistical analysis reported*
101.	Naish KR, Vedelago L, MacKillop J, Amlung M. Effects of neuromodulation on cognitive performance in individuals exhibiting addictive behaviors: a systematic review. *Drug and Alcohol Dependence 2018*; **192**: 338-51.	Intervention<br>*Intervention is not psychological or psychosocial*
102.	Najavits LM, Ledgerwood DM, Afifi TO. A Randomized Controlled Trial for Gambling Disorder and PTSD: Seeking Safety and CBT. *Journal of Gambling Studies 2023*; **39**: 1865-1884.	Data cannot be extracted<br>*No relevant data reported*
103.	NCT00158314. Cognitive behavioral therapy for treatment of pathological gambling. 2005. https://clinicaltrials.gov/study/NCT00158314	Publication type<br>*Trial registry protocol*
104.	NCT00345527. A randomized control trial examining two treatments for problem gambling. 2006. https://clinicaltrials.gov/study/NCT00345527	Publication type<br>*Trial registry protocol*
105.	NCT00578357. A personalized feedback intervention for problem gamblers. 2007. https://clinicaltrials.gov/study/NCT00578357	Publication type<br>*Trial registry protocol*
106.	NCT00685048. Brief therapies for problem gambling substance abusers. 2008. https://clinicaltrials.gov/study/NCT00685048	Publication type<br>*Trial registry protocol*
107.	NCT01154712. Deep low-frequency repetitive transcranial magnetic stimulation for cessation of pathological gambling. 2010. https://clinicaltrials.gov/study/NCT01154712	Intervention<br>*Intervention is not psychological or psychosocial*
108.	NCT02953899. Contingency management as an adjunct treatment for rural and remote disordered gamblers. 2016. https://clinicaltrials.gov/study/NCT02953899	Publication type<br>*Trial registry protocol*
109.	NCT03287583. SBIRT intervention for gambling behaviors. 2017. https://clinicaltrials.gov/study/NCT03287583	Publication type<br>*Trial registry protocol*
110.	NCT03464838. Effects of transcranial direct current stimulation (tDCS) in gambling disorder. 2018. https://clinicaltrials.gov/study/NCT03464838	Intervention<br>*Intervention is not psychological or psychosocial*
111.	NCT03497247. Mindfulness-based cognitive-behavioral therapy for gambling disorder. 2018. https://clinicaltrials.gov/study/NCT03497247	Publication type<br>*Trial registry protocol*

112.	Nilsson A, Magnusson K, Carlbring P, Andersson G, Gumpert CH. The development of an internet-based treatment for problem gamblers and concerned significant others: a pilot randomized controlled trial. *Journal of Gambling Studies 2018*; **34**: 539-59.	Data cannot be extracted<br>*Ns not reported*
113.	Oakley-Browne M, Adams P, Mobberley P. Interventions for pathological gambling. *Cochrane Database of Systematic Reviews 2000*; **1**: CD001521.	Systematic review<br>*Individual studies included in this review have been assessed for eligibility*
114.	Oei TP, Raylu N, Casey LM. Effectiveness of group and individual formats of a combined motivational interviewing and cognitive behavioral treatment program for problem gambling: a randomized controlled trial. *Behavioural and Cognitive Psychotherapy 2010*; **38**: 233-8.	Data cannot be extracted<br>*Ns not reported*
115.	Pallesen S, Mitsem M, Kvale G, Johnsen BH, Molde H. Outcome of psychological treatments of pathological gambling: a review and meta-analysis. *Addiction 2005*; **100**: 1412-22.	Systematic review<br>*Individual studies included in this review have been assessed for eligibility*
116.	Park JJ, King DL, Wilkinson-Meyers L, Rodda SN. Content and effectiveness of web-based treatments for online behavioral addictions: systematic review. *JMIR Mental Health 2022*; **9**: e36662.	Systematic review<br>*Individual studies included in this review have been assessed for eligibility*
117.	Peter SC, Brett EI, Suda MT, et al. A meta-analysis of brief personalized feedback interventions for problematic gambling. *Journal of Gambling Studies 2019*; **35**: 447-64.	Systematic review<br>*Individual studies included in this review have been assessed for eligibility*
118.	Petry NM, Ginley MK, Rash CJ. A systematic review of treatments for problem gambling. *Psychology of Addictive Behaviors 2017*; **31**: 951.	Systematic review<br>*Individual studies included in this review have been assessed for eligibility*
119.	Pfund RA, Peter SC, Whelan JP, Meyers AW, Ginley MK, Relyea G. Is more better? A meta-analysis of dose and efficacy in face-to-face psychological treatments for problem and disordered gambling. *Psychology of Addictive Behaviors 2020*; **34**: 557.	Systematic review<br>*Individual studies included in this review have been assessed for eligibility*
120.	Pfund RA, Forman DP, Whalen SK, Zech JM, Ginley MK, Peter SC, McAfee NW, Whelan JP. Effect of cognitive-behavioral techniques for problem gambling and gambling disorder: A systematic review and meta-analysis. *Addiction 2023*; **118**: 1661-1674.	Systematic review<br>*Individual studies included in this review have been assessed for eligibility*
121.	Pfund RA, Ginley MK, Kim HS, Boness CL, Horn TL, Whelan JP. Cognitive-behavioral treatment for gambling harm: Umbrella review and meta-analysis. *Clinical Psychology Review 2023*; **105**: 102336.	Systematic review

		Individual studies included in this review have been assessed for eligibility
122.	Pfund RA, King SA, Forman DP, Zech JM, Ginley MK, Peter SC, McAfee NW, Witkiewitz K, Whelan JP. Effects of cognitive behavioral techniques for gambling on recovery defined by gambling, psychological functioning, and quality of life: A systematic review and meta-analysis. *Psychology of Addictive Behaviors 2023*; **37**: 936-945.	Systematic review<br>*Individual studies included in this review have been assessed for eligibility*
123.	Pietsch B, Arnaud N, Lochbühler K, Rossa M, Kraus L, Gomes de Matos E, Grahlher K, Thomasius R, Hanewinkel R, Morgenstern M. Effects of an App-Based Intervention Program to Reduce Substance Use, Gambling, and Digital Media Use in Adolescents and Young Adults: A Multicenter, Cluster-Randomized Controlled Trial in Vocational Schools in Germany. *International Journal of Environmental Research & Public Health 2023*; **20**: 1970.	Population<br>*Population were not adults aged 18 years or over.*
124.	Protasio MIB, da Silva JPL, Arias-Carrión O, Nardi AE, Machado S, Cruz MS. Repetitive transcranial magnetic stimulation to treat substance use disorders and compulsive behavior. *CNS & Neurological Disorders-Drug Targets 2015*; **14**: 331-40.	Intervention<br>*Intervention is not psychological or psychosocial*
125.	Quilty LC, Wardell JD, Thiruchselvam T, Keough MT, Hendershot CS. Brief interventions for problem gambling: a meta-analysis. *PloS One 2019*; **14**: e0214502.	Systematic review<br>*Individual studies included in this review have been assessed for eligibility*
126.	Ranta J, Bellringer M, Garrett N, Abbott M. Can a brief telephone intervention for problem gambling help to reduce co-existing depression? A three-year prospective study in New Zealand. *Journal of Gambling Studies 2019*; **35**: 617-33.	Study design<br>*Non-randomised trial*
127.	Ribeiro EO, Afonso NH, Morgado P. Non-pharmacological treatment of gambling disorder: a systematic review of randomized controlled trials. *BMC Psychiatry 2021*; **21**: 1-5.	Systematic review<br>*Individual studies included in this review have been assessed for eligibility*
128.	Robillard G, Bouchard S, Boutin C, Vezina J. Effectiveness of a revised virtual reality program for pathological gambling. *Journal of Cyber Therapy and Rehabilitation 2016*; **9 (suppl 1)**: 55 (abstr).	Publication type<br>*Conference abstract*
129.	Rodda SN. A systematic review of internet delivered interventions for gambling: prevention, harm reduction and early intervention. *Journal of Gambling Studies 2022*; **38**: 967-91.	Systematic review<br>*Individual studies included in this review have been assessed for eligibility*
130.	Rodda SN. A systematic review of internet delivered interventions for gambling: prevention, harm reduction and early intervention. *Journal of Gambling Studies 2022*; **38**: 967-91.	Duplicate
131.	Rodda SN, Merkouris SS, Greenwood CJ, Thomas AC, Lubman DI, Dowling NA. Acceptability of the content and functionality of a just-in-time adaptive intervention for gambling problems: a mixed-methods evaluation of gambling habit hacker. *Addiction Science & Clinical Practice 2025*; **20**: 46.	Study design<br>*Not an RCT*

132.	Rogers R. Psychological treatments improve pathological gambling in the short and long term. *Evidence-based Mental Health 2006*; **9**: 44.	Publication type<br>*Non-primary publication (summary of systematic review already assessed)*
133.	Sagoe D, Griffiths MD, Erevik EK, et al. Internet-based treatment of gambling problems: a systematic review and meta-analysis of randomized controlled trials. *Journal of Behavioral Addictions 2021*; **10**: 546-65.	Systematic review<br>*Individual studies included in this review have been assessed for eligibility*
134.	Sauvaget A, Bulteau S, Guilleux A, et al. Both active and sham low-frequency rTMS single sessions over the right DLPFC decrease cue-induced cravings among pathological gamblers seeking treatment: a randomized, double-blind, sham-controlled crossover trial. *Journal of Behavioral Addictions 2018*; **7**: 126-36.	Intervention<br>*Intervention is not psychological or psychosocial*
135.	Savron G, De Luca R, Pitti P. Group therapy with pathological gamblers: results after 6, 12 and 18 months of treatment. *Rivista Di Psichiatria 2007*; **42**: 189-204.	Non-English language paper
136.	Saxton J, Rodda SN, Booth N, Merkouris SS, Dowling NA. The efficacy of personalized normative feedback interventions across addictions: a systematic review and meta-analysis. *PloS One 2021*; **16**: e0248262.	Systematic review<br>*Individual studies included in this review have been assessed for eligibility*
137.	Schuler A, Ferentzy P, Turner NE, et al. Gamblers anonymous as a recovery pathway: a scoping review. *Journal of Gambling Studies 2016*; **32**: 1261-78.	Study design<br>*Scoping review including randomised and non-randomised studies*
138.	Segawa T, Baudry T, Bourla A, et al. Virtual reality (VR) in assessment and treatment of addictive disorders: a systematic review. *Frontiers in Neuroscience 2020*; **13**: 1409.	Systematic review<br>*Individual studies included in this review have been assessed for eligibility*
139.	Shahrajabian F, Hasani J, Hodgins D, Griffiths MD. Emotional Working Memory Training Treatment for Young Adult Problem Online Sports Bettors: A Preliminary Randomized Controlled Trial. *Journal of Gambling Studies 2024*; **40**: 2209-2235.	Data cannot be extracted<br>*No relevant outcomes reported*
140.	Smith DP, Dunn KI, Harvey PW, Battersby MW, Pols RG. Assessing randomised clinical trials of cognitive and exposure therapies for gambling disorders: a systematic review. *Behaviour Change 2013*; **30**: 139-58.	Systematic review<br>*Individual studies included in this review have been assessed for eligibility*
141.	Smith DP, Fairweather-Schmidt AK, Harvey PW, Battersby MW. How does routinely delivered cognitive–behavioural therapy for gambling disorder compare to “gold standard” clinical trial? *Clinical Psychology & Psychotherapy 2018*; **25**: 302-10.	Study design<br>*Comparing intervention effectiveness data from RCT to intervention outcomes from routine intervention delivery in standard care*

142.	Smith EH, Free BL, Ginley MK, Whelan JP, Pfund RA. Personalized Feedback Interventions for Indicated Prevention of Gambling Disorder: A Systematic Review and Meta-Analysis. *Journal of Gambling Studies 2025*; **41**: 1563-1581.	Systematic review<br>*Individual studies included in this review have been assessed for eligibility*
143.	Soyata AZ, Aksu S, Woods AJ, İşçen P, Saçar KT, Karamürsel S. Effect of transcranial direct current stimulation on decision making and cognitive flexibility in gambling disorder. *European Archives of Psychiatry and Clinical Neuroscience 2019*; **269**: 275-84.	Intervention<br>*Intervention is not psychological or psychosocial*
144.	Tabri N, Wood RTA, Wohl MJA. Effects of personalized and normative feedback via the Positive Play Quiz on responsible gambling intention, self-efficacy and behavior: A randomized controlled trial. *Addiction 2025*; **120**: 1016-1027.	Data cannot be extracted<br>*No relevant outcomes reported*
145.	Tannous A, Demetrovics Z, Chhabra B, Logemann A, Czakó A, Griffiths MD, Szabo A. Mindfulness-based interventions for gambling disorder: A systematic review. *Journal of Behavioral Addictions 2026*; **15**: 115-134.	Systematic review<br>*Individual studies included in this review have been assessed for eligibility*
146.	Tavares H, Zilberman ML, el-Guebaly N. Are there cognitive and behavioural approaches specific to the treatment of pathological gambling? *The Canadian Journal of Psychiatry 2003*; **48**: 22-7.	Study design<br>*Mapping review to identify types of CBT approaches specific to gambling treatment*
147.	Toneatto T, Ladoceur R. Treatment of pathological gambling: a critical review of the literature. *Psychology of Addictive Behaviors 2003*; **17**: 284.	Study design<br>*Non-systematic review*
148.	Toneatto T, Dragonetti R. Effectiveness of community-based treatment for problem gambling: a quasi-experimental evaluation of cognitive-behavioral vs. twelve-step therapy. *American Journal on Addictions 2008*; **17**: 298-303.	Study design<br>*Non-randomised trial*
149.	Toneatto T, Pillai S, Courtice EL. Mindfulness-enhanced cognitive behavior therapy for problem gambling: a controlled pilot study. *International Journal of Mental Health and Addiction 2014*; **12**: 197-205.	Study design<br>*Non-randomised trial*
150.	Torrance J, Russell AMT, Heath C, Newall P. The effect of a brief intervention video on gambling advertising resistance: Results of a randomized, on-line experimental study. *Addiction 2025*; **120**:1028-1039.	Data cannot be extracted<br>*No relevant data reported*
151.	Tremblay J, Dufour M, Bertrand K, Saint-Jacques M, Ferland F, Blanchette-Martin N, Savard AC, Côté M, Berbiche D, Beaulieu M. Efficacy of a randomized controlled trial of integrative couple treatment for pathological gambling (ICT-PG): 10-month follow-up. *Journal of Consulting & Clinical Psychology 2023*; **91**: 221-233.	Data cannot be extracted<br>*Couples therapy added on to specialised treatment in addiction treatment centres and no clear endpoint data for the experimental intervention*
152.	Tse S, Campbell L, Rossen F, et al. Face-to-face and telephone counseling for problem gambling: a pragmatic multisite randomized study. *Research on Social Work Practice 2013*; **23**: 57-65.	Data cannot be extracted<br>*Results not reported by arm*

153.	von Hammerstein C, Benyamina A, Luquiens A. Long-term efficacy of an optimized online gambling self-exclusion procedure with extended suspension of commercial solicitations: a randomized controlled trial. *Harm Reduction Journal 2025*; **22**: 204.	Intervention<br>*Population-level intervention*
154.	Wall H, Magnusson K, Hellner C, Andersson G, Jayaram-Lindström N, Rosendahl I. The evaluation of a brief ICBT program with therapist support for individuals with gambling problems in the context of a gambling helpline: a randomized pilot trial. *Pilot & Feasibility Studies 2023*; **9**: 26.	Data cannot be extracted<br>*Ns not reported*
155.	Williams JM, Heath C, Leightley D, Murphy D, Dymond S. Smartphone app-delivered Acceptance and Commitment Therapy (ACT) for post-traumatic stress disorder and gambling harm in veterans: A pilot feasibility study. *Journal of Contextual Behavioral Science 2025*; **38**: 100956	Study design<br>*Not an RCT*
156.	Wulfert, E, Broussard J, Wemm SE, Glanton Holzhauer C, Rubonis AV. The Challenge of Retaining Gamblers in Treatment: Comparing Cognitive Motivational Behavior Therapy and CBT. *Cognitive and Behavioral Practice 2025*; in press: https://doi.org/10.1016/j.cbpra.2025.08.004	Study design<br>*Assignment not truly random, every second woman was assigned to the opposite group to which the previous woman had been randomised*
157.	Wulfert E, Wemm SE, Broussard JD. Betting on change: An analysis of cognitive motivational behavior therapy versus referral to gamblers anonymous for gambling disorder. *Psychology of Addictive Behaviors 2025*; **39**: 375-388.	Study design<br>*Assignment not truly random, every second woman was assigned to the opposite group to which the previous woman had been randomised*
158.	Yakovenko I, Quigley L, Hemmelgarn BR, Hodgins DC, Ronksley P. The efficacy of motivational interviewing for disordered gambling: systematic review and meta-analysis. *Addictive Behaviors 2015*; **43**: 72-82.	Systematic review<br>*Individual studies included in this review have been assessed for eligibility*
159.	Yakovenko I, Hodgins DC. Effectiveness of a voluntary casino self-exclusion online self-management program. *Internet Interventions 2021*; **23**: 100354.	Intervention<br>*Population-level intervention*
160.	Yokomitsu, K, Inoue, K, Kamimura, E et al. Effectiveness of Internet-based personalized normative feedback for help-seeking behaviors among individuals experiencing problem gambling. *Journal of Behavioral Addictions 225*; **14**	Publication type<br>*Conference abstract*
161.	Yokomitsu K, Inoue K, Kamimura E, Matsushita S, So R. Effectiveness of Internet-Based Personalized Normative Feedback Among Individuals Experiencing Problem Gambling: Randomized Controlled Trial. *Journal of Gambling Studies 2025*; **41**: 615-641.	Study design<br>*Assignment not truly random, alternate allocation*
162.	Zack M, Cho SS, Parlee J, Jacobs M, Li C, Boileau I, Strafella A. Effects of high frequency repeated transcranial magnetic stimulation and continuous theta burst stimulation on gambling reinforcement, delay discounting, and stroop interference in men with pathological gambling. *Brain Stimulation 2016*; **9**: 867-75.	Intervention<br>*Intervention is not psychological or psychosocial*

163.	Zhuang XY, Wong DF, Ng TK, Jackson AC, Dowling NA, Lo HH. Evaluating the effectiveness of an integrated cognitive-behavioural intervention (CBI) model for male problem gamblers in Hong Kong: a matched-pair comparison design. *Journal of Gambling Studies 2018*; **34**: 969-85.	Study design<br>*Non-randomised trial*
164.	Zucchella C, Mantovani E, Federico A, Lugoboni F, Tamburin S. Non-invasive brain stimulation for gambling disorder: a systematic review. *Frontiers in Neuroscience 2020*; **14**: 729.	Intervention<br>*Intervention is not psychological or psychosocial*

SUPPLEMENTARY MATERIAL S6: Risk of bias of individual studies included in the NMAs

Figure S1. Risk of bias assessment using the Cochrane risk of bias tool presented as percentages across all included studies

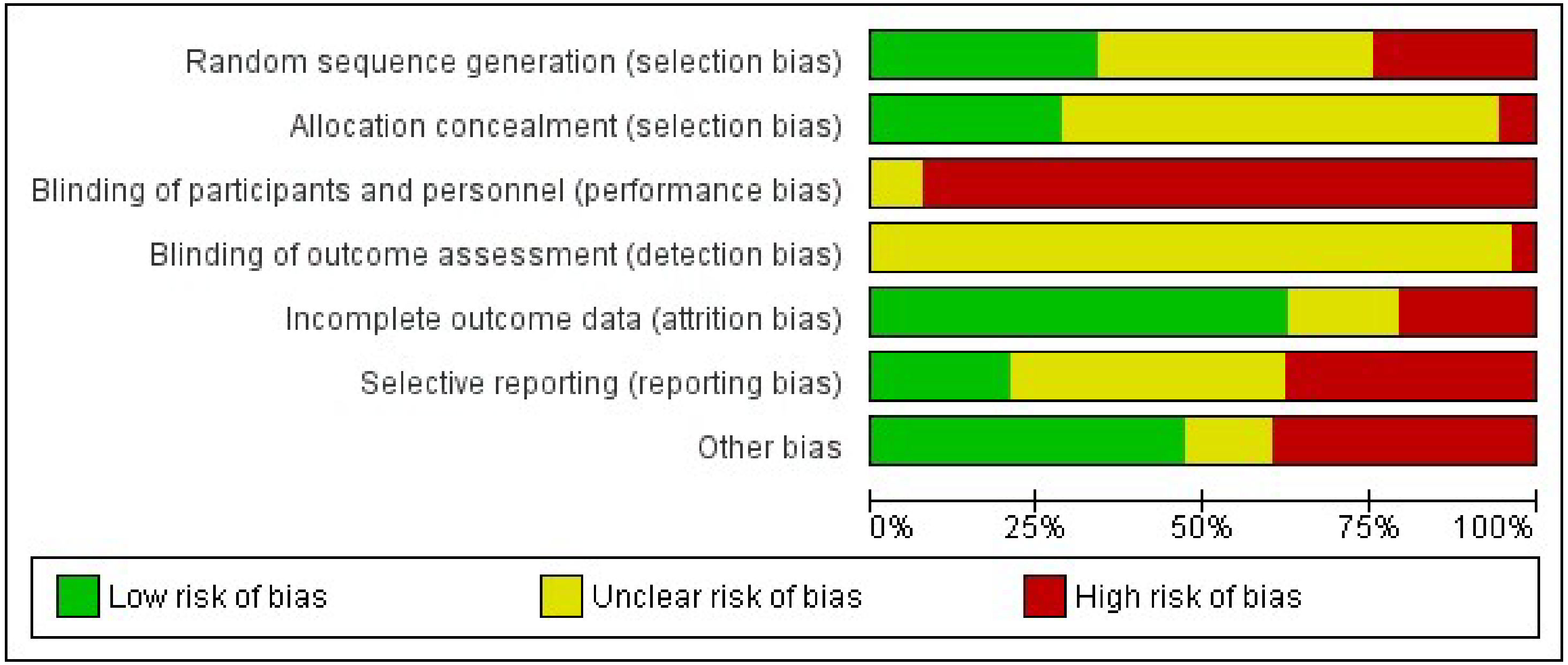

Figure S2. Risk of bias assessment using the Cochrane risk of bias tool for each included study

Study	Random sequence generation (selection bias)	Allocation concealment (selection bias)	Blinding of participants and personnel (performance bias)	Blinding of outcome assessment (detection bias)	Incomplete outcome data (attrition bias)	Selective reporting (reporting bias)	Other bias
Abbott 2012/2018	-	?	-	?	+	+	+
Bouchard 2017	?	?	-	?	+	-	+
Boudreault 2018	?	?	-	?	+	-	+
Boumparis 2023	+	?	-	?	+	+	+
Brazeau 2024	+	?	-	-	+	?	+
Bucker 2018	-	+	-	?	-	?	-
Bucker 2021	+	+	-	?	-	+	+
Campos 2016	-	?	-	?	-	-	+
Carlbring 2008	?	+	-	?	+	-	+
Cunningham 2009	-	?	-	?	+	?	-
Cunningham 2012	?	?	-	?	+	-	-
Cunningham 2019	-	?	-	?	+	+	+
Diskin 2009	+	?	?	?	+	-	?
Dowling 2007	?	?	-	?	+	-	?
Dowling 2021	+	+	-	?	-	-	-
Ede 2020	+	?	-	?	+	?	+
Grant 2009	?	?	-	?	+	-	+
Hodgins 2001/2004	?	?	-	?	+	-	+
Hodgins 2009	+	?	-	?	+	-	+
Hodgins 2019	+	?	-	?	+	+	+
Jonas 2020	-	+	-	?	-	+	+
Korman 2008	+	?	-	?	+	?	-
LaBrie 2012	-	-	-	?	+	?	-
Ladouceur 2001	?	?	-	?	-	?	-
Ladouceur 2003	?	?	-	?	?	-	?
Larimer 2012	?	?	-	?	?	?	+
Lee 2015	?	?	-	?	+	-	-
Luquiens 2016	?	?	-	?	-	-	-
Luquiens 2025	?	?	?	-	?	+	+
Marceaux 2011	?	?	-	?	?	-	?
Martens 2015	-	?	-	?	+	?	-
McIntosh 2016	-	?	-	?	+	?	-
Mide 2023	+	+	-	?	+	+	-
Milton 2002	+	-	-	?	-	?	-
Myrseth 2009	-	?	-	?	?	?	?
Myrseth 2011	+	?	-	?	-	?	+
Neighbors 2015	-	+	?	?	+	?	-
Nilsson 2020	?	+	-	?	?	?	-
Oei 2018	?	?	-	?	-	-	+
Petry 2006	-	?	-	?	+	-	+
Petry 2008	?	+	-	?	+	?	+
Petry 2009	?	+	-	?	+	-	+
Petry 2016	+	?	-	?	+	-	?
Rodda 2018	?	+	-	?	-	?	-
Rolvien 2024	+	?	-	?	+	+	-
Smith 2015	?	+	-	?	?	-	-
So 2020	?	+	-	?	+	?	+
So 2024	+	+	-	?	+	+	+
Thomas 2017	+	+	-	?	+	+	-
Toneatto 2009/2016	+	?	-	?	+	?	-
Wittekind 2019	-	-	?	?	?	?	-
Wong 2015	?	?	-	?	?	?	?
Yokotani 2026	+	?	-	?	+	?	+

SUPPLEMENTARY MATERIAL S7: Model fit statistics

Model fit statistics for the base case analysis for the outcome symptom severity, using the full dataset

Model	Total residual deviance	Dbar	pD	DIC	Between-study SD (95% CrI)	Between-intervention SD (95% CrI)	Regression coefficient (95%CrI)
**RE study, FE class**	132.5	202.4	110.6	313	0.57 (0.44 to 0.73)	-	-
**FE study, FE class**	425.6	495.5	66.9	562.5	-	-	-
**RE study, RE class**	132.6	202.5	111.1	313.6	0.56 (0.43 to 0.73)	0.13 (0.00 to 0.37)	-
**Inconsistency Model [RE study, FE class]**	141.5	211.4	100.6	312	0.24 (0.15 to 0.36)	-	-
**Meta-Regression, sample size**	131.7	201.6	111	313	0.59 (0.46 to 0.75)	-	-6.10 (-26.66 to 14.92)
**Meta-regression, industry funding**	131.4	201.3	111.2	312.5	0.59 (0.46 to 0.75)	-	-0.33 (-1.10 to 0.44)

Models differed in their specification of effects structure on study effects and treatment class effects. 54 studies with 128 study arms.
CrI: credible intervals; FE: fixed effect; RE: random effects; SD: standard deviation

Dev-dev plot for gambling severity, showing the posterior mean deviance for each data-point for the inconsistency model plotted against the NMA model (random study, fixed class effect model)

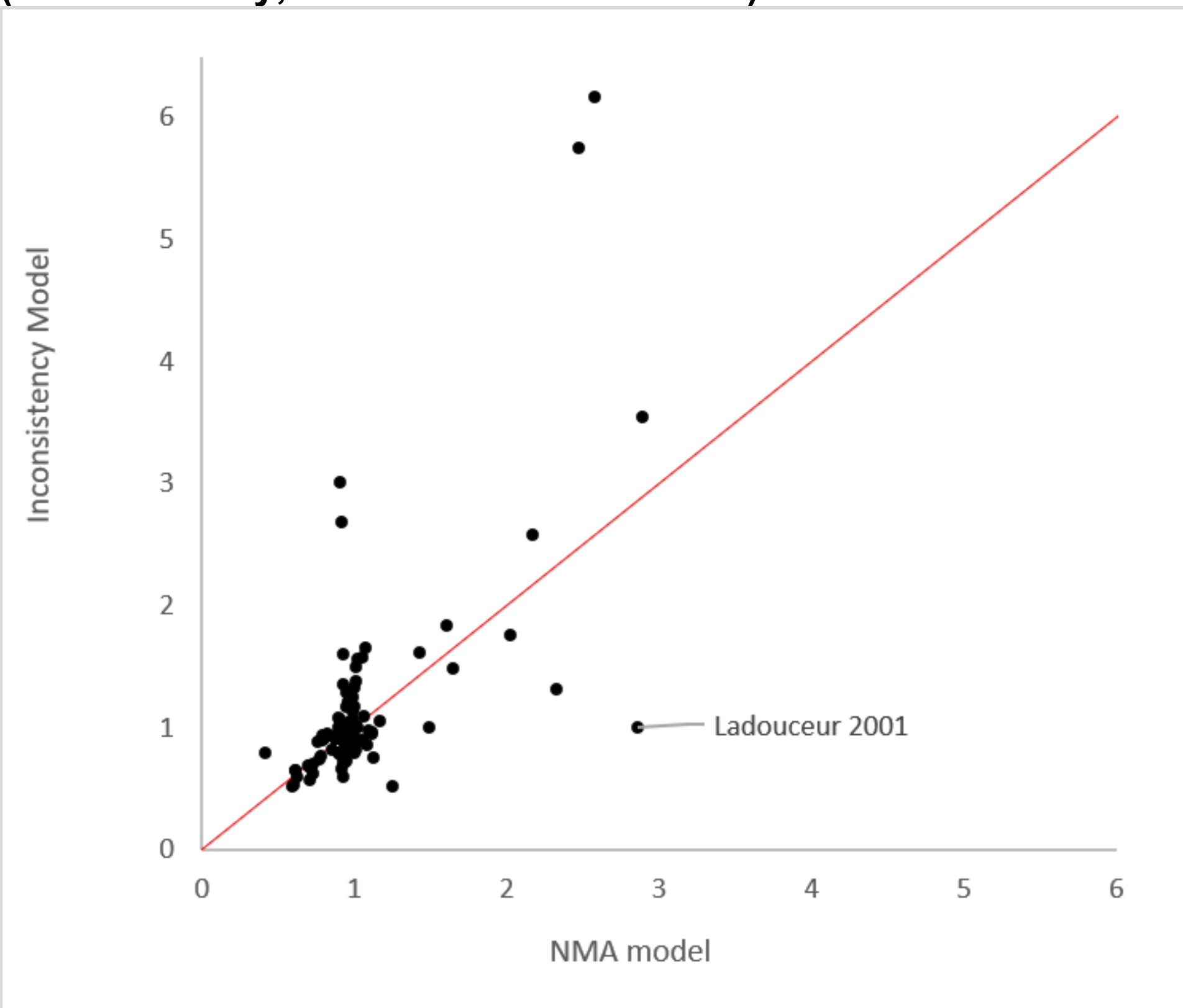

A data-point from Ladouceur 2001 is identified as potentially inconsistent.

Model fit statistics for the base case analysis for the outcome gambling frequency, using the full dataset

Model	Total residual deviance	Dbar	pD	DIC	Between-study SD (95% CrI)	Between-intervention SD (95% CrI)	Regression coefficient (95%CrI)
**RE study, FE class**	63.0	159.6	37.4	196.9	0.05 (0.00 to 0.14)	-	-
**FE study, FE class**	65.3	161.9	33.0	194.9	-	-	-
**RE study, RE class**	63.3	159.9	38.6	198.5	0.05 (0.00 to 0.13)	0.03 (0.00 to 0.11)	-
**Inconsistency Model [RE study, FE class]**	62.2	158.8	41.5	200.3	0.05 (0.00 to 0.14)	-	-
**Meta-Regression, sample size**	63.9	160.5	37.8	198.3	0.05 (0.00 to 0.14)	-	2.91 (-10.63 to 15.55)
**Meta-Regression, industry funding**	63.2	159.8	38.2	197.9	0.06 (0.01 to 0.14)	-	-0.04 (-0.24 to 0.15)

Models differed in their specification of effects structure on study effects and treatment class effects. 23 studies with 66 study arms.
CrI: credible intervals; FE: fixed effect; RE: random effects; SD: standard deviation

Dev-dev plot for gambling frequency, showing the posterior mean deviance for each data-point for the inconsistency model plotted against the NMA model (random study, fixed class effect model).

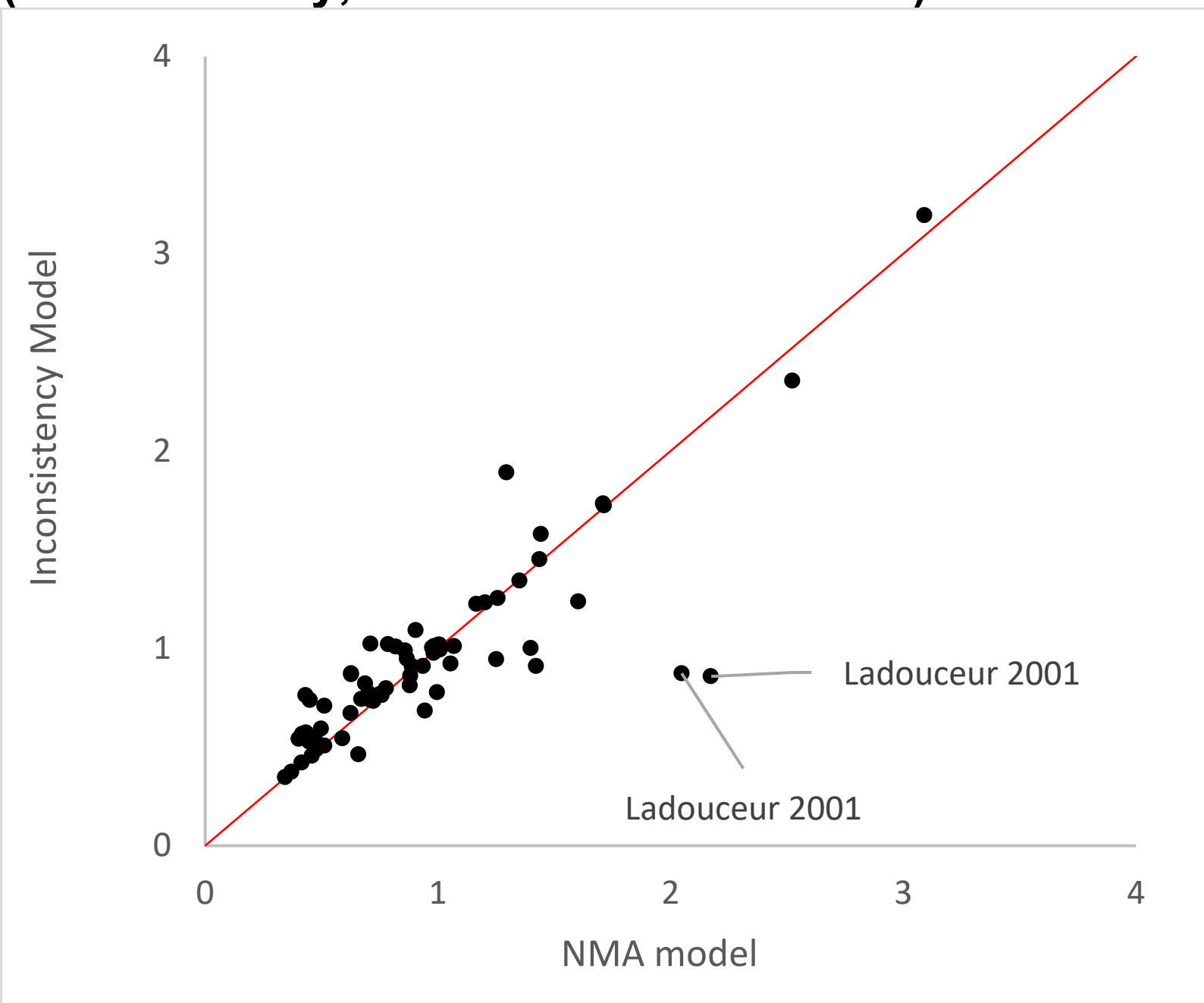

Data from study Ladouceur 2001 are identified as potentially inconsistent.

SUPPLEMENTARY MATERIAL S8: Gambling symptom severity NMA: treatment classes, interventions and numbers of participants

Interventions were classified into classes. Active intervention classes included in the gambling symptom severity NMA were: CBT individual; CBT group; behavioural therapies individual; counselling individual; motivational interviewing; self-help (with no or minimal support); guided self-help; couple interventions; twelve step group programme; selective serotonin reuptake inhibitors (SSRIs). Inactive intervention classes included in the gambling symptom severity NMA were: no treatment; treatment as usual (TAU); waitlist.

Treatment classes, interventions and numbers of participants tested on each

Class	N	Intervention	N
**No treatment**	771	No treatment	771
**TAU**	153	TAU (ranging from no treatment to a variety of other treatments including counselling)	8
		Information + referral	110
		Referral to Gamblers anonymous (GA) group	35
**Attention placebo**	271	Attention-control (non-gambling) feedback	114
		Sham computerised attentional bias modification	65
		Sham computerised cognitive training with support	92
**Waitlist**	558	Waitlist	558
**CBT individual**	592	Brief CBT individual	317
		Brief motivational interviewing + brief CBT individual	143
		CBT individual	104
		Brief Mindfulness-based cognitive therapy (MBCT) individual	28
**CBT group**	121	CBT group	121
**BT individual**	136	Behavioural therapy individual	73
		Exposure therapy individual	43
		Dialectical behavior therapy, modified for anger & addiction	20
**Counselling individual**	76	Client-centred therapy (CCT)	76
**Motivational interviewing**	303	Brief motivational interviewing	231
		Motivational interviewing	72
**Self-help (with no or minimal support)**	2555	Personalised feedback intervention	446
		Psychoeducational materials	182
		CBT workbook	366
		Computerised personalised feedback intervention	243
		Computerised CBT	689
		Chatbot-delivered CBT	163
		Computerised CBT for depression	71
		Computerised attentional bias modification	66
		Behaviour change SMS + accessing internet mental health service	99
		Computerised virtual peer support	230
**Guided self-help**	994	Brief motivational interviewing + CBT workbook	110
		CBT workbook with support	189
		CBT workbook with email support	14
		Psychoeducational materials with email support	56

		Computerised CBT with support	153
		Computerised behavioural couples therapy with support	68
		Computerised counselling with support	54
		Chatbot-delivered CBT with support	72
		Computerised motivational interviewing with support	30
		Computerised cognitive training with support	93
		Brief motivational interviewing + computerised CBT	155
**Couple interventions**	8	Congruence couple therapy	8
**Twelve step group programme**	11	Twelve-step facilitated group therapy	11
**SSRIs**	15	Escitalopram	15
**TOTAL**	6564		6564

BT: behavioural therapy; CBT: cognitive behavioural therapy; SSRIs: selective serotonin reuptake inhibitors; TAU: treatment as usual

SUPPLEMENTARY MATERIAL S9: Gambling symptom severity NMA: sensitivity analyses

Gambling symptom severity – base-case and sensitivity analyses: results, all treatments versus no treatment

Analysis	Full dataset		Intention-to-treat only		Completers only		No industry funding	
Number of RCTs	44		19		27		21	
**Class**	**N rand**	**SMD vs no treat (mean, 95% CrI)**	**N rand**	**SMD vs no treat (mean, 95% CrI)**	**N rand**	**SMD vs no treat (mean, 95% CrI)**	**N rand**	**SMD vs no treat (mean, 95% CrI)**
CBT group	121	**-1.09 (-1.75 to -0.44)**	27	**-2.22 (-3.48 to -0.91)**	144	**-1.00 (-1.79 to -0.24)**	80	-0.56 (-1.44 to 0.28)
CBT individual	592	**-0.56 (-1.07 to -0.07)**	461	**-0.59 (-1.07 to -0.10)**	212	-0.51 (-1.26 to 0.25)	276	-0.43 (-0.98 to 0.13)
Behavioural therapies individual	136	-0.59 (-1.41 to 0.23)	20	**-1.22 (-2.29 to -0.14)**	106	-0.31 (-1.43 to 0.80)		*Class not present*
Couple interventions	8	-0.48 (-2.22 to 1.28)		*Class not present*	8	-0.48 (-2.57 to 1.61)		*Class not present*
Counselling individual	76	-0.43 (-1.51 to 0.65)		*Class not present*	66	-0.27 (-1.59 to 1.04)		*Class not present*
SSRIs	15	-0.46 (-1.92 to 0.99)		*Class not present*	15	-0.42 (-2.11 to 1.26)	15	-0.33 (-1.74 to 1.07)
Twelve step group programme	11	-0.38 (-1.91 to 1.11)		*Class not present*	11	-0.27 (-1.97 to 1.38)		*Class not present*
Motivational interviewing	303	-0.30 (-0.84 to 0.24)		*Class not present*	309	-0.16 (-0.84 to 0.51)	271	-0.14 (-0.68 to 0.40)
Guided self-help	994	-0.06 (-0.56 to 0.44)	309	**-0.58 (-1.18 to -0.04)**	533	0.23 (-0.51 to 0.99)	763	-0.16 (-0.73 to 0.42)
No treatment	771	*Reference*	363	*Reference*	378	*Reference*	588	*Reference*
Attention placebo	271	0.04 (-0.75 to 0.82)	157	-0.54 (-1.20 to 0.09)	134	0.54 (-0.66 to 1.73)	92	-0.10 (-1.31 to 1.12)
Self-help (with no or minimal support)	2555	0.01 (-0.36 to 0.37)	1997	-0.28 (-0.59 to 0.03)	872	0.24 (-0.36 to 0.85)	1979	-0.09 (-0.49 to 0.32)
TAU	153	0.16 (-0.69 to 0.99)	70	0.74 (-0.14 to 1.60)	100	0.15 (-1.11 to 1.42)	180	0.20 (-0.61 to 1.02)
Waitlist	558	**0.96 (0.47 to 1.46)**	310	**0.54 (0.01 to 1.22)**	369	**1.20 (0.51 to 1.92)**	309	**1.04 (0.40 to 1.72)**
TOTAL	6564		3714		3257		4553	

CBT: cognitive behavioural therapy; CrI: credible intervals; N rand: number randomised; SMD: standardised mean difference; SSRIs: selective serotonin reuptake inhibitors; TAU: treatment as usual

Negative effect values indicate a favourable outcome compared with no treatment. Results where 95% CrI do not cross the no effect line are shown in bold.

Gambling symptom severity – base-case and sensitivity analyses: Network plots

Base-case (full dataset)

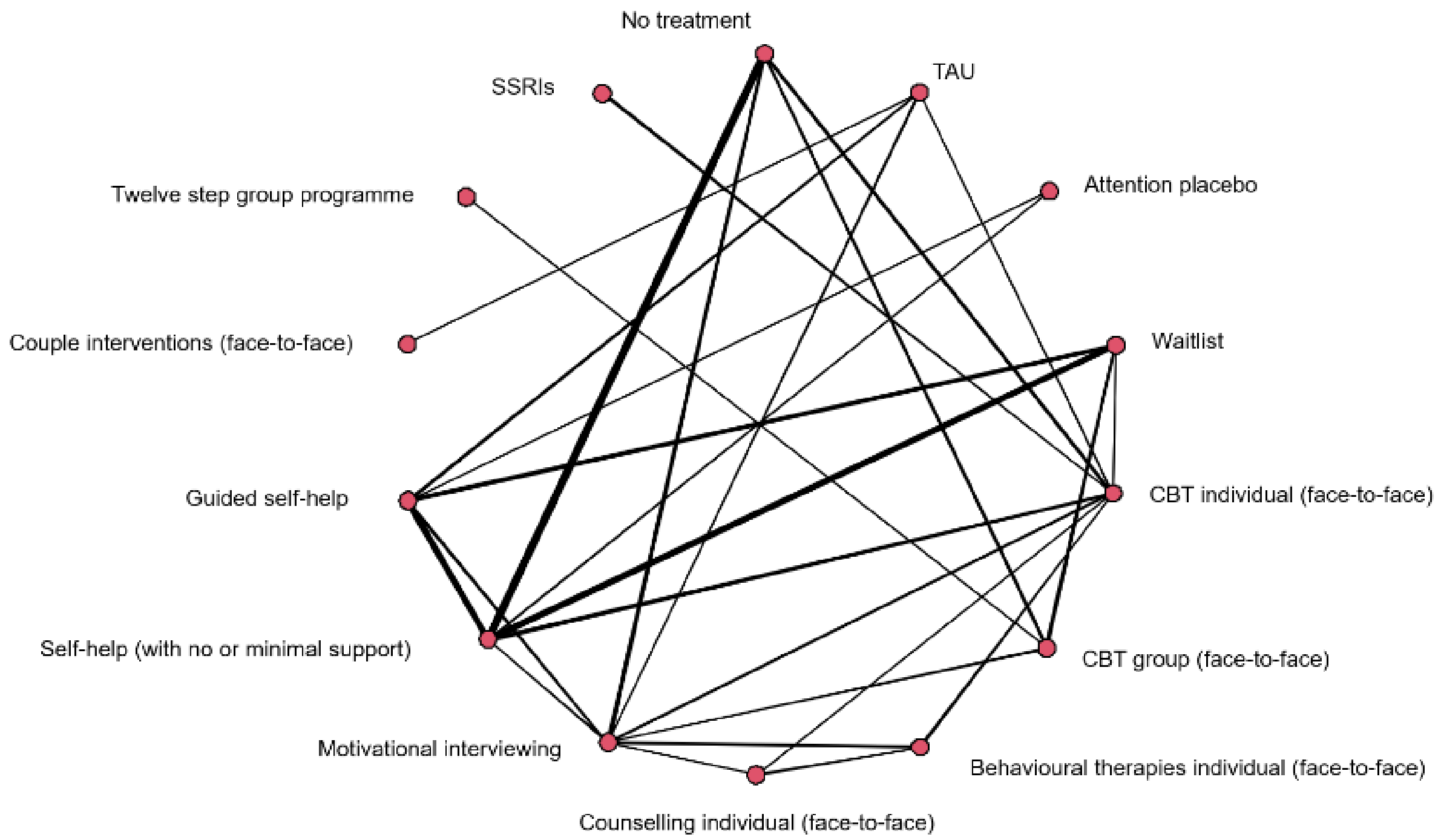

Intention-to-treat only

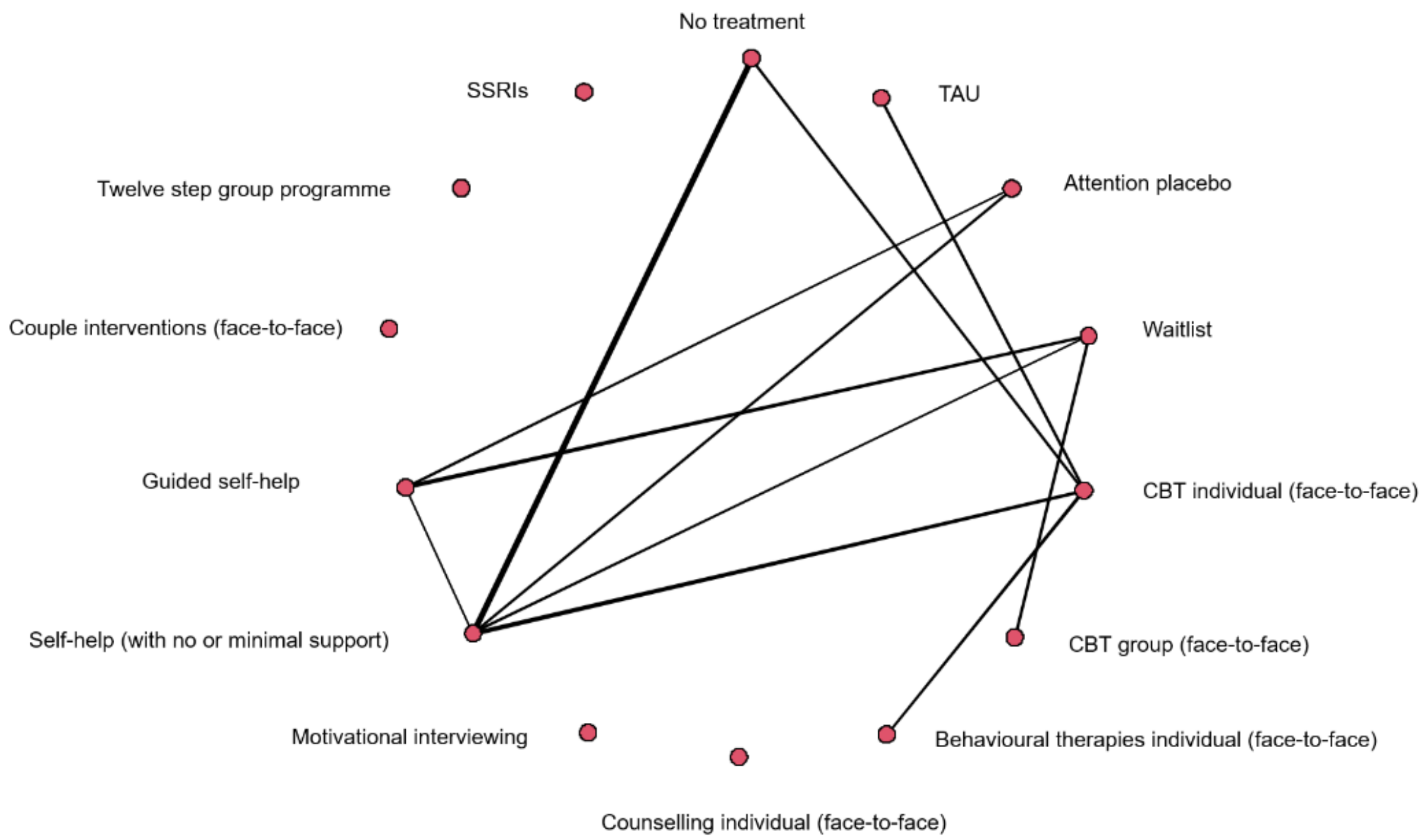

Completers only

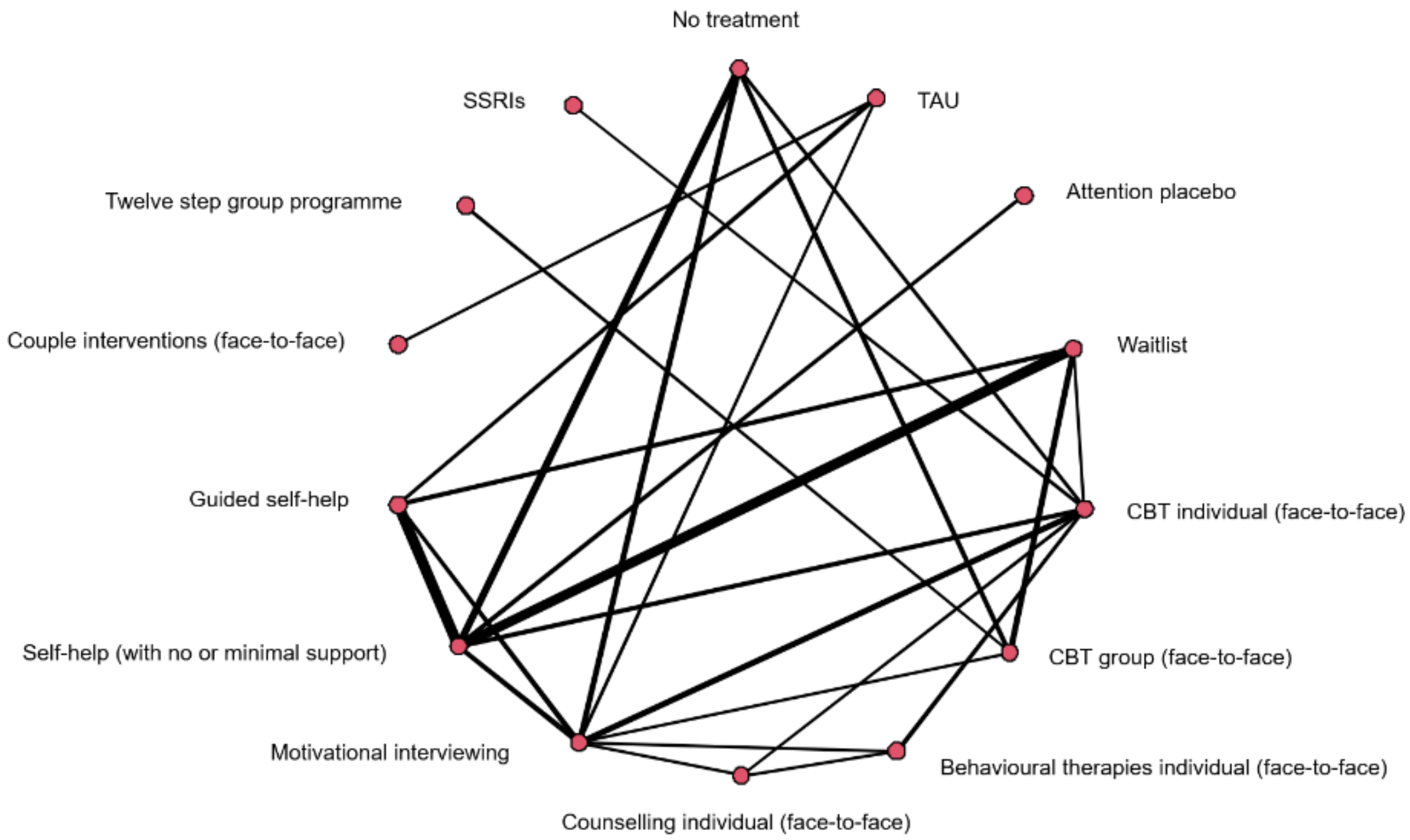

No industry funding

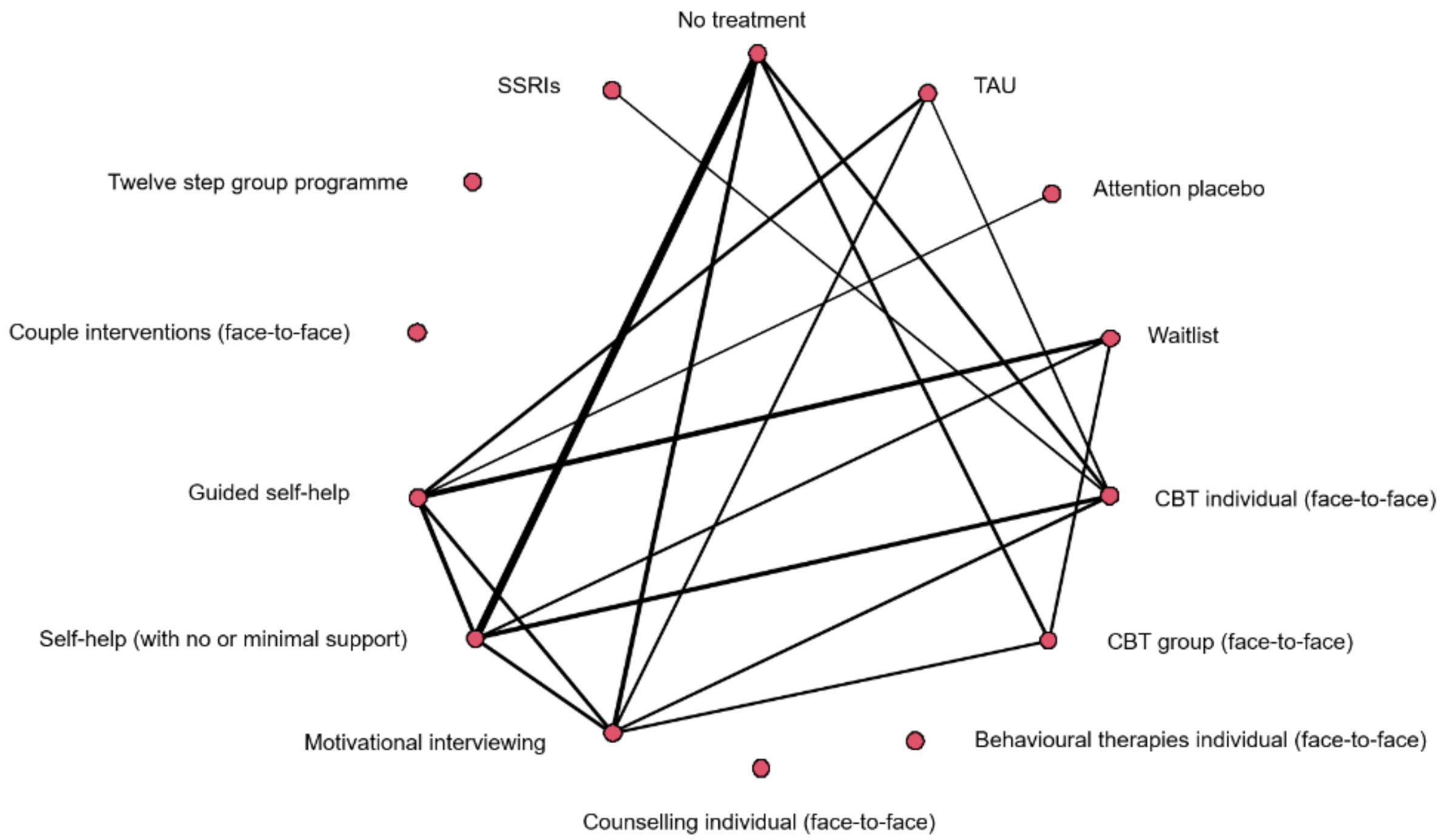

Gambling symptom severity – base-case and sensitivity analyses: Model fit statistics

Model	Total number of datapoints	Total residual deviance	Between-study SD (95% CrI)
**Base-case (full dataset)**	128	132.5	0.57 (0.44 to 0.73)
**Intention-to-treat only**	57	72.4	0.27 (0.07 to 0.55)
**Completers only**	75	73.0	0.65 (0.46 to 0.91)
**No industry funding**	68	71.1	0.51 (0.35 to 0.74)

CrI: Credible Intervals; SD: standard deviation

SUPPLEMENTARY MATERIAL S10: Gambling symptom severity NMA: relative effects between all pairs of treatment classes

Comparison	Mean SMD (95% CrI)
TAU vs. no treatment	0.16 (-0.69 to 0.99)
Attention placebo vs. no treatment	0.04 (-0.75 to 0.82)
Waitlist vs. no treatment	**0.96 (0.47 to 1.46)**
CBT individual (face-to-face) vs. no treatment	**-0.56 (-1.07 to -0.07)**
CBT group (face-to-face) vs. no treatment	**-1.09 (-1.75 to -0.44)**
BT individual (face-to-face) vs. no treatment	-0.59 (-1.41 to 0.23)
Counselling individual (face-to-face) vs. no treatment	-0.43 (-1.51 to 0.65)
Motivational interviewing vs. no treatment	-0.30 (-0.84 to 0.24)
Self-help (with no or minimal support) vs. no treatment	0.01 (-0.36 to 0.37)
Guided self-help vs. no treatment	-0.06 (-0.56 to 0.44)
Couple interventions (face-to-face) vs. no treatment	-0.48 (-2.22 to 1.28)
Twelve step group programme vs. no treatment	-0.38 (-1.91 to 1.11)
SSRIs vs. no treatment	-0.46 (-1.92 to 0.99)
Attention placebo vs. TAU	-0.12 (-1.16 to 0.93)
Waitlist vs. TAU	0.80 (-0.04 to 1.65)
CBT individual (face-to-face) vs. TAU	-0.72 (-1.52 to 0.08)
CBT group (face-to-face) vs. TAU	**-1.25 (-2.24 to -0.27)**
BT individual (face-to-face) vs. TAU	-0.74 (-1.77 to 0.29)
Counselling individual (face-to-face) vs. TAU	-0.59 (-1.84 to 0.66)
Motivational interviewing vs. TAU	-0.46 (-1.30 to 0.38)
Self-help (with no or minimal support) vs. TAU	-0.15 (-0.95 to 0.66)
Guided self-help vs. TAU	-0.22 (-1.02 to 0.58)
Couple interventions (face-to-face) vs. TAU	-0.64 (-2.16 to 0.89)
Twelve step group programme vs. TAU	**-0.54 (-2.23 to 1.11)**
SSRIs vs. TAU	-0.62 (-2.21 to 0.96)
Waitlist vs. attention placebo	**0.92 (0.15 to 1.70)**
CBT individual (face-to-face) vs. attention placebo	-0.60 (-1.44 to 0.23)
CBT group (face-to-face) vs. attention placebo	**-1.13 (-2.08 to -0.20)**
BT individual (face-to-face) vs. attention placebo	-0.62 (-1.68 to 0.42)
Counselling individual (face-to-face) vs. attention placebo	-0.47 (-1.75 to 0.80)
Motivational interviewing vs. attention placebo	-0.34 (-1.19 to 0.51)
Self-help (with no or minimal support) vs. attention placebo	-0.03 (-0.73 to 0.67)
Guided self-help vs. attention placebo	-0.10 (-0.83 to 0.63)
Couple interventions (face-to-face) vs. attention placebo	-0.52 (-2.37 to 1.34)
Twelve step group programme vs. attention placebo	-0.42 (-2.08 to 1.21)
SSRIs vs. attention placebo	-0.50 (-2.12 to 1.11)
CBT individual (face-to-face) vs. waitlist	**-1.52 (-2.08 to -0.98)**

CBT group (face-to-face) vs. waitlist	**-2.05 (-2.71 to -1.41)**
BT individual (face-to-face) vs. waitlist	**-1.54 (-2.40 to -0.71)**
Counselling individual (face-to-face) vs. waitlist	**-1.39 (-2.50 to -0.30)**
Motivational interviewing vs. waitlist	**-1.26 (-1.86 to -0.67)**
Self-help (with no or minimal support) vs. waitlist	**-0.95 (-1.34 to -0.57)**
Guided self-help vs. waitlist	**-1.02 (-1.47 to -0.58)**
Couple interventions (face-to-face) vs. waitlist	-1.44 (-3.18 to 0.31)
Twelve step group programme vs. waitlist	-1.34 (-2.83 to 0.13)
SSRIs vs. waitlist	-1.42 (-2.91 to 0.05)
CBT group (face-to-face) vs. CBT individual (face-to-face)	-0.53 (-1.27 to 0.20)
BT individual (face-to-face) vs. CBT individual (face-to-face)	-0.02 (-0.71 to 0.66)
Counselling individual (face-to-face) vs. CBT individual (face-to-face)	0.13 (-0.88 to 1.15)
Motivational interviewing vs. CBT individual (face-to-face)	0.26 (-0.28 to 0.80)
Self-help (with no or minimal support) vs. CBT individual (face-to-face)	**0.57 (0.11 to 1.04)**
Guided self-help vs. CBT individual (face-to-face)	0.50 (-0.05 to 1.05)
Couple interventions (face-to-face) vs. CBT individual (face-to-face)	0.08 (-1.64 to 1.82)
Twelve step group programme vs. CBT individual (face-to-face)	0.18 (-1.37 to 1.70)
SSRIs vs. CBT individual (face-to-face)	0.10 (-1.27 to 1.47)
BT individual (face-to-face) vs. CBT group (face-to-face)	0.50 (-0.46 to 1.48)
Counselling individual (face-to-face) vs. CBT group (face-to-face)	0.66 (-0.53 to 1.86)
Motivational interviewing vs. CBT group (face-to-face)	**0.79 (0.06 to 1.53)**
Self-help (with no or minimal support) vs. CBT group (face-to-face)	**1.10 (0.45 to 1.76)**
Guided self-help vs. CBT group (face-to-face)	**1.03 (0.33 to 1.74)**
Couple interventions (face-to-face) vs. CBT group (face-to-face)	0.61 (-1.20 to 2.44)
Twelve step group programme vs. CBT group (face-to-face)	0.71 (-0.72 to 2.11)
SSRIs vs. CBT group (face-to-face)	0.63 (-0.92 to 2.18)
Counselling individual (face-to-face) vs. BT individual (face-to-face)	0.15 (-0.92 to 1.22)
Motivational interviewing vs. BT individual (face-to-face)	0.28 (-0.51 to 1.08)
Self-help (with no or minimal support) vs. BT individual (face-to-face)	0.59 (-0.19 to 1.39)
Guided self-help vs. BT individual (face-to-face)	0.52 (-0.32 to 1.37)
Couple interventions (face-to-face) vs. BT individual (face-to-face)	0.10 (-1.72 to 1.96)
Twelve step group programme vs. BT individual (face-to-face)	0.20 (-1.48 to 1.85)
SSRIs vs. BT individual (face-to-face)	0.12 (-1.40 to 1.66)
Motivational interviewing vs. counselling individual (face-to-face)	0.13 (-0.90 to 1.16)
Self-help (with no or minimal support) vs. counselling individual (face-to-face)	0.44 (-0.62 to 1.51)
Guided self-help vs. counselling individual (face-to-face)	0.37 (-0.73 to 1.47)
Couple interventions (face-to-face) vs. counselling individual (face-to-face)	-0.05 (-2.01 to 1.95)
Twelve step group programme vs. counselling individual (face-to-face)	0.05 (-1.77 to 1.84)
SSRIs vs. counselling individual (face-to-face)	-0.03 (-1.73 to 1.67)
Self-help (with no or minimal support) vs. motivational interviewing	0.31 (-0.21 to 0.83)
Guided self-help vs. motivational interviewing	0.24 (-0.33 to 0.82)

Couple interventions (face-to-face) vs. motivational interviewing	-0.18 (-1.92 to 1.58)
Twelve step group programme vs. motivational interviewing	-0.08 (-1.64 to 1.44)
SSRIs vs. motivational interviewing	-0.16 (-1.63 to 1.31)
Guided self-help vs. self-help (with no or minimal support)	-0.07 (-0.45 to 0.31)
Couple interventions (face-to-face) vs. self-help (with no or minimal support)	-0.49 (-2.20 to 1.25)
Twelve step group programme vs. self-help (with no or minimal support)	-0.39 (-1.90 to 1.09)
SSRIs vs. self-help (with no or minimal support)	-0.47 (-1.92 to 0.98)
Couple interventions (face-to-face) vs. guided self-help	-0.42 (-2.13 to 1.31)
Twelve step group programme vs. guided self-help	-0.32 (-1.85 to 1.18)
SSRIs vs. guided self-help	-0.40 (-1.88 to 1.08)
Twelve step group programme vs. couple interventions (face-to-face)	0.10 (-2.18 to 2.33)
SSRIs vs. couple interventions (face-to-face)	0.02 (-2.19 to 2.21)
SSRIs vs. Twelve step group programme	-0.08 (-2.13 to 1.98)

BT: behavioural therapies; CBT: cognitive behavioural therapy; CrI: credible intervals; SMD: standardised mean difference; SSRIs: selective serotonin reuptake inhibitors; TAU: treatment as usual

Negative values indicate a favourable outcome of the first treatment class in the comparison. Results where the 95% CrI do not cross the line of no effect are shown in bold.

SUPPLEMENTARY MATERIAL S11: Gambling frequency NMA: treatment classes, interventions and numbers of participants

Interventions were classified into classes. Active intervention classes included in the gambling frequency NMA were: CBT individual; CBT group; behavioural therapies individual; counselling individual; motivational interviewing; self-help (with no or minimal support); guided self-help. Inactive intervention classes included in the gambling frequency NMA were: no treatment; treatment as usual (TAU); attention placebo; waitlist.

Treatment classes, interventions and numbers of participants tested on each

Class	N	Intervention	N
**No treatment**	682	No treatment	682
**TAU**	111	Information + referral	111
**Attention placebo**	39	Brief semi-structured interview	39
**Waitlist**	401	Waitlist	401
**CBT individual**	331	Brief CBT individual	183
		Brief motivational interviewing + brief CBT individual	103
		CBT individual	45
**CBT group**	30	CBT group	30
**BT individual**	98	Behavioural therapy individual	98
**Counselling individual**	76	Client-centred therapy (CCT)	76
**Motivational interviewing**	290	Brief motivational interviewing	195
		Motivational interviewing	95
**Self-help (with no or minimal support)**	1756	Personalised feedback intervention	349
		Psychoeducational materials	182
		Psychoeducational workbook	213
		CBT workbook	199
		Computerised personalised feedback intervention	243
		Computerised CBT	145
		Chatbot-delivered CBT	96
		Behaviour change SMS + accessing internet mental health service	99
		Computerised virtual peer support	230
**Guided self-help**	608	Brief motivational interviewing + CBT workbook	223
		CBT workbook with support	224
		Psychoeducational materials with email support	56
		Computerised CBT with support	51
		Computerised counselling with support	54
**TOTAL**	4422		4422

BT: behavioural therapy; CBT: cognitive behavioural therapy; SSRIs: selective serotonin reuptake inhibitors; TAU: treatment as usual

SUPPLEMENTARY MATERIAL S12: Gambling frequency NMA: network of treatment classes

Figure S3. Gambling frequency network plot – full dataset containing 66 treatment arms, 11 treatment classes and 26 interventions, 4,422 participants

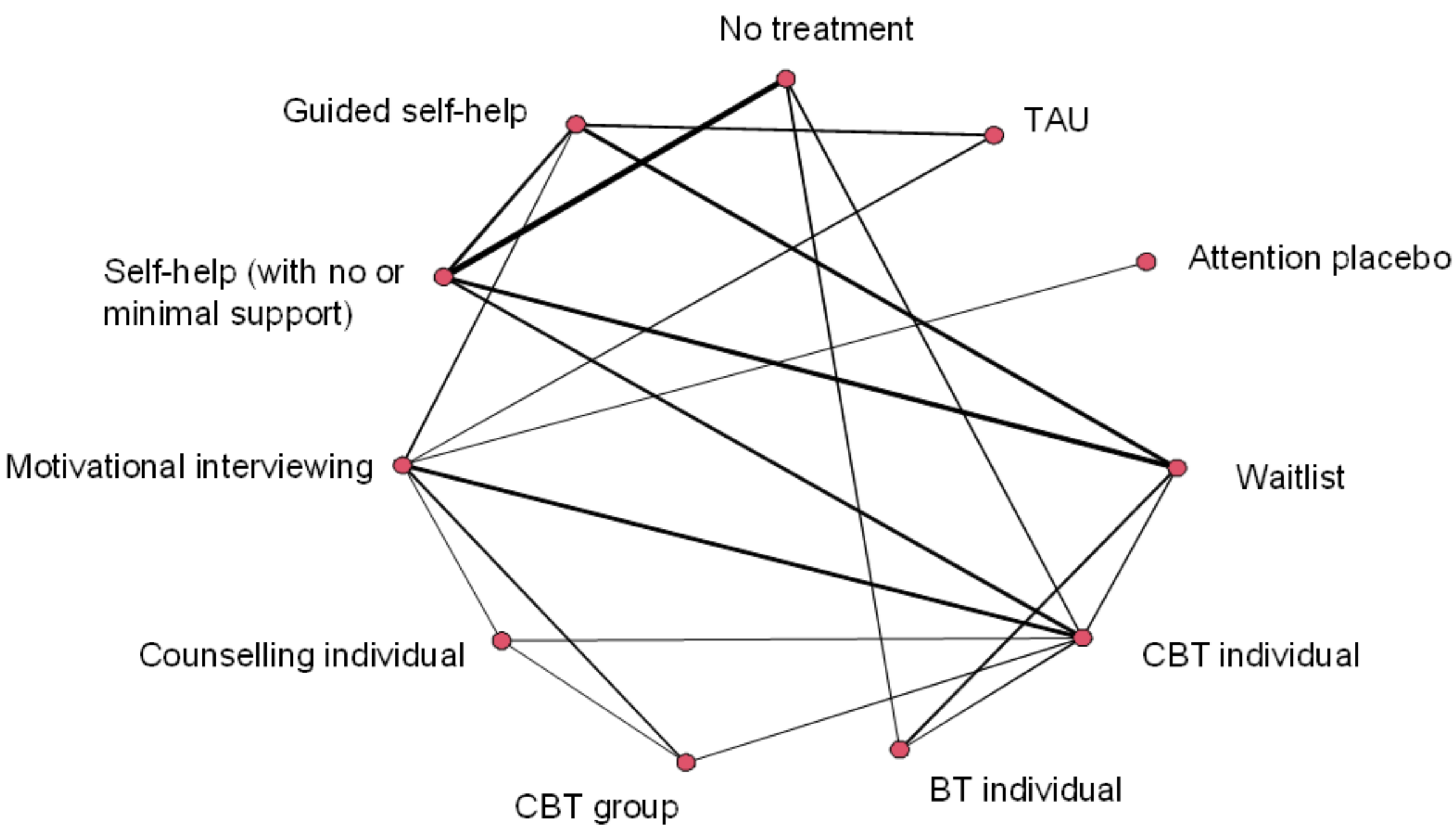

BT: behavioural therapy; CBT: cognitive behavioural therapy; TAU: treatment as usual

SUPPLEMENTARY MATERIAL S13: Gambling frequency NMA: results versus no treatment

Gambling frequency results – full dataset: posterior standardised mean difference (SMD) of all treatments versus no treatment

Treatment class	N rand	SMD vs no treatment (mean, 95% CrI)	Rank (mean, 95% CrI)
TAU	111	**-0.42 (-0.72 to -0.14)**	4.67 (1 to 10)
Behavioural therapies individual	98	**-0.41 (-0.68 to -0.14)**	4.79 (2 to 9)
CBT individual	331	**-0.36 (-0.55 to -0.19)**	5.52 (2 to 9)
Attention placebo	39	-0.37 (-0.82 to 0.08)	6.05 (1 to 12)
CBT group	30	-0.34 (-0.79 to 0.08)	6.32 (1 to 12)
Guided self-help	608	**-0.31 (-0.48 to -0.14)**	6.77 (3 to 10)
Motivational interviewing	290	**-0.30 (-0.51 to -0.10)**	7.07 (4 to 10)
Counselling individual	76	-0.25 (-0.58 to 0.08)	7.94 (3 to 12)
Self-help (with no or minimal support)	1756	**-0.15 (-0.25 to -0.06)**	9.72 (7 to 12)
No treatment	682	Reference	11.76 (10 to 14)
Waitlist	401	0.01 (-0.15 to 0.18)	11.87 (10 to 14)

CBT: cognitive behavioural therapy; CrI: credible intervals; N rand: number randomised; SMD: standardised mean difference; TAU: treatment as usual

Treatment classes ordered from best to worst, according to mean rankings. Negative effect values indicate a favourable outcome compared with no treatment. Results where 95% CrI do not cross the no effect line are shown in bold.

SUPPLEMENTARY MATERIAL S14: Gambling frequency NMA: forest plots versus no treatment

Gambling frequency forest plot – full dataset. Standardised mean difference versus no treatment (N=682). Vertical axis shows effect of no treatment. Values on the left side of the vertical axis indicate better effect compared with no treatment.

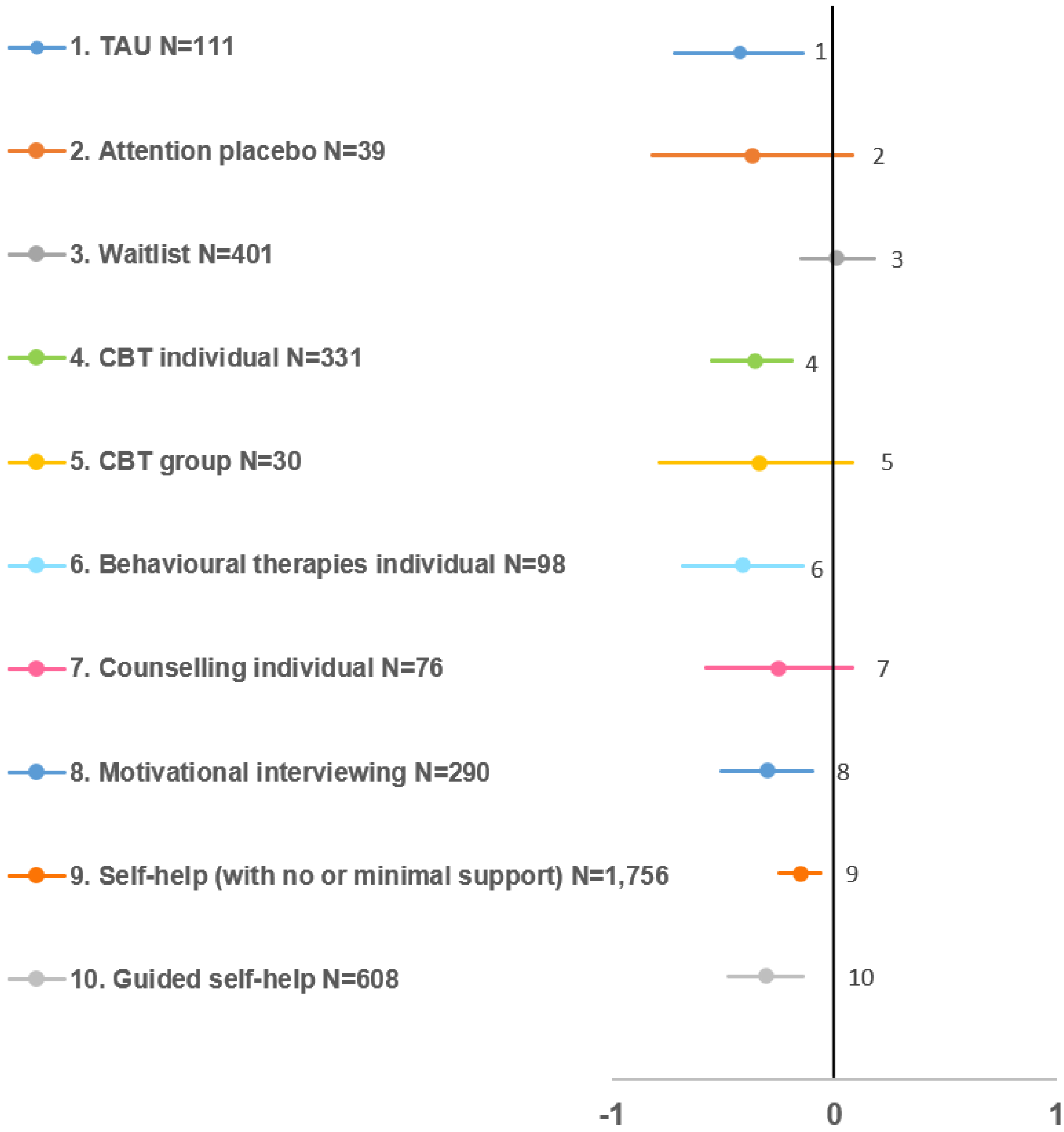

CBT: cognitive behavioural therapy; TAU: treatment as usual

SUPPLEMENTARY MATERIAL S15: Gambling frequency NMA: sensitivity analyses

Gambling frequency – base-case and sensitivity analyses: network plots and results, all treatments versus no treatment

**Analysis**	**Full dataset**		**Intention-to-treat only**		**Completers only**		**No industry funding**	
Number of RCTs	23		10		13		11	
**Class**	**N rand**	**SMD vs no treat (mean, 95% CrI)**	**N rand**	**SMD vs no treat (mean, 95% CrI)**	**N rand**	**SMD vs no treat (mean, 95% CrI)**	**N rand**	**SMD vs no treat (mean, 95% CrI)**
TAU	111	**-0.42 (-0.72 to -0.14)**	-	*Class not present*	100	**-0.41 (-0.78 to -0.03)**	111	**-0.48 (-0.80 to -0.15)**
Behavioural therapies individual	98	**-0.41 (-0.68 to -0.14)**	24	-0.20 (-0.86 to 0.46)	66	**-0.47 (-0.87 to -0.06)**	-	*Class not present*
CBT individual	331	**-0.36 (-0.55 to -0.19)**	191	**-0.31 (-0.55 to -0.07)**	125	**-0.48 (-0.81 to -0.15)**	105	-0.21 (-0.47 to 0.04)
Attention placebo	39	-0.37 (-0.82 to 0.08)	-	*Class not present*	39	-0.38 (-0.89 to 0.16)	-	*Class not present*
CBT group	30	-0.34 (-0.79 to 0.08)	-	*Class not present*	30	-0.36 (-0.82 to 0.10)	-	*Class not present*
Guided self-help	608	**-0.31 (-0.48 to -0.14)**	110	**-0.57 (-1.00 to -0.13)**	424	**-0.31 (-0.58 to -0.04)**	557	**-0.37 (-0.60 to -0.13)**
Motivational interviewing	290	**-0.30 (-0.51 to -0.10)**	50	-0.02 (-0.55 to 0.53)	225	**-0.31 (-0.61 to -0.01)**	125	**-0.36 (-0.64 to -0.09)**
Counselling individual	76	-0.25 (-0.58 to 0.08)	-	*Class not present*	67	-0.29 (-0.72 to 0.15)	-	*Class not present*
Self-help (with no or minimal support)	1756	**-0.15 (-0.25 to -0.06)**	1309	**-0.20 (-0.33 to -0.07)**	336	-0.14 (-0.35 to 0.06)	895	**-0.15 (-0.27 to -0.03)**
No treatment	682	*Reference*	363	*Reference*	249	Reference	458	*Reference*
Waitlist	401	0.01 (-0.15 to 0.18)	228	-0.11 (-0.36 to 0.14)	169	0.05 (-0.25 to 0.36)	186	-0.04 (-0.30, 0.23)
TOTAL	4422		2275		1830		2437	

CBT: cognitive behavioural therapy; CrI: credible intervals; N rand: number randomised; SMD: standardised mean difference; TAU: treatment as usual
Negative effect values indicate a favourable outcome compared with no treatment. Results where 95% CrI do not cross the no effect line are shown in bold.

Gambling frequency – base-case and sensitivity analyses: Network plots

Base-case (full dataset)

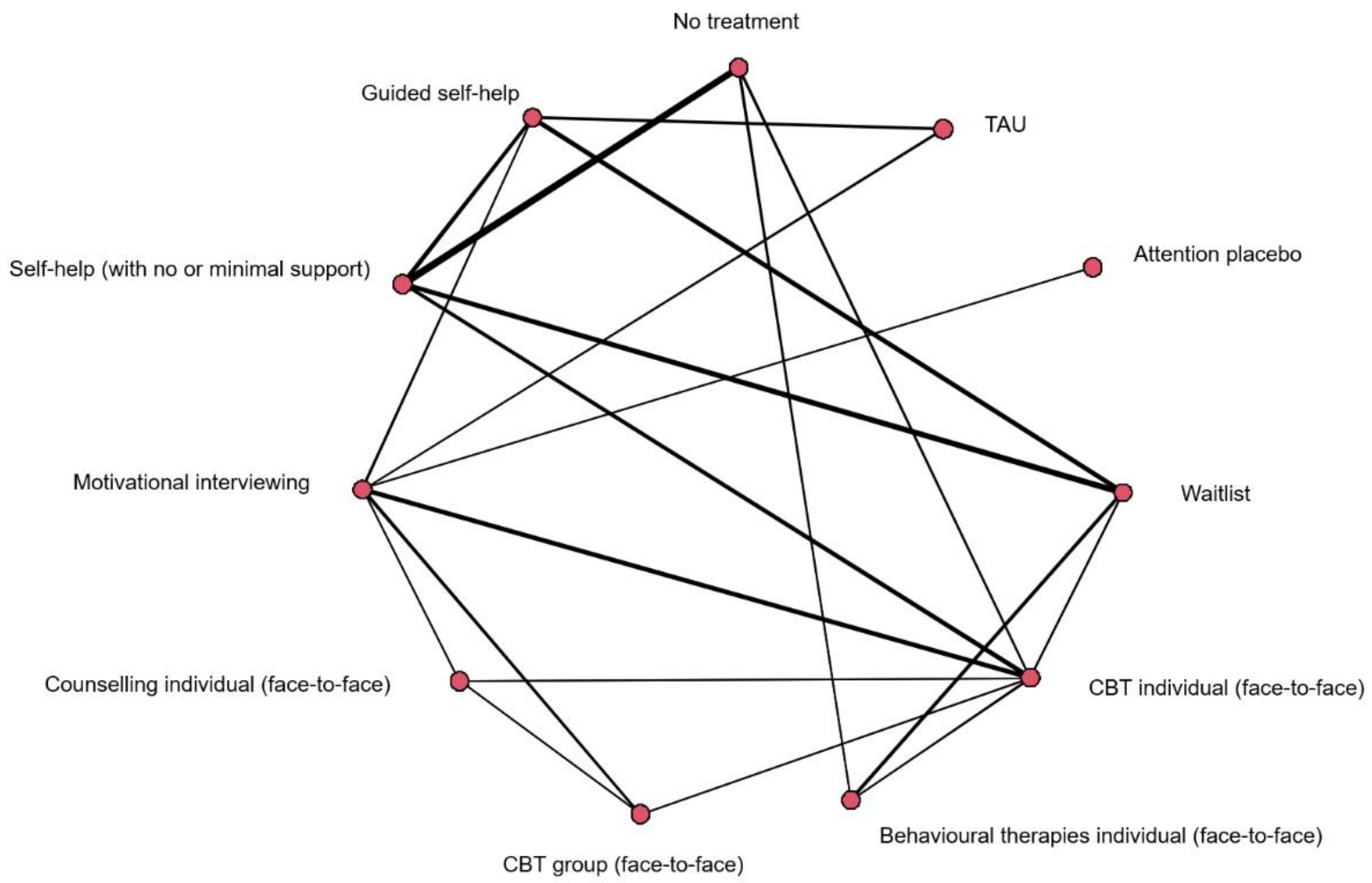

Intention-to-treat only

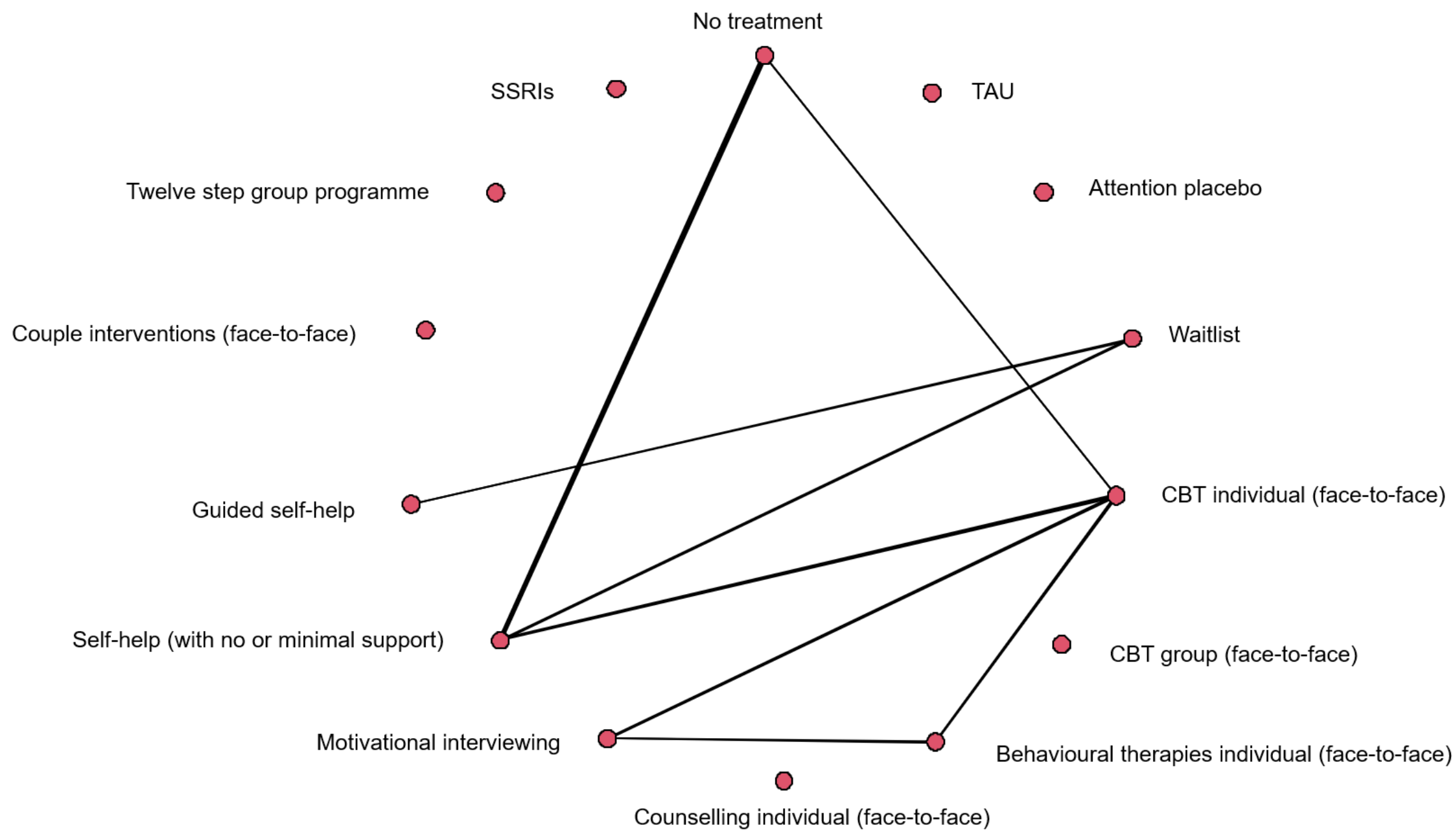

Completers only

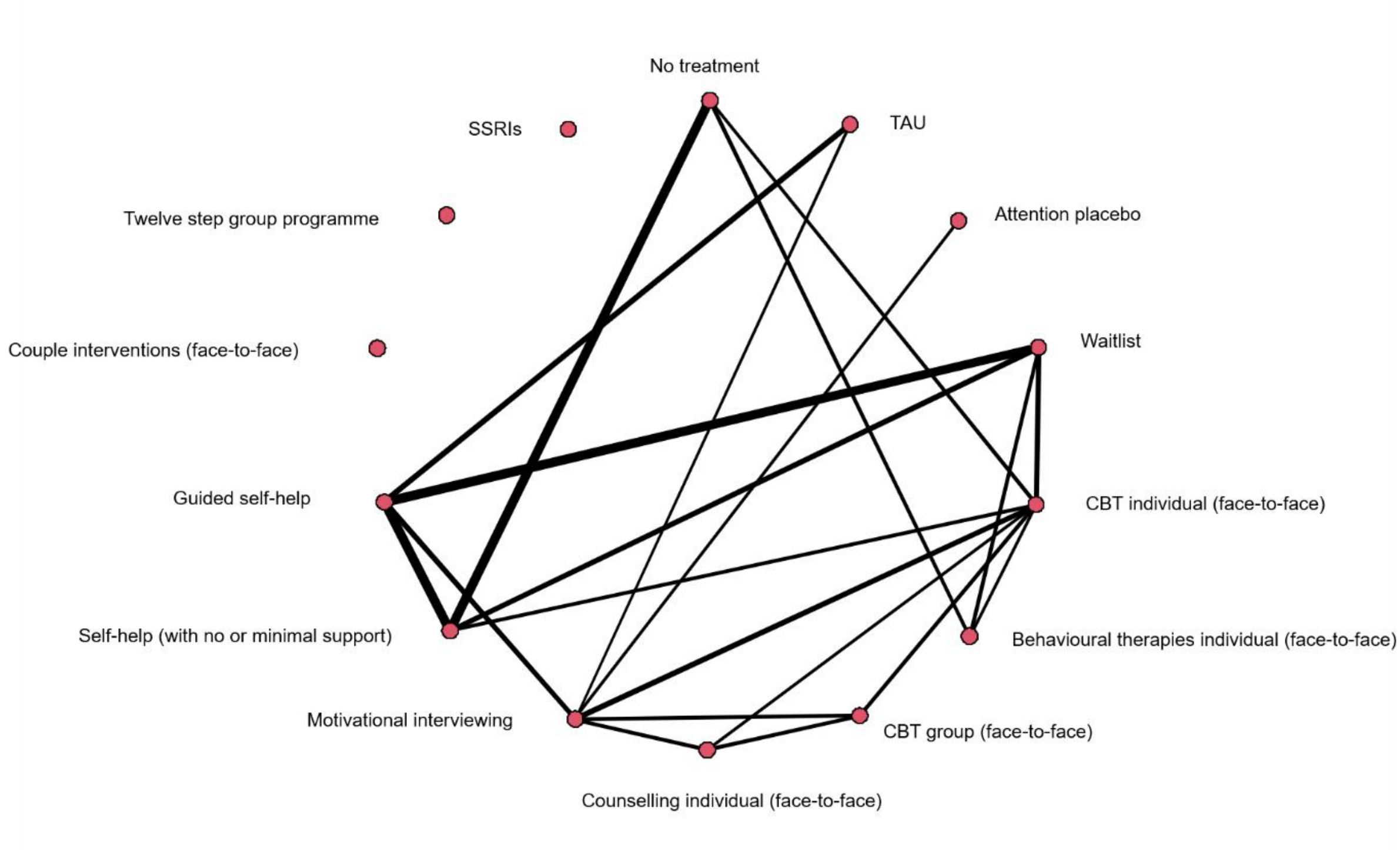

No industry funding

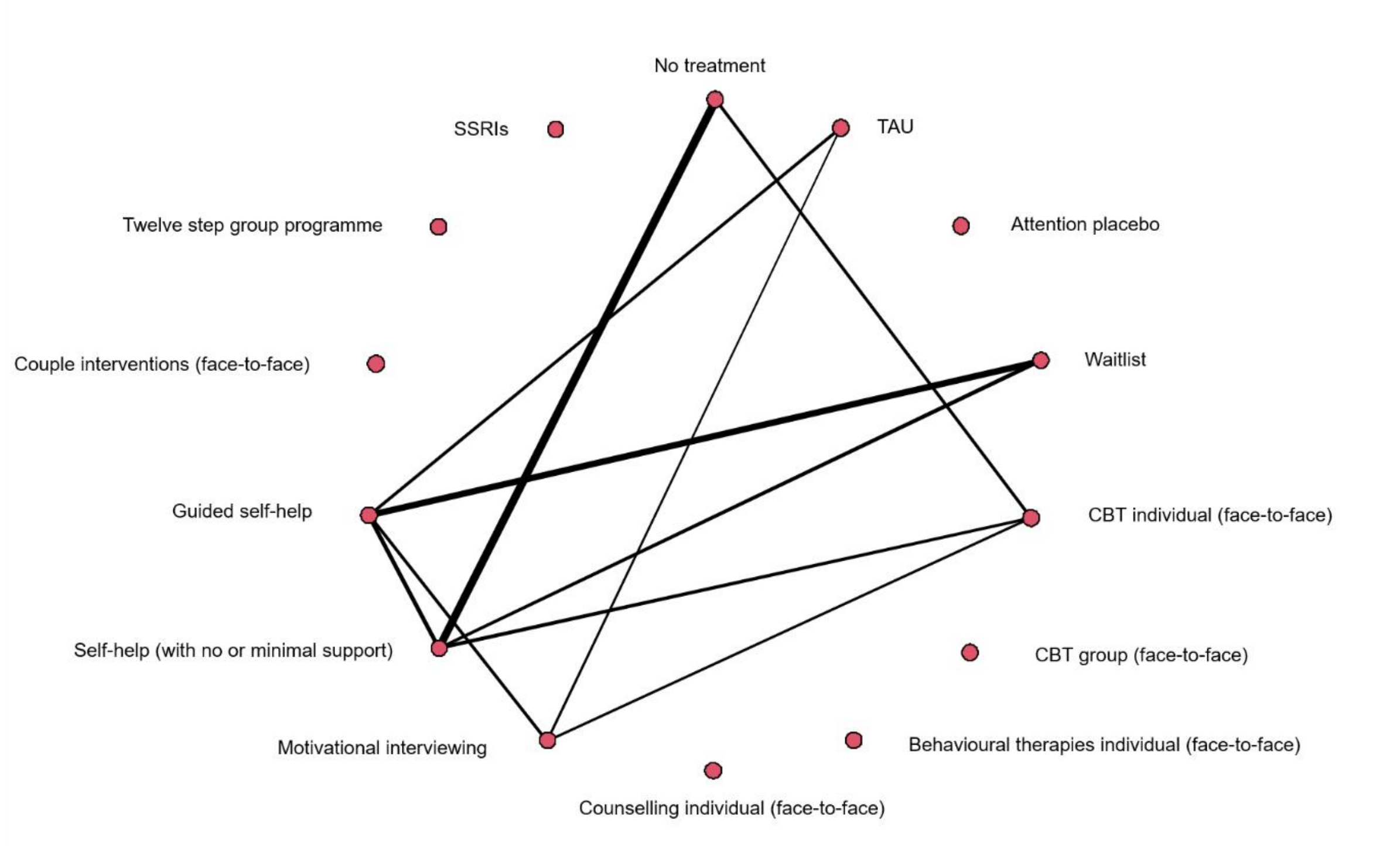

Gambling frequency – base-case and sensitivity analyses: Model fit statistics

Model	Total number of datapoints	Total residual deviance	Between-study SD (95% CrI)
**Base-case (full dataset)**	66	63.0	0.05 (0.00 to 0.14)
**Intention-to-treat only**	30	29.9	0.07 (0.00 to 0.20)
**Completers only**	36	31.4	0.07 (0.00 to 0.21)
**No industry funding**	33	31.4	0.07 (0.00 to 0.18)

CrI: Credible Intervals; SD: standard deviation

SUPPLEMENTARY MATERIAL S16: Gambling frequency NMA: relative effects between all pairs of treatment classes

Comparison	Mean SMD (95% CrI)
TAU vs. no treatment	**-0.42 (-0.72 to -0.14)**
Attention placebo vs. no treatment	-0.37 (-0.82 to 0.08)
Waitlist vs. no treatment	0.01 (-0.15 to 0.18)
CBT individual (face-to-face) vs. no treatment	**-0.36 (-0.55 to -0.19)**
CBT group (face-to-face) vs. no treatment	-0.34 (-0.79 to 0.08)
BT individual (face-to-face) vs. no treatment	**-0.41 (-0.68 to -0.14)**
Counselling individual (face-to-face) vs. no treatment	-0.25 (-0.58 to 0.08)
Motivational interviewing vs. no treatment	**-0.30 (-0.51 to -0.10)**
Self-help (with no or minimal support) vs. no treatment	**-0.15 (-0.25 to -0.06)**
Guided self-help vs. no treatment	**-0.31 (-0.48 to -0.14)**
Attention placebo vs. TAU	0.06 (-0.41 to 0.53)
Waitlist vs. TAU	**0.44 (0.16 to 0.72)**
CBT individual (face-to-face) vs. TAU	0.06 (-0.23 to 0.36)
CBT group (face-to-face) vs. TAU	0.08 (-0.43 to 0.59)
BT individual (face-to-face) vs. TAU	0.02 (-0.32 to 0.35)
Counselling individual (face-to-face) vs. TAU	0.18 (-0.23 to 0.56)
Motivational interviewing vs. TAU	0.12 (-0.14 to 0.40)
Self-help (with no or minimal support) vs. TAU	**0.27 (0.00 to 0.56)**
Guided self-help vs. TAU	0.11 (-0.13 to 0.36)
Waitlist vs. attention placebo	0.38 (-0.08 to 0.84)
CBT individual (face-to-face) vs. attention placebo	0.00 (-0.45 to 0.46)
CBT group (face-to-face) vs. attention placebo	0.02 (-0.59 to 0.65)
BT individual (face-to-face) vs. attention placebo	-0.04 (-0.51 to 0.43)
Counselling individual (face-to-face) vs. attention placebo	0.12 (-0.38 to 0.62)
Motivational interviewing vs. attention placebo	0.07 (-0.34 to 0.47)
Self-help (with no or minimal support) vs. attention placebo	0.21 (-0.23 to 0.67)
Guided self-help vs. attention placebo	0.05 (-0.39 to 0.50)
CBT individual (face-to-face) vs. waitlist	**-0.38 (-0.57 to -0.18)**
CBT group (face-to-face) vs. waitlist	-0.36 (-0.80 to 0.07)
BT individual (face-to-face) vs. waitlist	**-0.42 (-0.70 to -0.14)**
Counselling individual (face-to-face) vs. waitlist	-0.26 (-0.59 to 0.08)
Motivational interviewing vs. waitlist	**-0.31 (-0.53 to -0.10)**
Self-help (with no or minimal support) vs. waitlist	**-0.16 (-0.30 to -0.02)**
Guided self-help vs. waitlist	**-0.32 (-0.48 to -0.16)**
CBT group (face-to-face) vs. CBT individual (face-to-face)	0.02 (-0.43 to 0.45)
BT individual (face-to-face) vs. CBT individual (face-to-face)	-0.04 (-0.28 to 0.20)

Counselling individual (face-to-face) vs. CBT individual (face-to-face)	0.12 (-0.18 to 0.42)
Motivational interviewing vs. CBT individual (face-to-face)	0.07 (-0.13 to 0.26)
Self-help (with no or minimal support) vs. CBT individual (face-to-face)	**0.21 (0.06 to 0.38)**
Guided self-help vs. CBT individual (face-to-face)	0.05 (-0.14 to 0.25)
BT individual (face-to-face) vs. CBT group (face-to-face)	-0.06 (-0.55 to 0.44)
Counselling individual (face-to-face) vs. CBT group (face-to-face)	0.10 (-0.44 to 0.63)
Motivational interviewing vs. CBT group (face-to-face)	0.05 (-0.43 to 0.53)
Self-help (with no or minimal support) vs. CBT group (face-to-face)	0.19 (-0.23 to 0.64)
Guided self-help vs. CBT group (face-to-face)	0.03 (-0.41 to 0.48)
Counselling individual (face-to-face) vs. BT individual (face-to-face)	0.16 (-0.13 to 0.46)
Motivational interviewing vs. BT individual (face-to-face)	0.11 (-0.12 to 0.35)
Self-help (with no or minimal support) vs. BT individual (face-to-face)	**0.26 (0.00 to 0.53)**
Guided self-help vs. BT individual (face-to-face)	0.10 (-0.17 to 0.37)
Motivational interviewing vs. counselling individual (face-to-face)	-0.05 (-0.34 to 0.25)
Self-help (with no or minimal support) vs. counselling individual (face-to-face)	0.10 (-0.23 to 0.42)
Guided self-help vs. counselling individual (face-to-face)	-0.06 (-0.40 to 0.26)
Self-help (with no or minimal support) vs. motivational interviewing	0.15 (-0.04 to 0.35)
Guided self-help vs. motivational interviewing	-0.01 (-0.20 to 0.17)
Guided self-help vs. self-help (with no or minimal support)	**-0.16 (-0.31 to -0.01)**

BT: behavioural therapies; CBT: cognitive behavioural therapy; CrI: credible intervals; SMD: standardised mean difference; TAU: treatment as usual

Negative values indicate a favourable outcome of the first treatment class in the comparison. Results where the 95% CrI do not cross the line of no effect are shown in bold.

SUPPLEMENTARY MATERIAL S17: Threshold analysis

In threshold analysis intervention classes based on small numbers considered insufficient for recommendations were excluded: couple interventions (N=8), twelve step group programme (N=11), and SSRIs (N=15). Intervention classes are coded with No Treatment as intervention class 1, TAU is class 2, etc. Note that this means that all intervention class numbers are 1 higher than those displayed in Figure 3 in paper.

Figure S4 (below) shows that the decision to recommend group CBT as the most effective intervention class is robust to changes in the estimates for all contrasts, with the possible exception of the comparison between Behavioural Therapies Individual and CBT Individual. For this contrast the evidence would have to change to give an estimated SMD just below the lower credible limit in favour of Behavioural Therapies Individual for the most effective intervention class to change from Group CBT to Behavioural Therapies Individual.

There were 3 studies comparing Behavioural Therapies Individual and CBT Individual (Korman 2008, Smith 2015, and Thomas 2017). All 3 studies received funding from industry, and had similar risk of bias profiles (including some high risk of bias ratings). Smith 2015 reports complete case data, whereas the other 2 studies report ITT data. The study-specific estimates of SMD from the NMA for Behavioural Therapies Individual relative to CBT Individual were:

- Korman 2008: 0.47 (-0.197, 1.144)
- Smith 2015: -0.15 (-0.550, 0.233)
- Thomas 2017: 0.05 (-0.249, 0.351)

These study-specific estimates are quite heterogeneous, but all are consistent with no effect, and all 95% credible intervals are within the invariant range (-0.74, 9.71). It is unlikely that the true estimate would be outside of all of the study-specific credible intervals.

The results of the threshold analysis suggest that the recommendation made based on the NMA results is robust to potential changes in the evidence. If there were a large change in the estimate for Behavioural Therapies Individual vs CBT Individual, then the most effective intervention class would change to Behavioural Therapies Individual, but this would only occur if the estimate were substantially lower than lower limits of the 95% credible intervals from the 3 existing studies making this comparison.

Figure S4. Threshold analysis for Group CBT being the most effective class for SMD in change from baseline in symptom severity

Contrast	Mean	95% Credible Interval	Invariant Interval		
7 vs. 5	−0.02	(−0.71, 0.66)	7	(−0.74, 9.71)	5
6 vs. 1	−1.09	(−1.75, −0.44)	–	(NT, 0.21)	7
6 vs. 4	−2.04	(−2.70, −1.41)	–	(NT, −0.71)	7
10 vs. 5	0.57	(0.11, 1.04)	10	(−10.58, 2.06)	5
10 vs. 3	−0.04	(−0.73, 0.66)	10	(−70.97, 1.66)	3
9 vs. 8	0.14	(−0.89, 1.18)	9	(−25.95, 1.91)	8
8 vs. 5	0.12	(−0.88, 1.12)	8	(−1.68, 23.05)	5
11 vs. 10	−0.07	(−0.45, 0.30)	11	(−2.33, 8.11)	10
8 vs. 7	0.15	(−0.92, 1.21)	8	(−2.15, 4.56)	7
5 vs. 1	−0.57	(−1.07, −0.06)	5	(−2.92, 12.61)	1
10 vs. 4	−0.95	(−1.34, −0.57)	7	(−3.42, 6.01)	4
9 vs. 6	0.79	(0.07, 1.53)	7	(−1.69, NT)	–
9 vs. 7	0.29	(−0.50, 1.09)	9	(−19.86, 2.98)	7
10 vs. 1	0.01	(−0.36, 0.38)	10	(−2.98, 2.86)	1
11 vs. 2	−0.22	(−1.02, 0.58)	11	(−17.48, 2.78)	2
11 vs. 4	−1.02	(−1.47, −0.59)	11	(−4.19, 14.68)	4
9 vs. 1	−0.30	(−0.84, 0.24)	9	(−3.59, 9.51)	1
9 vs. 5	0.27	(−0.27, 0.81)	9	(−5.14, 3.57)	5
5 vs. 2	−0.72	(−1.53, 0.08)	5	(−8.14, 2.62)	2
11 vs. 3	−0.11	(−0.84, 0.62)	11	(−19.50, 3.27)	3
5 vs. 4	−1.52	(−2.09, −0.98)	5	(−5.89, 40.10)	4
11 vs. 9	0.24	(−0.34, 0.81)	11	(−8.88, 6.03)	9
9 vs. 2	−0.46	(−1.30, 0.38)	9	(−17.28, 5.41)	2
10 vs. 9	0.31	(−0.21, 0.83)	10	(−23.23, 7.53)	9
13 vs. 6	0.71	(−0.71, 2.12)	5	(−14.82, NT)	–
13 vs. 4	−1.34	(−2.84, 0.13)	–	(NT, 15.19)	7

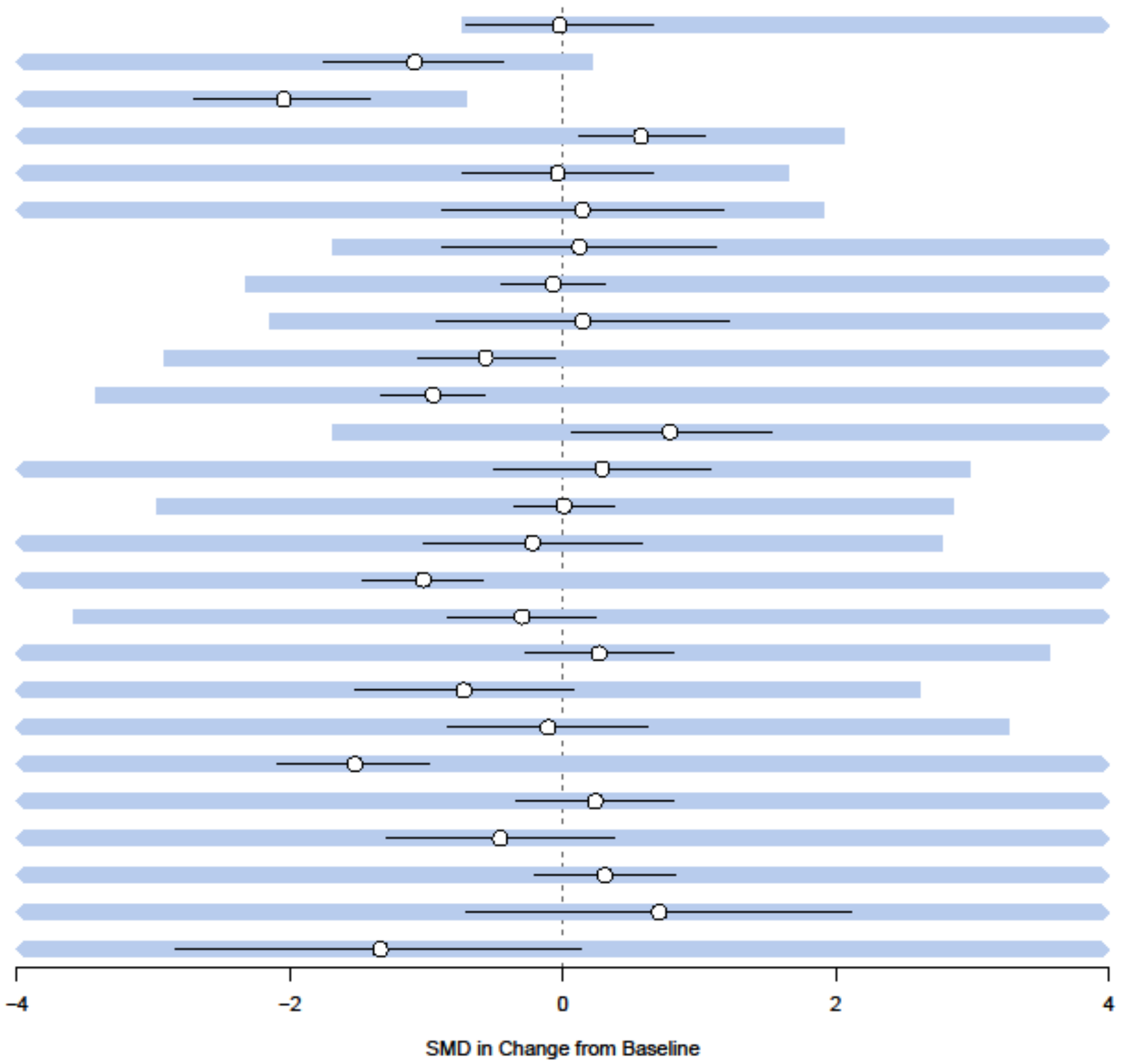

○ Mean — 95% Credible Interval ▬ Invariant Interval

The NMA estimates and 95% credible intervals are shown by the black lines. The blue shaded areas show the invariant interval where the most effective intervention class does not change, and the intervention that would become the most effective class is indicated by the figures either side of the invariant interval. The pink area indicates where the most effective class changes within the credible limits of the NMA estimates. Intervention codes are 1 higher than those given in Figure 3 in paper.

SUPPLEMENTARY MATERIAL S18: Results of the original NMA, undertaken to inform the development of the NICE guideline on gambling-related harms

Gambling symptom severity results – full dataset: posterior standardised mean difference (SMD) of all treatments versus no treatment. ORIGINAL NMA [2023]

Treatment class	N rand	K arms	SMD vs no treatment (mean, 95% CrI)	Rank (mean, 95% CrI)
CBT group	121	6	**-1.08 (-1.82 to -0.35)**	2.20 (1 to 6)
CBT individual	592	17	-0.54 (-1.11 to 0.04)	4.71 (2 to 8)
Behavioural therapies individual	136	3	-0.57 (-1.49 to 0.35)	4.87 (1 to 11)
Couple interventions	8	1	-0.48 (-2.37 to 1.42)	6.14 (1 to 14)
Counselling individual	76	1	-0.42 (-1.64 to 0.80)	6.15 (1 to 13)
SSRIs	15	1	-0.44 (-2.05 to 1.16)	6.25 (1 to 14)
Twelve step group programme	11	1	-0.38 (-2.02 to 1.23)	6.63 (1 to 14)
Motivational interviewing	303	5	-0.29 (-0.90 to 0.32)	6.77 (3 to 11)
Guided self-help	644	11	-0.10 (-0.75 to 0.54)	8.31 (4 to 13)
No treatment	681	9	Reference	9.33 (5 to 13)
Attention placebo	179	2	0.12 (-0.92 to 1.18)	9.73 (3 to 14)
Self-help (with no or minimal support)	1616	22	0.07 (-0.38 to 0.52)	9.98 (6 to 13)
TAU	153	3	0.16 (-0.81 to 1.13)	10.16 (4 to 13)
Waitlist	461	13	**1.05 (0.46 to 1.65)**	13.78 (12 to 14)

CBT: cognitive behavioural therapy; CrI: credible intervals; K arms: number of arms; N rand: number randomised; SMD: standardised mean difference; SSRIs: selective serotonin reuptake inhibitors; TAU: treatment as usual
Treatment classes ordered from best to worst, according to mean rankings. Negative effect values indicate a favourable outcome compared with no treatment. Results where 95% CrI do not cross the no effect line are shown in bold. It is noted that in this original NMA, the 95%CrI of individual CBT marginally cross the line of no effect versus no treatment (-1.11 to 0.04).

Gambling frequency results – full dataset: posterior standardised mean difference (SMD) of all treatments versus no treatment ORIGINAL NMA [2023]

Treatment class	N rand	K arms	SMD vs no treatment (mean, 95% CrI)	Rank (mean, 95% CrI)
TAU	111	1	**-0.42 (-0.69 to -0.14)**	3.186 (1 to 8)
Behavioural therapies individual	98	2	**-0.41 (-0.68 to -0.13)**	3.336 (1 to 8)
CBT individual	331	7	**-0.36 (-0.55 to -0.18)**	4.012 (1 to 7)
Attention placebo	39	1	-0.37 (-0.86 to 0.08)	4.405 (1 to 11)
CBT group	30	2	-0.34 (-0.77 to 0.10)	4.757 (1 to 11)
Guided self-help	608	9	**-0.31 (-0.47 to -0.13)**	5.284 (2 to 8)
Motivational interviewing	290	6	**-0.30 (-0.49 to -0.09)**	5.569 (2 to 8)
Counselling individual	76	1	-0.24 (-0.57 to 0.08)	6.611 (1 to 11)
Self-help (with no or minimal support)	1526	18	**-0.15 (-0.25 to -0.04)**	8.237 (6 to 9)
No treatment	592	7	Reference	10.24 (9 to 11)
Waitlist	401	8	0.01 (-0.15 to 0.19)	10.37 (9 to 11)

CBT: cognitive behavioural therapy; CrI: credible intervals; K arms: number of arms; N rand: number randomised; SMD: standardised mean difference; TAU: treatment as usual
Treatment classes ordered from best to worst, according to mean rankings. Negative effect values indicate a favourable outcome compared with no treatment. Results where 95% CrI do not cross the no effect line are shown in bold.
